# A Biomimetic Study of the Behavior of *N*‑Cyclopropyl-Based Single Electron Transfer Probes in the Context of Monoamine Oxidase-Catalyzed Oxidations

**DOI:** 10.1021/acs.joc.5c02528

**Published:** 2026-01-10

**Authors:** Nathan Price, Bradley Engels, Paul Venturo, Jonathan Sánchez González, Thomas Robbins, Joseph Barton, Neal Castagnoli, James M. Tanko

**Affiliations:** Department of Chemistry, 1757 Virginia Polytechnic Institute and State University, Blacksburg, Virginia 24060, United States

## Abstract

Monoamine oxidase-A and -B are important flavoenzymes involved in the oxidative metabolism of various biogenic amines. Mechanisms including polar/nucleophilic, hydride transfer, and single electron transfer (SET) have been proposed for the initial steps of the catalytic mechanism. The most compelling evidence for the latter comes from the observed inhibitory behavior of *N*-cyclopropyl compounds. Enzyme inactivation presumably occurs when the primary radical portion of the distonic radical cation, resulting from cyclopropyl ring opening, couples to the enzyme. Previously, we hypothesized that the unique substrate behavior of 1,4-disubstituted-1,2,3,6-tetrahydropyridinyl systems was attributable to certain structural features which activate the SET pathway. In the present work, the oxidation of several *N*-cyclopropyl derivatives of MPTP in a biomimetic system (3MLF/*hν*) is reported. Calculations suggest that the barrier to ring opening may not be as low as assumed, and experiments show the process is reversible. The results also suggest that the ring-opened (distonic) radical cation may disrupt the active site through radical coupling, hydrogen atom abstraction, or through reaction with O_2_ that can lead to reactive oxygen species. Hydrolysis of an intermediate iminium moiety leads to the production of low molecular weight aldehydes, which may also provide a pathway for enzyme inactivation.

## Graphical abstract

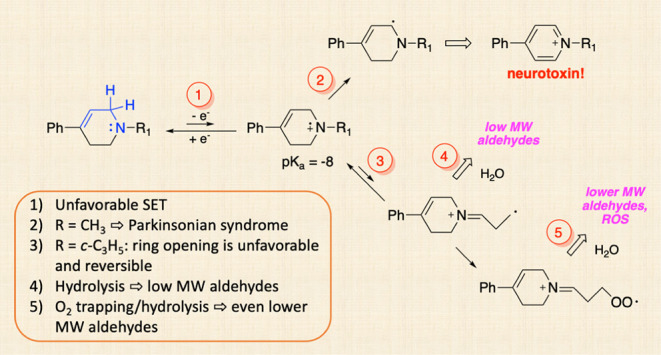

## Introduction

Monoamine oxidase (MAO) exists in 2 forms, MAO-A and MAO-B. These are mitochondrial flavoenzymes that catalyze the oxidative deamination of biogenic amines including serotonin (**1**), dopamine (**2**), and epinephrine (**3**, Figure 1a).^1,2^ Tetrahydropyridines such as 1-methyl-4-phenyl-1,2,3,6-tetrahydropyridine (**MPTP**, Figure 1a) are xenobiotics and unique MAO substrates. In humans and other mammals, injection of this compound leads to the irreversible destruction of dopaminergic nigrostriatal neurons and to the onset of a Parkinsonian syndrome. MAO-B catalyzes the α-carbon oxidation of **MPTP** to form the corresponding 1,2-dihydropyridinium species **MPDP** ^+^ that undergoes a second 2-electron oxidation to give the 1-methyl-4-phenylpyridinium species **MPP** ^+^, the proposed ultimate neurotoxin.^3^

**Figure 1:**
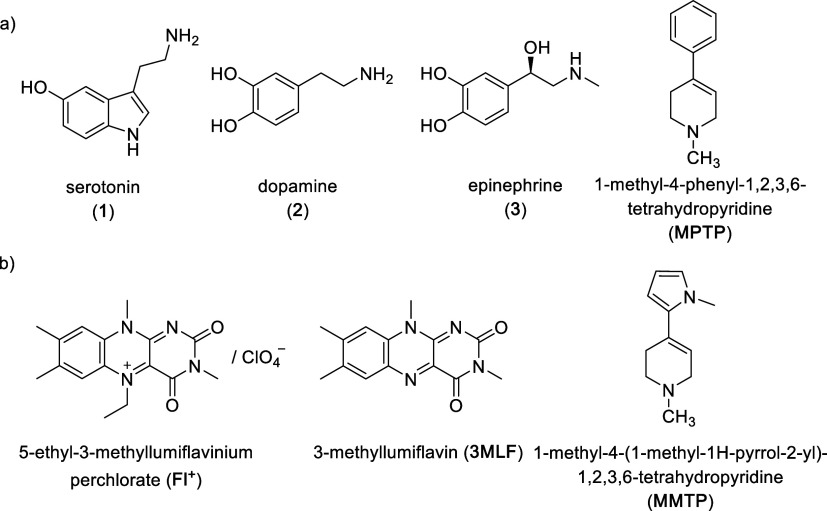
(a) Common primary and secondary biogenic amines oxidized by MAO-B are shown alongside 1-methyl-4-phenyl-1,2,3,6-tetrahydropyridine (**MPTP**). **MPTP** and related tetrahydropyridines are xenobiotics and the only known 3° amines that are MAO substrates. (b) Biomimetics used to probe MAO’s mechanism of catalysis. **MMTP** is an **MPTP** analog with no reported human toxicity.

Various mechanisms have been suggested to account for the initial stages of MAO-catalyzed oxidations including nucleophilic,^4,5^ hydride transfer^5,6^ and single electron transfer (SET).^7–11^ For the most part, the recent literature has focused almost exclusively on the polar mechanisms (nucleophilic or hydride transfer),^1,12–14^ with fewer reports advocating for SET.^15–17^ However, there is an anomaly in the literature which has only recently been addressed. Generally, tertiary amines are not MAO substrates. In addition, they cannot react via polar mechanisms because of steric constraints at the MAO active site. This holds true for MAO biomimetics as well. Mariano et al. found that the 3-methyllumiflavin (**3MLF**)-promoted oxidation of various benzylamines followed the reactivity order 1° > 2° ≫ 3°.^18^

Yet as noted, **MPTP** and other tetrahydropyridines are the only known tertiary amines that are MAO substrates. Using the biomimetics 5-ethyl-3-methyllumiflavinium perchlorate (**Fl** ^+^)^19^ and 3-methyllumiflavin (**3MLF**),^20^ which mimic the flavin active site in MAO, we recently demonstrated that (unlike other tertiary amines) 1-methyl-4-(1-methyl-1*H*-pyrrol-2-yl)-1,2,3,6-tetrahydropyridine (**MMTP**, a close analog of **MPTP**, Figure 1b) was oxidized by an SET pathway.^19,20^ It is worth noting that **Fl** ^+^ and **3MLF** were the same biomimetics used by Mariano et al. earlier, who found no evidence for SET. The critical difference is that their work did not examine tertiary amines with similar structural features to **MPTP** and derivatives.

In this context, we developed a hypothesis (Figure 2) to explain why tertiary amines such as **MPTP** and related compounds are MAO substrates, while other, fully aliphatic tertiary amines are not: The α-C=C moiety in **MPTP** (and derivatives) dramatically lowers the p*K*_a_ of the corresponding aminyl radical cations, from ca. 8 for a fully aliphatic tertiary amine radical cation such as (CH_3_)_3_N^•+^ to as low as −5 for **MPTP** ^•+^.^19–22^ The neutral radical resulting from this deprotonation is considerably more stable (ca. 15 kcal/mol) than would be the case if the α-C=C were not present.^21^ Thus, although the electron transfer process is a thermodynamically unfavorable equilibrium, it is driven toward products in the context of Le Chatlier’s principle because of an extremely facile deprotonation step. It is interesting to note that the only 3° amine Mariano found to react with **3MLF** possessed a very unusual feature, α-Si­(CH_3_)_3_ moiety vs H as a positively charged leaving group.^18^

**Figure 2:**
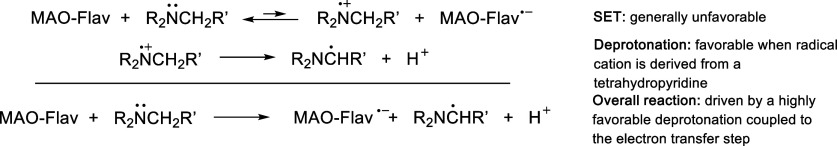
Single electron transfer hypothesis for the MAO catalyzed oxidation of tetrahydropyridines; an unfavorable electron transfer is driven by an extremely favorable deprotonation. Reproduced from reference 20. Available under a CC-BY license. Copyright 2024 N. J. Price, A. Nakamura, N. Castagnoli, and J. Tanko.

Arguably, the most compelling evidence for single electron transfer in MAO-catalyzed oxidations came from the use of single electron transfer probes. SET probes are typically strained, small ring-containing substrates which, in analogy to the classic cyclopropylcarbinyl → homoallyl neutral radical rearrangement, undergo rapid and irreversible ring opening when a radical or radical ion is produced. The general concept behind this approach is outlined in Figure 3 using an *N*-cyclopropyl **MPTP** analog, which is a mechanism-based inactivator of MAO B.^11^ Electron transfer to the flavin moiety in the MAO active site produces an aminyl radical cation and flavin radical anion. Deprotonation of the radical cation (path a) by a base (B:) generates a neutral α-aminoalkyl radical (*k*_*b*_ is the rate constant for this process), ultimately leading to a pyridinium species. When the substrate possesses an *N*-cyclopropyl group or other strained moiety, a competing ring opening pathway (path b) is introduced, where *k*_*o*_ is the unimolecular rate constant for ring opening. It has been suggested that inactivation results because the resulting ring opened (distonic) radical cation becomes attached to the enzyme active site.^7^ The rate constant for cyclopropane ring opening is critical because for ring opening to occur and SET to be detected, *k*_*o*_ ≫ *k*_*b*_ [B:].

**Figure 3:**
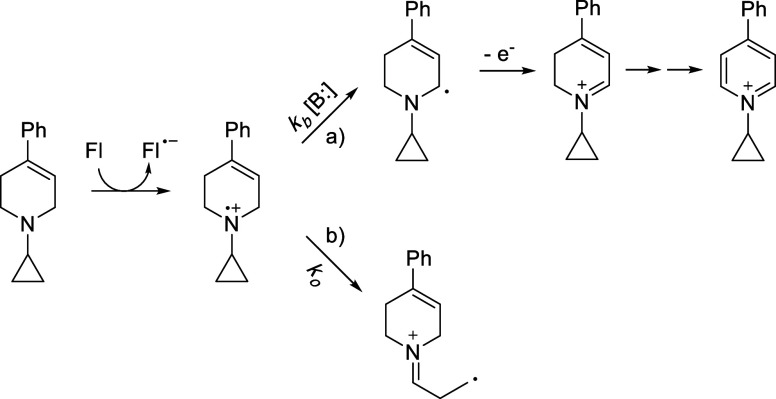
Use of an electron transfer probe to provide evidence for single electron transfer in the MAO-catalyzed oxidation of **MPTP**. Electron transfer to the flavin moiety in the MAO active site produces an aminyl radical cation. (a) Deprotonation and subsequent oxidation steps lead to pyridinium ion formation; *k*_*b*_ is the rate constant for deprotonation and B: is the base. (b) The presence of an *N*-cyclopropyl group introduces a competing pathway that leads to a ring-opened distonic radical cation; *k*_*o*_ is the rate constant for ring opening. The ring-opened distonic radical cation is believed to disrupt the enzyme active site. In order for this approach to be successful in detecting a single electron transfer event, ring opening must be rapid (*k*_*o*_ ≫ *k*_*b*_[B:]) and irreversible. Reproduced from reference 20. Available under a CC-BY license. Copyright 2024 N. J. Price, A. Nakamura, N. Castagnoli, and J. Tanko.

Pioneering work by Silverman et al. used various *N*-cyclopropyl, *N*-cyclobutyl, and other strained compounds to probe the MAO catalytic mechanism (Figure 4a).^7,8,23^ These substrates proved to be MAO inhibitors, covalently attaching to the enzyme in a 1:1 stoichiometry and producing ring-cleaved metabolites. Using various *N*-cyclopropyl derivatives of **MPTP** (and related compounds), Castagnoli et al. extended this approach (Figure 4a) to learn more about the MAO-catalyzed oxidation of **MPTP**. Many of these compounds proved to be mechanism-based, irreversible inhibitors of MAO, consistent with an SET pathway.^10,11,24^

**Figure 4:**
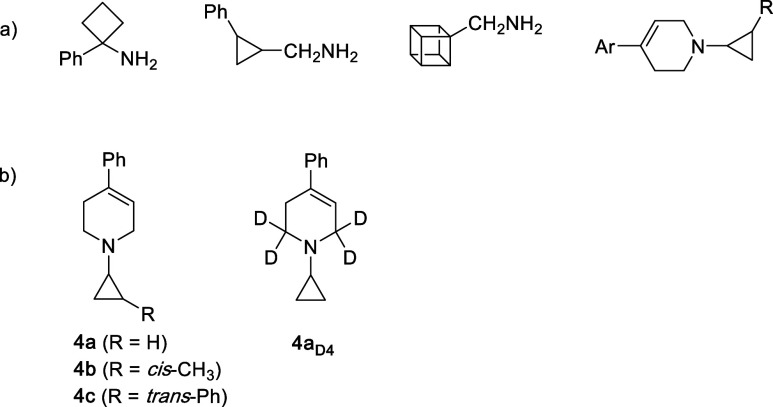
(a) Mechanism-based MAO inhibitors used by Silverman^7,8,23^ and Castagnoli^10,11,24^ to probe for single electron transfer. (b) *N*-Cyclopropyl analogs of **MPTP** used in this study.

In an attempt to reconcile the evidence for SET presented by Castagnoli and Silverman with the nucleophilic mechanism, Mariano et al. demonstrated that flavin-substrate adducts may also be produced by nonradical (polar) pathways (using **Fl** ^+^ as a biomimetic).^25^ Again though, none of the substrates studied had the critical α-C=C which we have suggested leads to unique **MPTP** chemistry.

The present work examines the chemistry of several 3° *N*-cyclopropyl compounds related to **MPTP** in a biomimetic system (Figure 4b) with a focus on the chemistry that occurs after single electron transfer. The principal objectives are to determine whether ring opening occurs, and if so, to identify the resulting products. We hypothesize that these results will provide new insight into the mechanism of MAO-catalyzed oxidations, especially in the context of enzyme inactivation. As noted, for the SET probe approach to succeed, ring opening should be rapid and irreversible, and this study will test these assumptions.

## Results and Discussion

The reactions of **3MLF** with *N*-cyclopropyl MPTP derivatives **4** (**a**–**c**) were studied under anaerobic and aerobic conditions. The reactions were monitored by ^1^H NMR. The solvent was CD_3_CN. This solvent contained a small amount of H_2_O which proved critical in the analysis of the outcomes of these experiments. The reactions were typically performed on a 2–5 mM scale. The ratio of **3MLF** to substrate was as close to 1:1 as feasible. NMR spectra were recorded at t = 0 and after the reaction had gone to completion (20–24 h).

For reactions conducted under anaerobic conditions, the final NMR spectra were obtained before and after exposure to air and, in some cases, benzene was added as an internal standard. The reaction mixtures were further analyzed by LC-MS/MS. Volatile products (low molecular weight aldehydes) were separated by a room temperature trap-to-trap distillation using a vacuum line and analyzed by NMR. A reviewer commented favorably that these complex reaction mixtures were analyzed as fully as possible given the constraints, e.g., the small scale of the reactions dictated by the limited availability of the tetrahydropyridine derivatives, and concerns about their potential neurotoxicity.

Unlike our earlier results for the reaction of **3MLF** with **MMTP**,^20^ the reactions of all the *N*-cyclopropyl MPTP derivatives with **3MLF** required visible light (50 W tungsten light bulb). There was no significant thermal reaction detected within a 24 h period in the dark. The low energy excited states of **3MLF** have a redox potential (*E*_1/2_(triplet) = 2.1 V; *E*_1/2_(singlet) = 2.6 V) that makes them a much more potent oxidant than the ground state (*E*_1/2_ = 0.04 V).^9,26^

A detailed analysis of the results for the reaction of **3MLF** and **4a** is provided below. Results for **4b** and **4c** are available in the Supporting Information. Molecular orbital calculations were performed to support and amplify these findings.

The first issue to address regarding these results is why do these reactions require light? Earlier work demonstrated a thermal reaction between **3MLF** and **MMTP**.^20^ We believe the difference is attributable to the fact that **MMTP** is much more easily oxidized than **MPTP** and any of its *N*-cyclopropyl derivatives. To test this, calculations were performed to assess energetics of a hypothetical electron transfer between the 1,2,3,6-tetrahydropyridinyl radical cation and **MPTP**, **MMTP**, and related compounds. The results, summarized in Table 1, reveal that **MMTP** is more readily oxidized than **MPTP** by 500 mV. The results also show that the phenyl group in **MPTP** has little effect on the oxidation energetics (Δ*E* < 100 mV, comparing **MPTP** to 1,2,3,6-tetrahydropyridine). Thus, **MPTP** derivatives require a substantially more potent oxidant (i.e., the excited state of **3MLF**)^26^ to drive the electron transfer process.

**Table 1:**
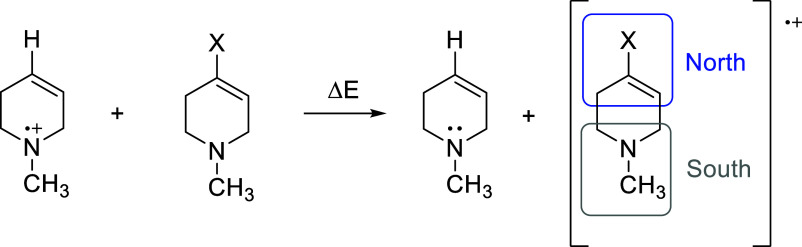
Energetics of Electron Transfer between 1,2,3,6-Tetrahydropyridine Radical Cation and Itself, MPTP, and MMTP; Spin and Charge Densities Associated with Different Regions of the Resultant Radical Cations Are Shown

	CAM-B3LYP-/def2-TZVP^a^				M06-2X/cc-PVTZ^a^			
X	Δ*E* (mV)	Region^b^	Charge	Spin Density	Δ*E* (mV)	Region^b^	Charge	Spin Density
*N*-methylpyrrole (s-cis)^c^	–570	N	0.83	0.96	–551	N	0.69	0.96
		S	0.17	0.04		S	0.31	0.04
*N*-methylpyrrole (s-trans)^c^	–622	N	0.83	0.97	–612	N	0.68	0.97
		S	0.17	0.03		S	0.32	0.03
phenyl	–94	N	0.21	0.06	–79	N	0.16	0.07
		S	0.79	0.94		S	0.84	0.93
H	0	N	0.20	0.06	0	N	0.19	0.06
		S	0.80	0.94		S	0.81	0.94

a Geometry optimizations were performed using M062X/6-31G­(d) followed by single point calculations at the level indicated.

b N and S refer to the northern and southern regions of the radical cations.

c s-*cis* and s-*trans* refer to the two planar conformations of the *N*-methyl on the pyrrole ring relative to the C=C of the tetrahydropyridine ring. (The s-*cis* conformation places the methyl group closer to the C=C and vice versa.).

The reason for this dramatic difference becomes readily apparent by examining the charge and spin density in the radical cations. For **MMTP**, the spin and positive charge are mostly confined to the northern region of the species, i.e., in the pyrrole ring. The opposite holds true for **MPTP** as most of the charge and spin are restricted to the tetrahydropyridine ring in the southern region. Thus, the electronic structures of **MPTP** ^•+^ and **MMTP** ^•+^ are very different. Although unexpected, this result makes sense. The ionization potentials of *N*-methylpyrrole, *N*-methyl-1,2,3,6-tetrahydropyridine, and benzene are 8.0, 8.7, and 9.2 eV, respectively.^27^ *N*-Methypyrrole is the most easily oxidized of the three compounds. Thus, the nitrogen lone pair on the tetrahydropyridine moiety of **MPTP** is most easily oxidized, whereas the pyrrole ring in **MMTP** is more readily oxidized. However, despite the difference in the electronic structures and stability of these two radical cations, both, upon deprotonation, lead to radicals of comparable structure and stability (Figure 5). The α-C–H BDEs of **MPTP** and **MMTP** are nearly identical, 73 and 74 kcal/mol, respectively.^21^

**Figure 5:**
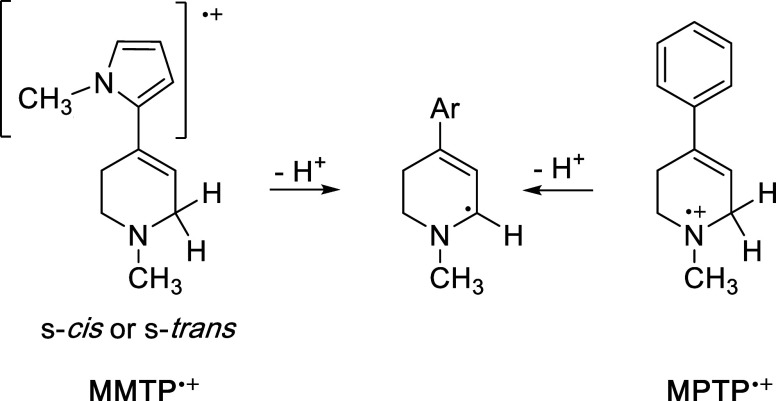
Although the electronic structures of **MMTP** ^•+^ and **MPTP** ^•+^ are different, both lead to radicals of comparable stability upon loss of a proton based upon the α–C-H BDEs of the **MPTP** and **MMTP**.

The ^1^H NMR spectrum for the reaction of a 1:1 mix of **3MLF** and **4a** after 20 h of irradiation under anaerobic conditions is shown in Figure 6. Figure 6a is the spectrum obtained after the reaction is complete and still under anaerobic conditions. Most of the peaks in this spectrum can be assigned to either unreacted substrate (**4a**) or products (**5a**–**8a**), based upon direct comparison to authentic spectra. Most notably though, no peaks associated with **3MLF** are observed and there are a few unassigned peaks, presumed to arise from reaction intermediates (noted in dotted rectangular boxes). When the NMR tube was opened and exposed to air, peaks attributable to **3MLF** reappeared while the unknown peaks vanished (Figure 6b). LC-MS/MS analysis of the reaction mixture detected only **4a**, **5a**, **6**, and **3MLF**. Finally, the volatile components of the reaction mixture, propionaldehyde (**7a**) and propenal (**8a**) were separated by high vacuum trap-to-trap distillation at room temperature and their identities were further confirmed by ^1^H NMR analysis. In response to a reviewer comment, it should be noted that the volatile aldehydes could not be resolved from solvent by GC or HPLC. However, the fact that they were in fact volatile (separated by ambient temperature vacuum distillation), and the NMR spectra matched authentic samples leaves no ambiguity as to the assignment.

**Figure 6:**
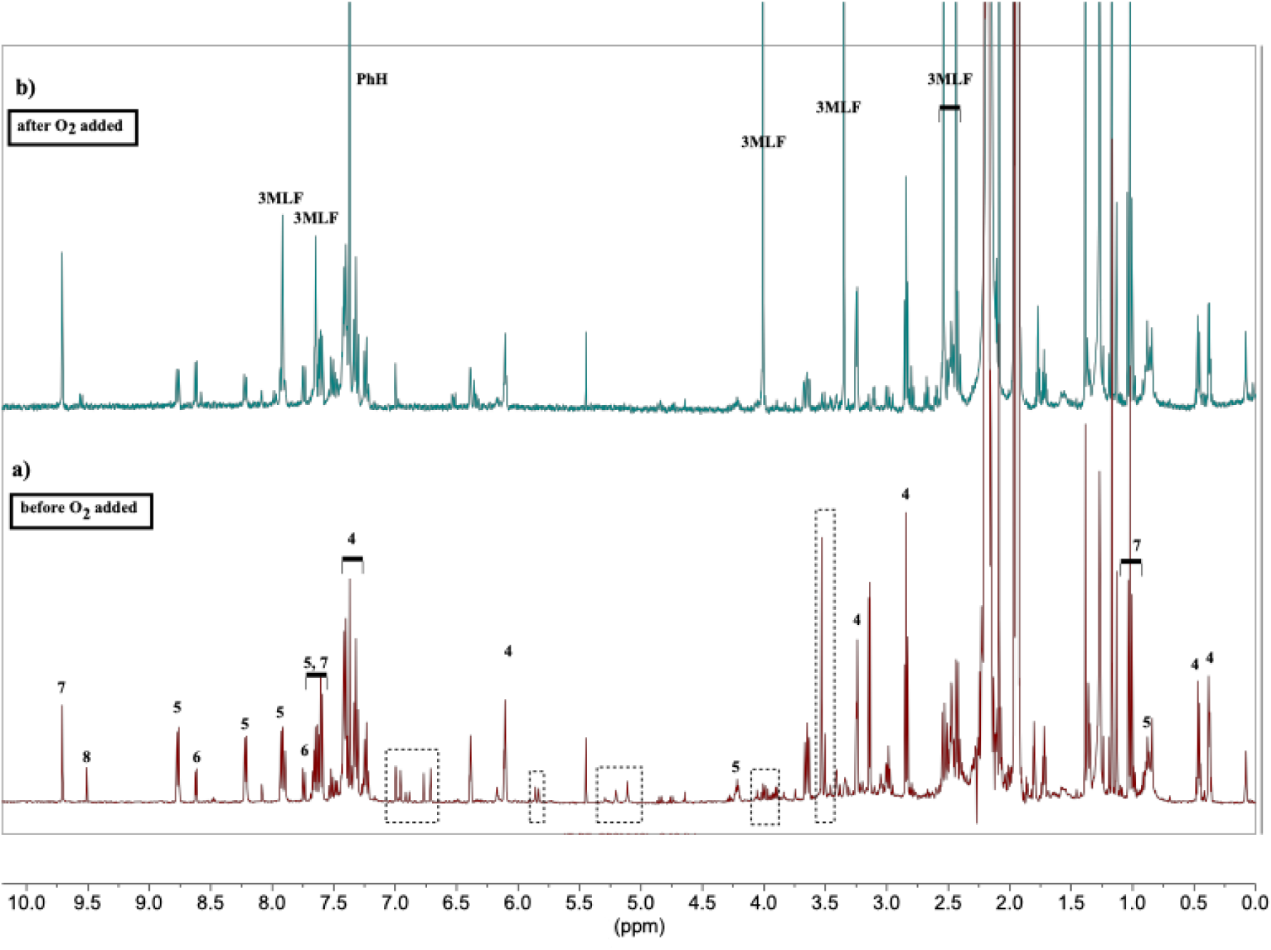
^1^H NMR analysis of the anaerobic reaction of **3MLF** and **4a** (1:1) in CD_3_CN. (a) The reaction mixture after 20 h of irradiation (anaerobic). The identity of the major peaks are noted, and correspond to compounds **5a**–**8a**. No peaks associated with **3MLF** are observed, but there are a few unassigned peaks (indicated with dotted boxes). (b) This illustrates the changes observed when the NMR tube is opened and exposed to air. Most of the assigned peaks remain (though there are some changes in intensity), the unassigned peaks vanish, and peaks corresponding to **3MLF** (re)­appear.

The results for the reactions of **3MLF** with **4b** and **4c** were similar and are summarized in Table 2. In all cases, there was approximately 50% unreacted substrate, which is consistent with the reaction stoichiometry (vide infra). Two products derived from the tetrahydropyridine moiety of the substrate were observed, a pyridinium species which maintained a closed cyclopropane ring (**5**), and 4-phenylpyridine (**6**).

**Table 2:**
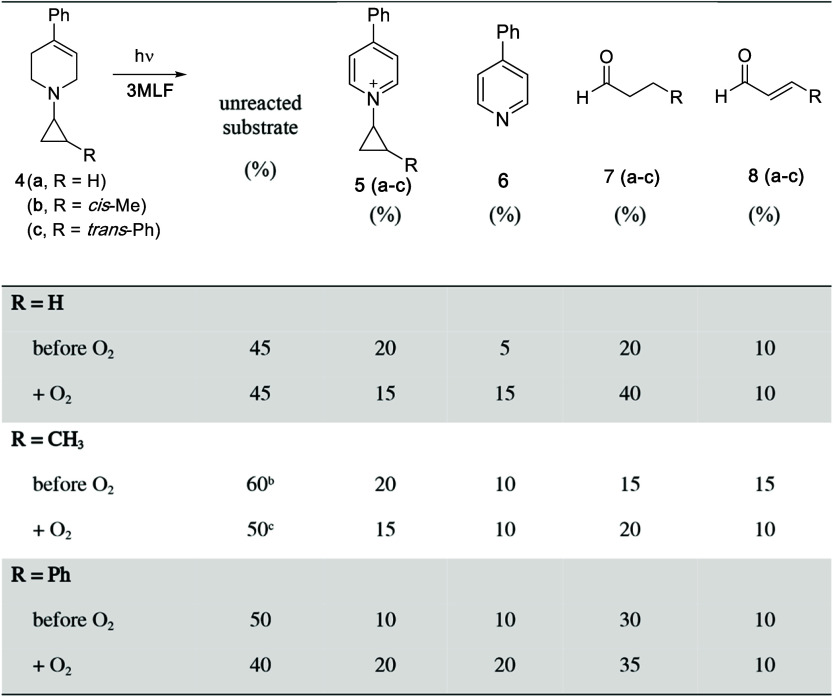
Results of Reactions of *N*-Cyclopropyl Compounds **4**(**a**–**c**) with 3MLF^d^

a Regarding mass balance: Because the substrates fragment into two products, total mass balance can be based on the cyclopropyl group (% **4** + % **5** + % **7** + % **8** ≤ 100%), or the tetrahydropyridine moiety (% **4** + % **5** + % **6** ≤ 100%).

b 70% *trans*; 30% *cis*.

c 65% *trans*; 35% *cis*.

d The % composition[Table-fn tbl2fn1] of the reaction mixture relative to starting material at *t* = 19 h was Determined by 1H NMR. The starting ratio of **3MLF**: **1­(a–c)** was 1:1; the reaction was conducted anaerobically. Corresponding spectra can be found in the Supporting Information.

Compounds **6**–**8** likely arise from cyclopropane ring opening, as shown in Figure 7. Ring opening of radical cation **4** ^•+^ leads to distonic radical cation **9** ^•+^, which can undergo disproportionation leading to **10** and **11** (precursors to **7** and **8**). Alternatively, there is also another possible H atom donor present, namely the substrate itself. The CH_2_ moiety in the substrate that is α-both to nitrogen and the C=C has an unusually low C–H bond dissociation energy (75 kcal/mol).^21^ Hydrolysis of the iminium species by H_2_O (present in the CD_3_CN NMR solvent) generates the observed aldehydes.

**Figure 7:**
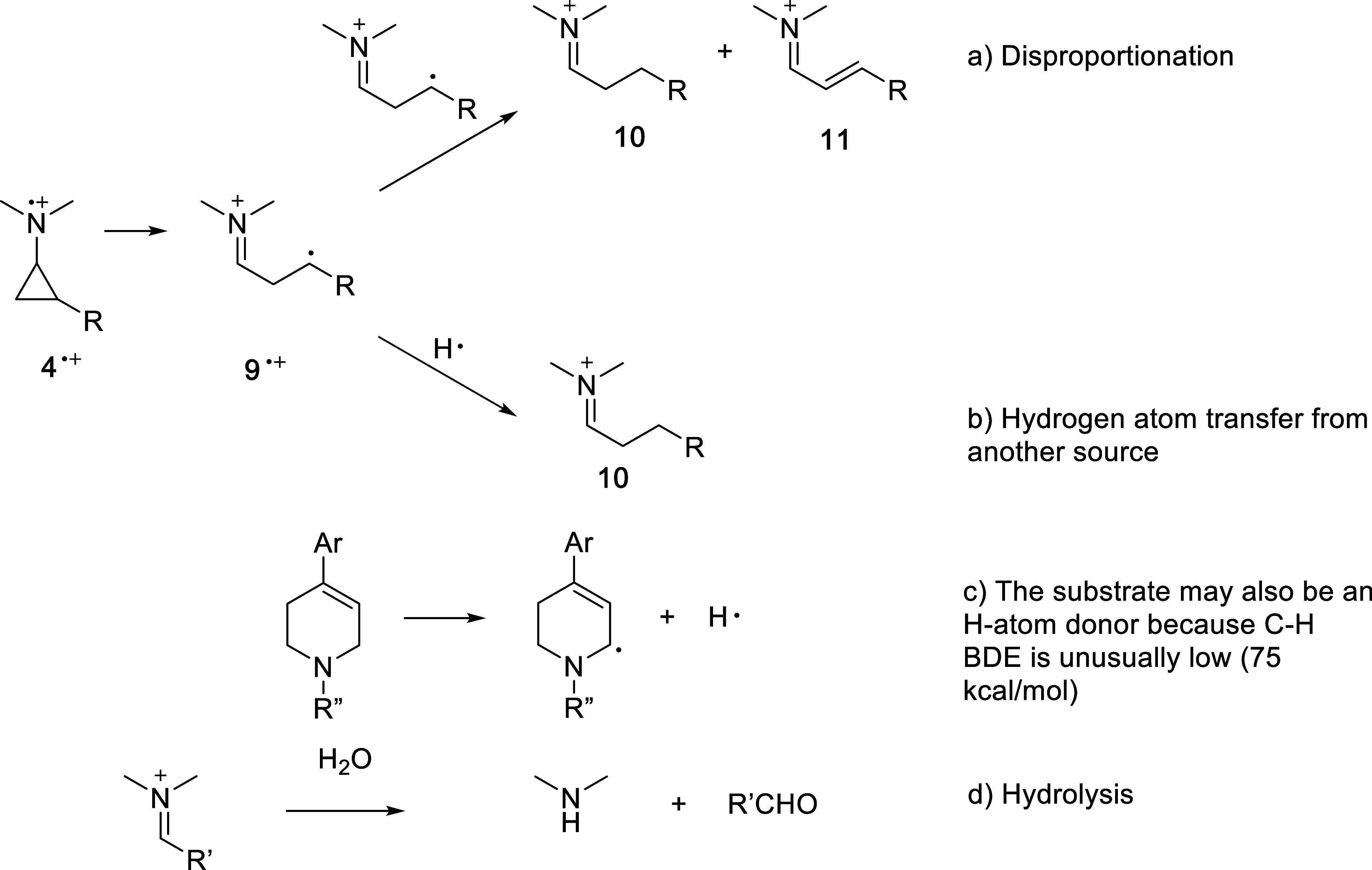
Ring opening of **4** ^•+^ generates distonic radical cation **9** ^•+^. (a) Disproportionation of **9** ^•+^ leads to **10** and **11**, which are precursors to the observed aldehydes. (b) Hydrogen atom transfer to **9** ^•+^ from another source may also lead to **10**. (c) The substrate has an exceptionally weak C–H bond and may be a hydrogen atom source. (d) Hydrolysis of iminium species **10** and **11** leads to the aldehyde products.

To further probe whether the starting substrate was a source of the hydrogen atom leading to aldehyde **7a**, the reaction of **3MLF** and deuterium labeled substrate (**4a**_**D4**_) was examined. The reaction was conducted as described above for **4a**, i.e., 1:1 stoichiometric ratio under anaerobic conditions, and the propionaldehyde product was isolated via room temperature high vacuum trap-to-trap distillation. If the starting material were indeed the source of hydrogen, then the methyl group in propionaldehyde would be expected to be deuterated under these reaction conditions.

For CH_3_CH_2_CHO, the methyl group appears at δ = 1.03 ppm as a triplet (J = 9.7 Hz, coupled to the adjacent CH_2_). For CH_2_DCH_2_CHO, the hydrogens on the methyl would be coupled both to the adjacent CH_2_, and the α-deuterium atom, leading to a multiplet of 9 peaks in accordance with the 2nI + 1 rule.^28^ The coupling constant for H/D coupling is readily calculated based upon the ratio γ_H_/γ_D_, where γ refers to the gyromagnetic ratio of H vs D (ca. 6.5).^28^ The normal coupling constant for hydrogens attached to the same carbon is 12–16 Hz, which means the expected H/D coupling constant would be 1.8–2.5 Hz. In short, for CH_2_D, the normal triplet of propionaldehyde would be replaced by a triplet of triplets. In addition, the replacement of H by D would move the^1^H NMR signal slightly upfield.^29^

Figure 8a shows the methyl region of the propionaldehyde distillate obtained from the reaction of **4a**_**D4**_, which is neither a simple triplet nor the nine line pattern described above, leading us to suspect that it was a mixture of CH_3_ and CH_2_D. Figure 8b shows a simulated ^1^H NMR spectrum (methyl region) for a 50:50 mix of CH_3_CH_2_CHO and CH_2_DCH_2_CHO. The simulation parameters for the methyl group of CH_2_DCH_2_CHO (δ = 1.01 with a slight upfield shift relative to CH_3_,^29^ and *J*_H/D_ = 2.5 Hz) are well within reason. This result confirms that there are two pathways for hydrogen atom transfer to produce the propionaldehyde product: hydrogen atom transfer from the substrate and disproportionation of the ring opened radical ions.

**Figure 8:**
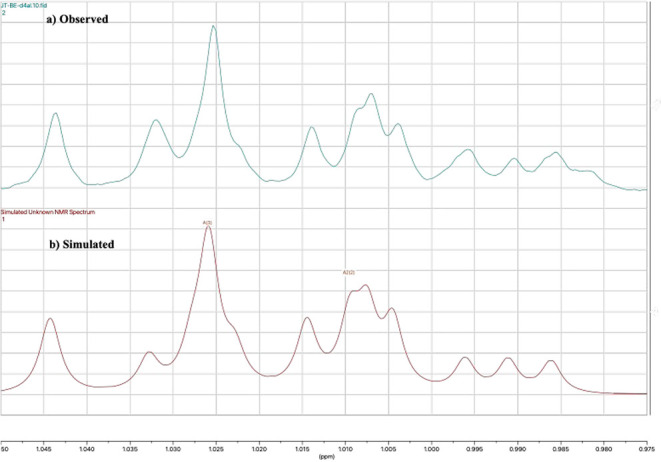
(a) The observed ^1^H NMR spectrum (methyl region) of the propionaldehyde distillate obtained from the reaction of **3MLF** and **4a**_**D4**_ in CD_3_CN. (b) The predicted ^1^H NMR spectrum for a 50:50 mix of CH_3_CH_2_CHO and CH_2_DCH_2_CHO generated using MestReNova, v. 14.1.2–25024 (Mestrelab Research). For CH_3_, δ = 1.03, *J* = 9.7 Hz (coupling to CH_2_). For CH_2_D, δ = 1.01 ppm, *J* = 9.2 Hz (coupling to CH_2_), and *J* = 2.5 Hz (coupling to D).

The detection of hydrogen atom transfer from the substrate was unexpected. Although there is a significant thermodynamic driving force (Δ*H* ^o^ = −23 kcal/mol, based upon C–H bond strengths), hydrogen atom transfers between carbon atoms tend to have a large activation energy.^30^ In principle, the process may occur intermolecularly, or intramolecularly via a 6-membered ring transition state. The intramolecular pathway has been suggested previously based upon results obtained from a mass spectrometric study of the ring opening pathway,^10^ although those results can also be explained invoking the intermolecular pathway. These experiments and the available substrates do not allow us to unambiguously resolve this issue, though for reasons outlined below, the overall results seem more consistent with an intermolecular process.

The rate constants for ring opening of these radical cations are expected to be **4c** ^•+^ ≫ **4b** ^•+^ > **4a** ^•+^, attributable to stabilization of the radical portion of the ring opened distonic radical cations, e.g., Ph ≫ Me > H.^31^ Earlier work using a novel EC/ESI-MS, which involves coupling an electrochemical cell to an electrospray ionization mass spectrometer, provided results in support of this expected trend. Moreover, based upon molecular orbital calculations (UHF/6-31G*), these authors suggested that for **4c**, ring opening and electron transfer might be concerted (i.e., **4c** ^•+^ does not reside at a potential energy minimum.)^32^

To obtain more quantitative information about the barrier to ring opening of these radical cations, molecular orbital calculations were performed at various levels. Although the numbers varied somewhat with method and basis set, the trends were consistent. Table 3 summarizes the results obtained specifically using M06-2X/DEF2-TZVP//M06-2X/CC-PVDZ.^33^ Results from other methods are provided in the Supporting Information. In the case of **4b** ^•+^, although the *cis* isomer was used in this study, results for the *trans* isomer were included for reasons that will become apparent.

**Table 3:**
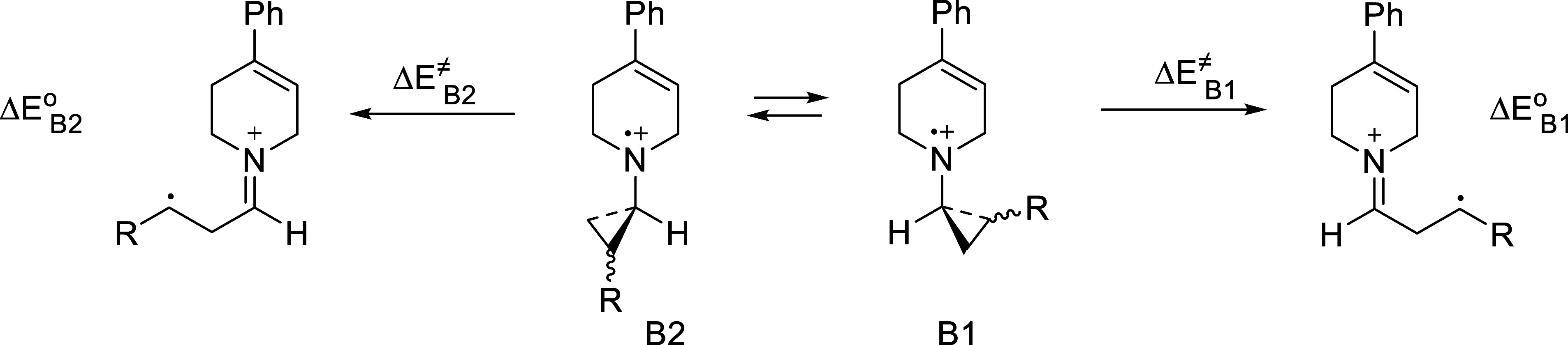
Results Obtained from Molecular Orbital Calculations^a^ Pertaining to the Barrier (Δ*E* ^≠^)­^b^ and Energetics (Δ*E* ^o^)­^c^ of Ring Opening for Radical Cations Generated from **4**(**a**–**c**)

Structure	Δ*E* ^≠^_B2_ (kcal/mol)	Δ*E* ^o^_B2_ (kcal/mol)	Δ*E* ^≠^_B1_ (kcal/mol)	Δ*E* ^o^_B1_ (kcal/mol)
**4a** ^•+^	6.6	3.3	5.8	3.2
*cis*-**4b** ^•+^	1.5	–2.5	2.5	–0.4
*trans*-**4b** ^•+^	3.7	2.1	3.2	1.8
*trans*-**4c** ^•+^	n/a^d^	n/a^d^	n/a^d^	n/a^d^

a Geometry optimizations were performed at the M06-2X/CC-PVDZ level followed by a single point calculation at the M06-2X/DEF2-TZVP to determine the energies.

b Δ*E* ^‡^ = *E*(transition state) – *E*(reactant).

c Δ*E* ^o^ = *E*(ring-opened product) – E­(reactant).

d Neither conformation B1 nor B2 was found to reside at potential energy minimum. All attempts to optimize the ring closed form of **4c** ^•+^ at any level of theory resulted in a ring-opened structure.

As reported in earlier work using less robust methods and basis sets,^32^ a stationary point could not be found for the radical cation generated from **4c**, suggesting that **4c** ^•+^ may not exist at a potential energy minimum. This was further probed by constructing an energy profile, varying the C–C bond distance from 2.3 to 1.5 Å (typical C–C bond length in a cyclopropane), starting from either conformation of the ring-opened form of **4c** ^•+^ (Figure 9). The curves suggest that the interaction between C1 and C2 is repulsive at all distances, consistent with the notion that electron transfer and ring opening are concerted for **4c** ^•+^ (concerted dissociative electron transfer, cDET).^31^

**Figure 9:**
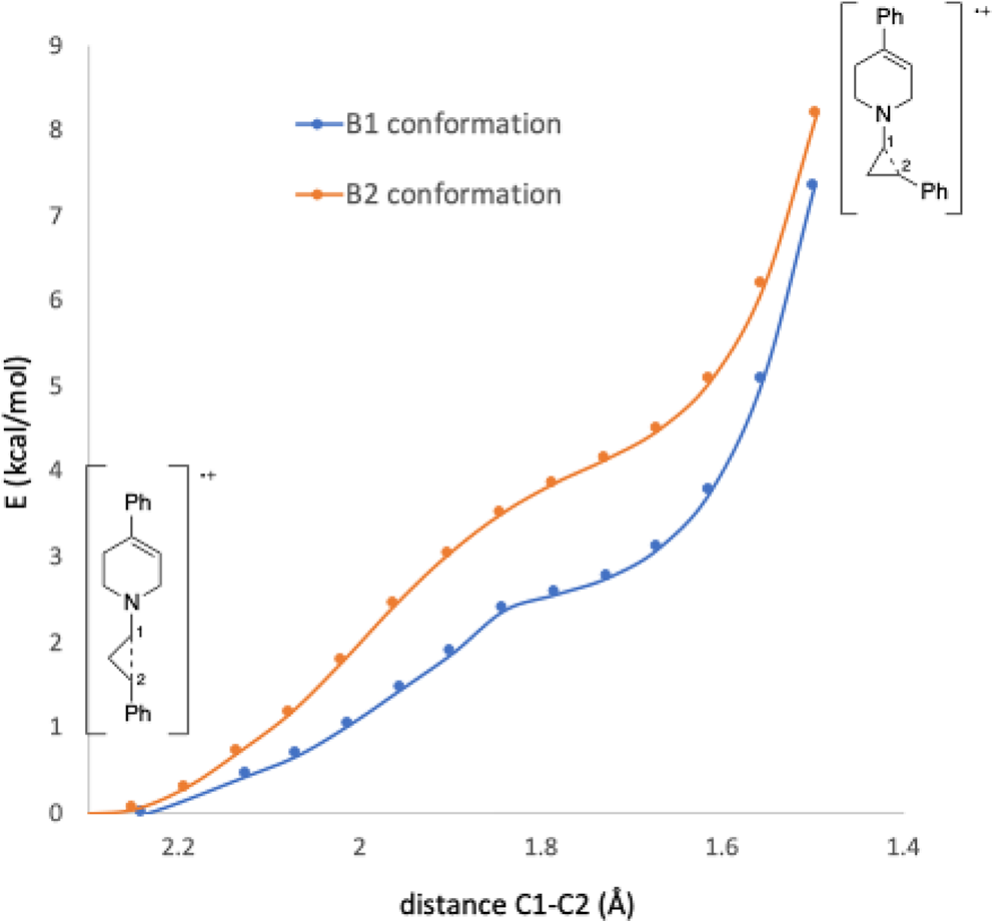
Energy profile (energy vs C1–C2 bond length) for **4c** ^•+^. Calculations were performed at the M06-2X/cc-pVDZ level.

As noted in the introduction, the use of *N*-cyclopropyl substrates as probes for single electron transfer is predicated on the assumption that, in analogy to the cyclopropylcarbinyl → homoallyl neutral radical rearrangement, ring opening is rapid and essentially irreversible. These calculations reveal that this may only hold true for **4c** ^•+^. For **4a** ^•+^, calculations indicate that ring opening may be slow and thermodynamically unfavorable. The calculations for **4b** ^•+^ are particularly noteworthy. For the *cis* isomer, there is a low barrier to ring opening, and the overall process is favorable. Ring opening of the *trans* isomer has a higher barrier and is unfavorable. More importantly, the ring-closed *trans* form of **4b** ^•+^ is *lower in energy* than either *cis*-**4b** ^•+^ (ring closed) or any of the ring opened structures. This prediction is supported by the experimental results, because if one looks carefully at the cyclopropyl region of the ^1^H NMR spectra before (*t* = 0) and after reaction (*t* = 20 h), it is clear that a significant amount of the starting material has undergone *cis* → *trans* isomerization during the course of the reaction (Figure 10). The results clearly demonstrate that while ring opening of *cis*-**4b** ^•+^ may be favorable, the lowest energy product that arises from that process is *trans*-**4b** ^•+^ and not the ring opened products.

**Figure 10:**
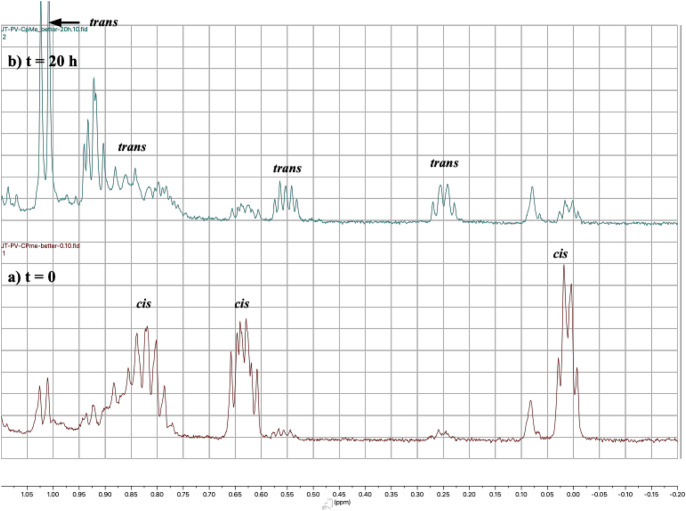
^1^H NMR spectra of the cyclopropyl region from the reaction of **3MLF** and *cis*-**4b** in CD_3_CN at (a) *t* = 0, and (b) *t* = 20 h. The labeled peaks show the position of the (clearly resolved) cyclopropyl hydrogens of the starting material, and demonstrate that *cis*/*trans* isomerization occurred during the course of the reaction. *Cis/trans* assignments were based upon ^1^H NMR spectra of the pure compounds (see Supporting Information).

Referencing Figure 3, the ratio of ring-opened to ring-closed products is expected to be directly proportional to the rate constant ratio *k*_*o*_/(*k*_*B*_[B:]). And, as implied in Table 3, the rate constants for ring opening are expected to vary greatly with **4c** ^•+^ predicted to be so short-lived, that only ring-opened products would be expected, i.e., no **5c** should have been produced! Overall, for the reaction of compounds **4**(**a**–**c**) with **3MLF**, the amount of ring opened products observed seems unrelated to rate of ring opening. We believe that the reason for this is that **5**(**a**–**c**) are *not* formed as a result of the competition depicted in Figure 3, but rather, as a consequence of the hydrogen atom abstraction from substrate described above as shown in Figure 11. The substrate radical generated from this process is readily oxidized under the reaction conditions, providing a pathway for the formation of a fully oxidized product with the cyclopropyl group intact because a radical is never generated in the vicinity of the cyclopropyl moiety.

**Figure 11:**
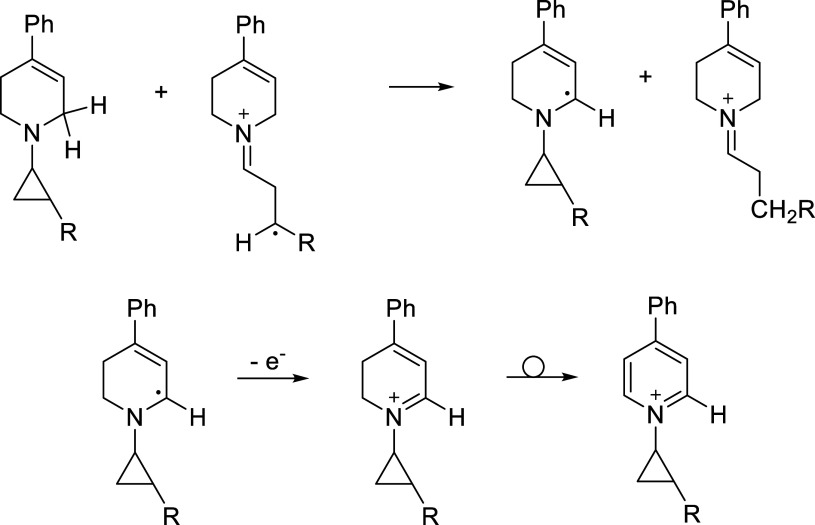
Substrate radical generated by hydrogen atom abstraction can undergo further oxidation leading to a pyridinium ion with the cyclopropyl group intact.

As noted earlier, when the anaerobic reactions of 1:1 **3MLF**:**4**(**a**–**c**) were analyzed by NMR, no peaks associated with **3MLF** were observed and there are a few unknown peaks (noted in the dotted rectangular boxes in Figure 6). In all these reactions, the major products were a cyclopropane ring closed pyridinium species (**5**), 4-phenylpyridine (**6**), low molecular weight aldehydes (**7** and **8**), and about 50% unreacted substrate (**4**). When the NMR tube was opened and exposed to oxygen, peaks attributable to **3MLF** reappeared while the unknown peaks vanished while some of the known peaks increased in intensity. The 1:1 ratio of reactants proved critical in revealing some of the nuances associated with these reactions, e.g., *cis*/*trans* isomerization of **4b**, which would have been missed if the reactions had gone to completion.

Consider the stoichiometry of these reactions. **3MLF** is a two electron oxidant. The oxidation of **4** to **5** (eq 1) is a four electron oxidation. The oxidation of **4** to **6** and **7** is also a four electron oxidation (eq 2). This of course means there is not enough **3MLF** to fully oxidize all of the substrate, which explains why there is only ca. 50% conversion. But, there must also be intermediate oxidation products formed from the substrate, specifically dihydropyridine (**DHP**) species that are precursors to **5** and **6**. We suspect that some of the unknown peaks in the NMR spectra of the reaction mixture that disappear upon exposure to oxygen may be these **DHP**s, which undergo autoxidation to **5** and **6** in the presence of O_2_. ^1^H NMR spectra for various of these **DHP**s have been reported,^34,35^ lending support to this idea.
1

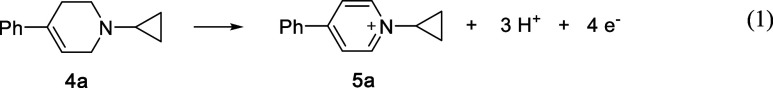



2

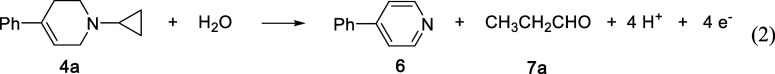




Finally, these reactions of **3MLF** and **4** (**a–c**) were also performed under aerobic conditions. The mass balance for **4a** was poor (because of overoxidation), but **4b** and **4c** provided useful new information. For **4b**, the results were essentially the same as observed anaerobically in terms of the production of **5** and **6**. The difference was that rather than butyraldehyde (**7b**), a different aldehyde was produced (acetaldehyde, in addition to **8b**). Similarly, for reaction of **4c**, benzaldehyde was produced in lieu of **7c**. These new products are clearly the result of trapping of the ring-opened distonic radical cations by O_2_, with subsequent decomposition of the resulting peroxides via classical free radical pathways (Figure 12). For all these aerobic reactions, **3MLF** was continuously regenerated by O_2_ and catalytic in the production of the various aldehydes (pertinent spectra are provided in the Supporting Information).

**Figure 12:**
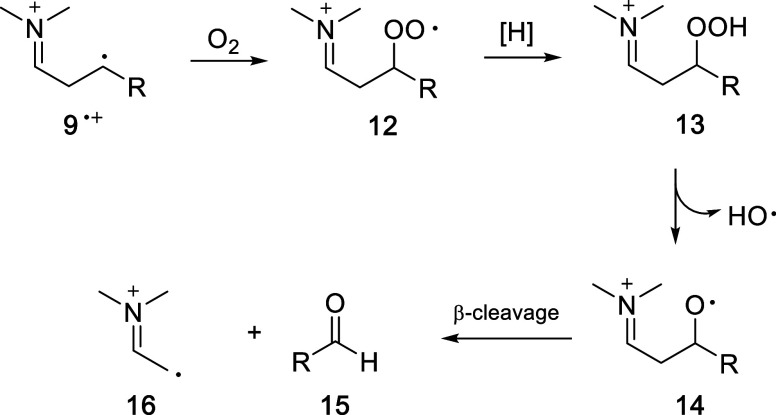
Trapping of the ring-opened, distonic radical cation **9** ^•+^ (R = CH_3_ and Ph) by molecular oxygen provides entry into the formation of additional low molecular weight aldehydes and other reactive species. The fate of **16** is unknown.

## Conclusions

As stated at the outset, the objective of this work was to examine the chemistry of 3° *N*-cyclopropyl compounds related to **MPTP** in a biomimetic system (**3MLF**/hν) in order to better understand how these SET probes might behave in MAO-catalyzed oxidations if electron transfer were to occur. Obviously, there are distinct differences between a biomimetic study such as this, and what may actually be occurring in the enzyme. The most striking difference in this case is that with **3MLF**, the reactions with the *N*-cyclopropyl-**MPTP** derivatives require light. As noted earlier though, the role of light is to modulate the electrochemical potential of **3MLF**; the excited state is a more potent oxidant. Regardless of whether SET occurs thermally or photochemically, the products are the same–formation of an amine radical cation and a flavin radical anion. These experiments focus on the chemistry that occurs after single electron transfer, and it is *that* chemistry which may be relevant to MAO. This work has shown that many of the anomalies reported in the literature regarding the reaction mechanism of MAO catalyzed oxidations and possible role of SET can be understood by considering the electronic properties of the amine, stability of radicals produced, the rate of cyclopropyl ring opening of radical cation intermediates relative to other competing processes, and reactive products arising from rupture of the cyclopropane moiety. The implications arising from these results are

1. A possible reason that **MPTP** and related compounds are the only known tertiary amines with MAO substrate or inhibitor properties may be that the α-C=C in these compounds dramatically lowers the p*K*_a_ of the corresponding aminyl radical cations (compared to fully aliphatic tertiary amines). The combined resonance effect of the α-C=C and α-nitrogen in these carbon-centered radicals leads to an exceptionally stable free radical.
2. The use of *N*-cyclopropyl compounds as SET probes assumed that, like the cyclopropyl-carbinyl radical rearrangement, ring opening would be rapid and irreversible. For **4a** ^•+^ and **4b** ^•+^ this is certainly not the case. Nonetheless, even though ring opening is unfavorable and reversible, the SET pathway can lead to detectable products.
3. One electron oxidation of **4c** likely proceeds via a concerted dissociative electron transfer process; **4c** ^•+^ does not likely exist at a potential energy minimum.
4. One of the suggested mechanisms for MAO inactivation by *N*-cyclopropyl compounds involved coupling of the ring opened distonic radical cation with a flavin radical (also formed by SET) in the active site. This biomimetic study found no evidence for coupling with **3MLF** derived radicals either by ^1^H NMR or LC-MS/MS.
5. Cyclopropane ring opening of **4**(**a**–**c**)^•+^ results in formation of an iminium species, which upon hydrolysis, leads to low molecular weight aldehydes. It is feasible that formation of such aldehydes in the active site provide another pathway that leads to MAO inactivation. In essence, in addition to generating a reactive radical which disrupts the active site, these *N*-cyclopropyl compounds may act by delivering a highly reactive electrophilic species as a possible alternative mechanism for inhibition.
6. The radical portion of the ring-opened, distonic radical cation is reactive and, as demonstrated herein, can abstract hydrogen from a C–H bond (substrate). In the active site of MAO, there are certainly residues present that would be more effective hydrogen atom donors leading to another potential mechanism for enzyme inactivation. A plausible hydrogen atom source is the phenolic functionality of tyrosine, a key amino acid involved in MAO catalysis.^36^ (Tyrosyl radicals have been detected in MAO catalysis.)^17^
7. Similarly, the radical portion of this radical cation can be trapped by oxygen leading to other reactive species. To the extent there is dissolved oxygen in the active site of MAO, such trapping may lead to peroxide formation, hydroxyl radical, and low molecular weight aldehydes whose chemistry might disrupt the active site.

## Experimental Section

All chemicals and solvents used were at the highest purity available. Details of the synthesis of 3-methyllumiflavin (**3MLF**) can be found in the work originally reported by Ghisla et al.^37^ All **MPTP** derivatives were synthesized according to literature procedures and stored as their respective oxalate or hydrochloride salts: **4a**,^38^ *cis*-**4b**,^32^ *trans*-**4c**,^32^ and **4a**_**D4**_.^39^ The salts were treated using aqueous potassium carbonate solutions and the generated free amine was extracted using methylene chloride.

Caution: **MPTP** is a known nigrostriatal neurotoxin and should be handled with care under a ventilated hood and with the proper personal protective equipment. Procedures for the safe handling of **MPTP** have been documented.^40^ Although the compounds chosen for study have no known human toxicity, these precautions were followed nonetheless.

NMR spectra were recorded using a Bruker Neo400HT NMR (400 MHz). Data analyses were performed using MestreNova. All reactions were prepared using degassed CD_3_CN stock solutions. Stocks were kept refrigerated unless being actively used. This was done primarily to maintain consistency in sample preparation. Aerobic reactions were prepared directly in NMR tubes with a total reaction volume of approximately 0.6 mL. To ensure proper and consistent aeration of the sample, the tubes were placed on a mixing table when not being analyzed. Anaerobic reactions were also prepared directly in NMR tubes, but were immediately freeze–pump–thaw degassed (the process started <1 min after mixing). After degassing, the NMR tubes were flame-sealed. The reaction mixtures were irradiated with a 50W tungsten lamp from a distance of ca. 1–2 feet.

Molecular orbital calculations at the M06-2X^33^ and CAM3-B3LYP^41^ levels were performed using either Gaussian 09 ^42^ (Table 1) or Spartan’24 ^43^ (Table 3 and Figure 7) to support and amplify these findings. M06-2X/def2-TZVP has been shown to be effective in terms of the interplay between accuracy and computational efficiency in calculations involving radicals.^44^ CAM3-B3LYP performs well when long-range interactions are important, such as might be the case for the ring-opened distonic radical cations **4­(a–c)** ^•+^.^41^ All geometry optimizations were performed using the functional and basis sets indicated in the results. The optimized geometries were verified as transition state structures (one imaginary frequency corresponding to the reaction coordinate) or minima (zero imaginary frequencies) by frequency calculations.

## Supplementary Material



## Data Availability

The data underlying this study are available in the published article and its Supporting Information.

## Notes

Spectral data for the reaction of **4**(**a**–**c**), **4a**_**D4**_ under anaerobic and aerobic conditions, authentic spectra of compounds pertinent to this study, details of the ^1^H NMR simulation shown in Figure 4, and the XYZ coordinates for all the optimized structures pertinent to Tables 1 and 3 (PDF)
