# Supplementary material for: A Biomimetic Study of the Behavior of N‑Cyclopropyl-Based Single Electron Transfer Probes in the Context of Monoamine Oxidase-Catalyzed Oxidations
Source: J Org Chem. 2026 Jan 10;91(3):1371–82. doi: 10.1021/acs.joc.5c02528 (PMC12836322; doi:10.1021/acs.joc.5c02528)
Supplement: Supplementary file 1 [file jo5c02528_si_001.pdf]

Supporting Information for:

A Biomimetic Study of the Behavior of *N*-Cyclopropyl-Based Single Electron Transfer Probes in the Context of Monoamine Oxidase Catalyzed Oxidations

*Nathan Price, Bradley Engels, Paul Venturo, Jonathan Sánchez González, Thomas Robbins, Joseph Barton, Neal Castagnoli, and James M. Tanko**

Department of Chemistry, Virginia Polytechnic Institute and State University, Blacksburg, VA 24060, USA

email: jtanko@vt.edu

Contents

Spectral data for the reaction (anaerobic) of **4a** with 3MLF	S2
Spectral data for the reaction (anaerobic) of *cis*-**4b** with 3MLF	S8
Spectral data for the reaction (anaerobic) of *trans*-**4c** with 3MLF	S14
Spectral data for the reaction (anaerobic) of $\mathbf{4a_{D4}}$ with 3MLF	S20
Spectral data for the reaction (aerobic) of *cis*-**4b** with 3MLF	S26
Spectral data for the reaction (aerobic) of *trans*-**4c** with 3MLF	S29
Authentic 400 MHz ^{1}H NMR spectra of compounds pertinent to this study	S32
Details of the ^{1}H NMR simulation shown in Figure 8 of the manuscript	S42
Computed energies used for the results summarized in Table 1	S43
XYZ coordinates for optimized structures pertinent to Table 1	S44
Results obtained from molecular orbital calculations pertaining to the barrier ($\Delta E^{\neq}$) and energetics (ΔE^{o}) of ring opening for $\mathbf{4(a - c)^{\bullet +}}$	S49
XYZ coordinates for optimized structures pertinent to Table 3	S52
Reviewer comments (not directly addressed in the manuscript)	S69

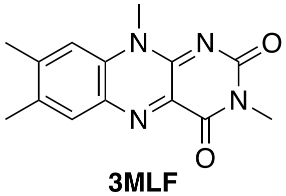

3MLF

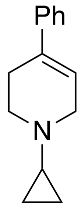

4a

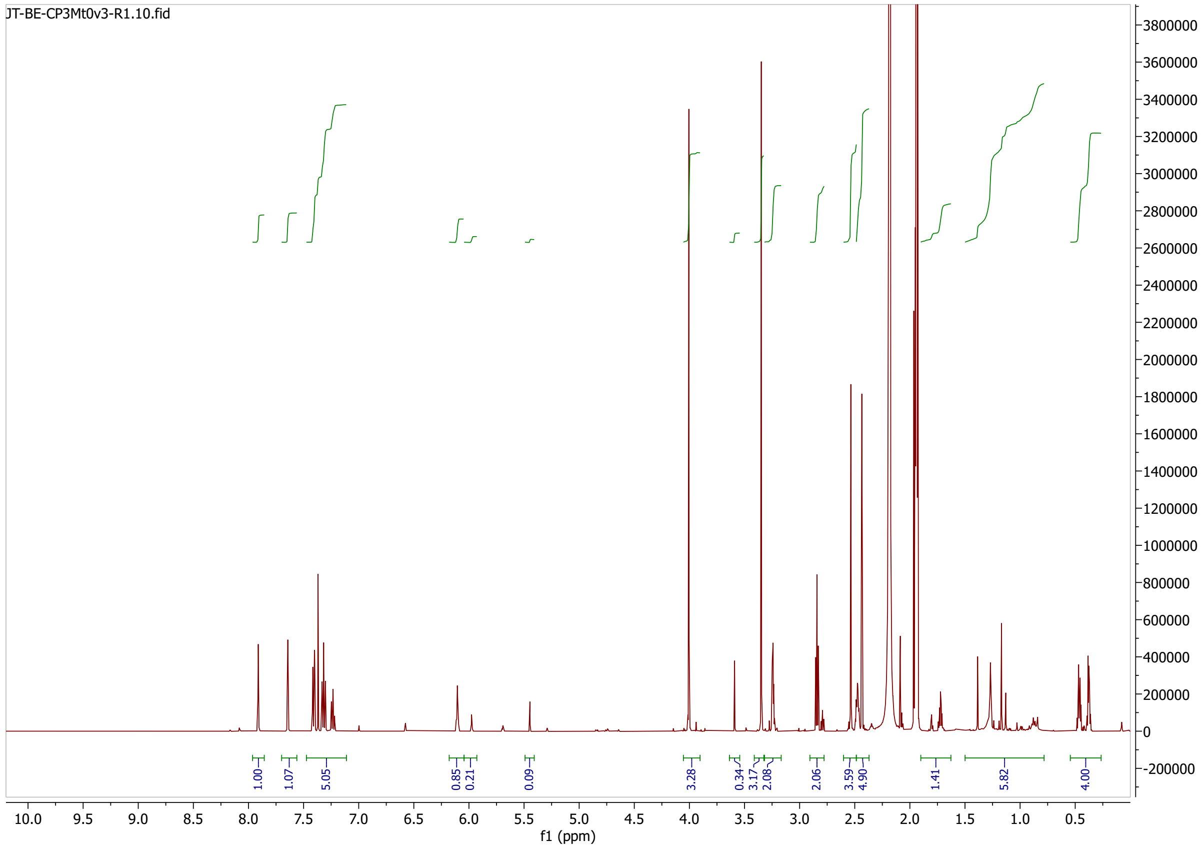

Figure S1. 400 MHz ^{1}H NMR spectrum for the reaction (anaerobic) of **4a** with **3MLF** (t = 0) in CD_3CN.

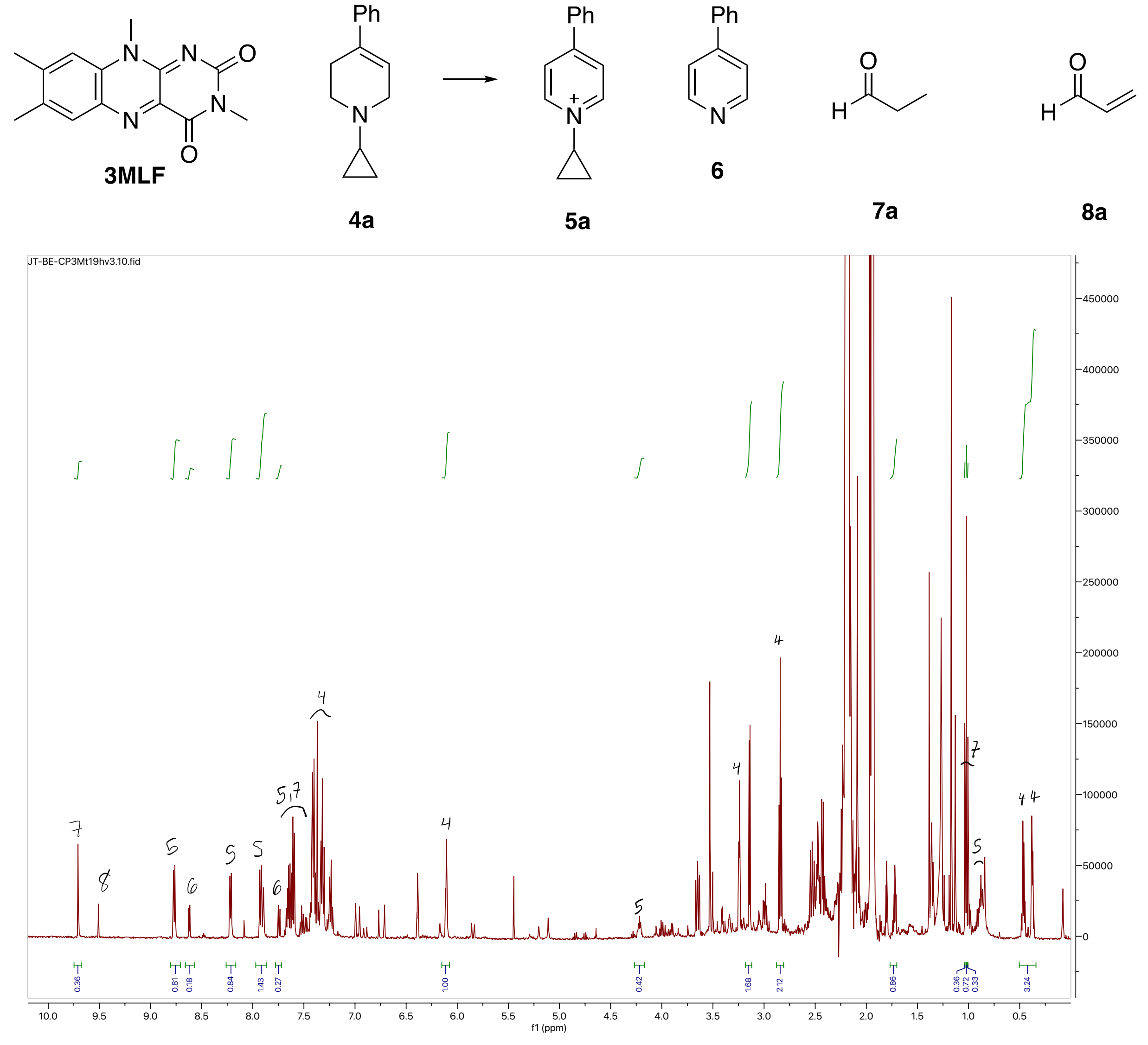

Figure S2. 400 MHz ^{1}H NMR spectrum for the reaction (anaerobic) of **4a** with **3MLF** (t = 19 h) in CD_3CN.

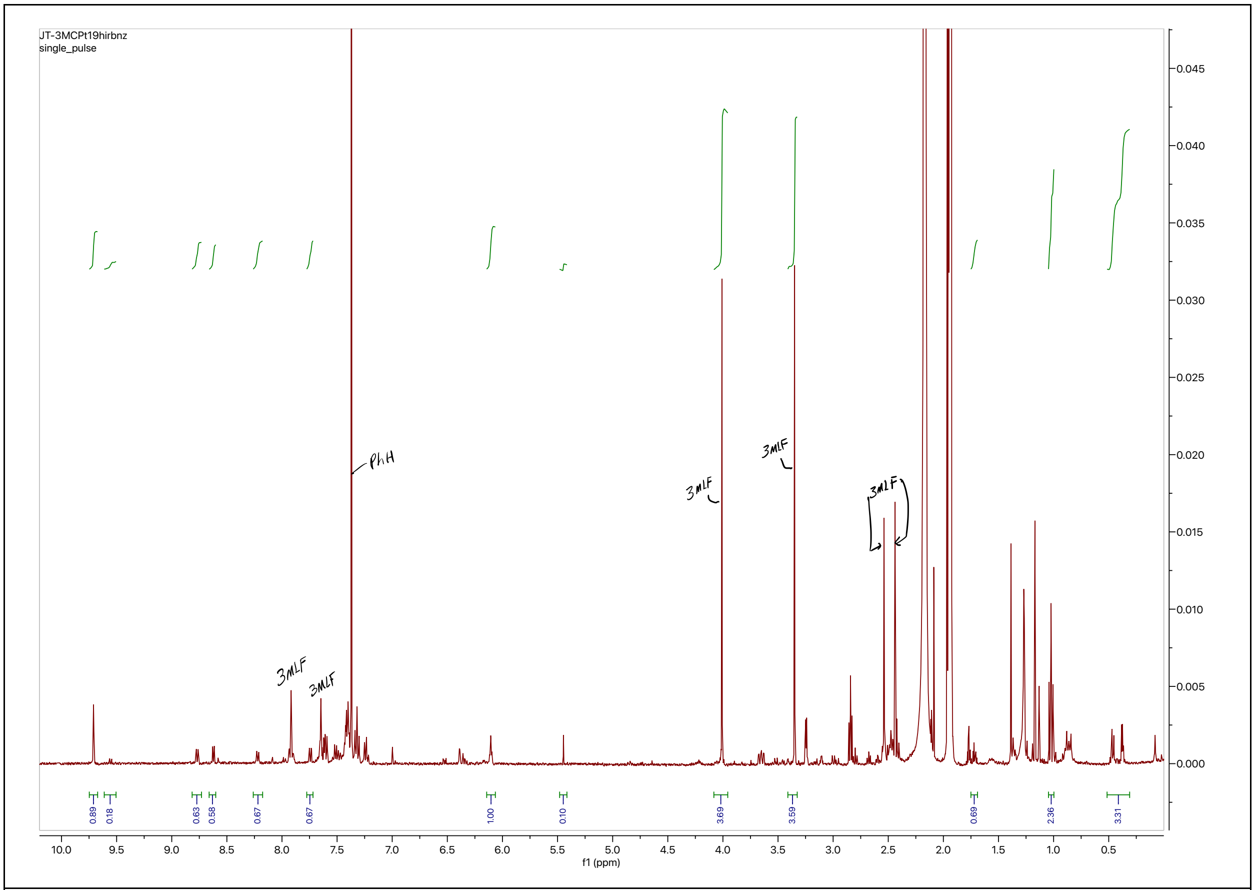

Figure S3. 400 MHz ^1H NMR spectrum for the reaction (anaerobic) of **4a** with **3MLF** (t = 19 h and after exposure to O_2) in CD_3CN.

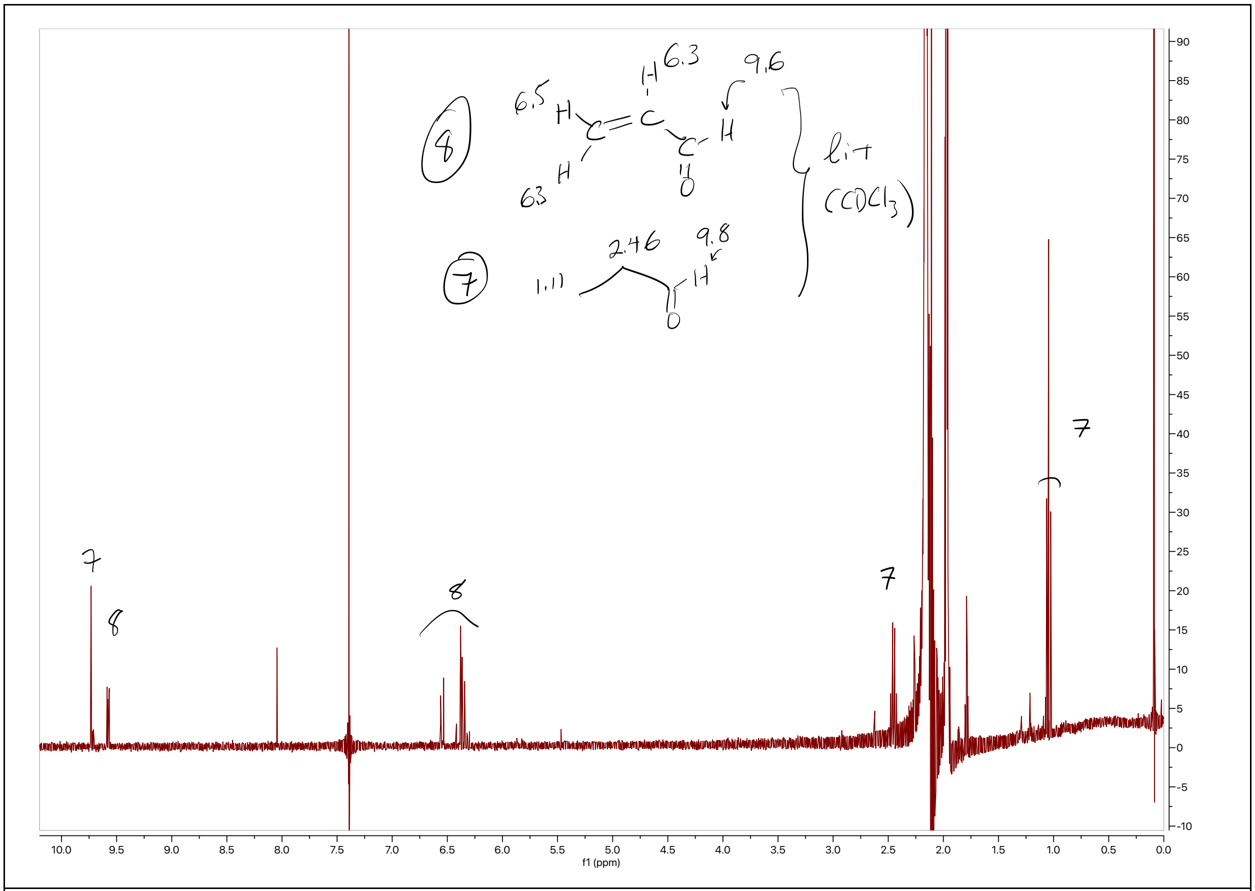

Figure S4. 400 MHz ^{1}H NMR spectrum for the distillate obtained from the reaction (anaerobic) of **4a** with **3MLF** in CD_3CN.

a)

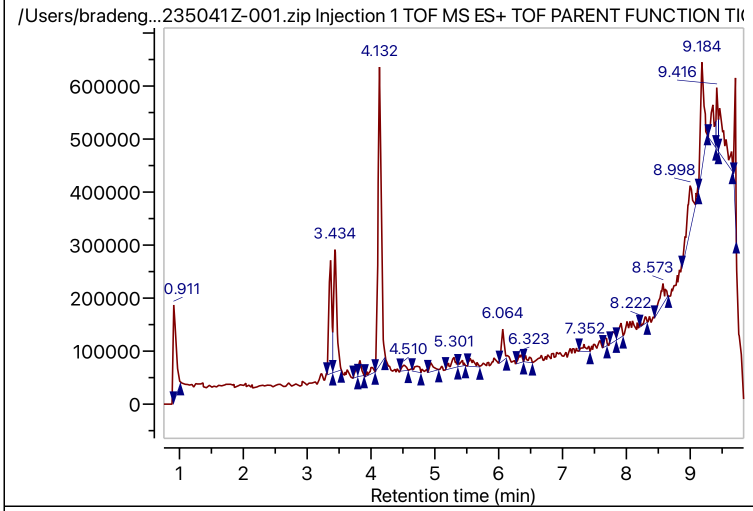

b) 3.36 min

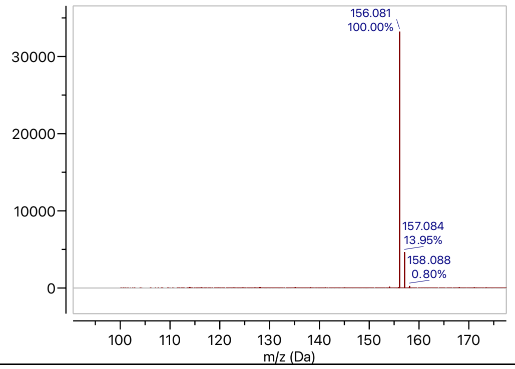

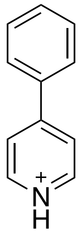

Exact Mass: 156.081

c) 3.43 min

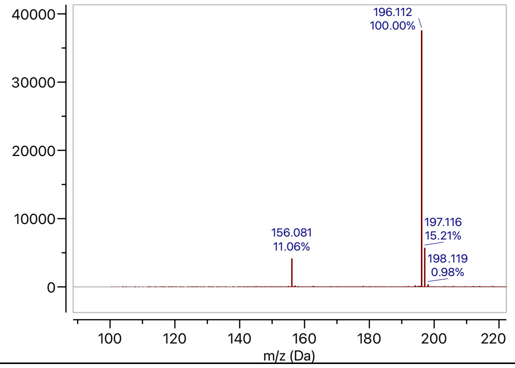

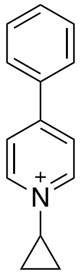

Exact Mass: 196.112

d) 3.83 min

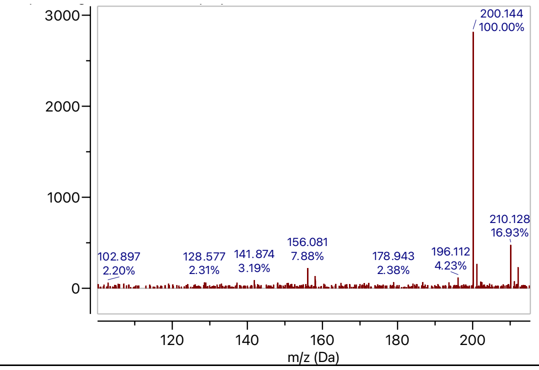

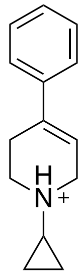

Exact Mass: 200.143

e) 4.13 min

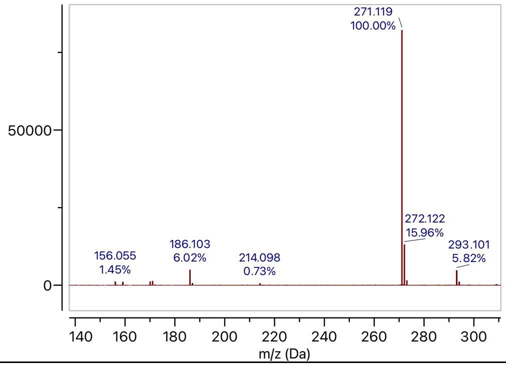

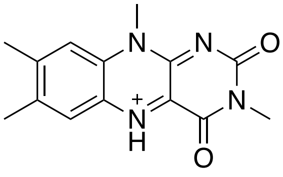

Exact Mass: 271.119

Figure S5. LC-MS/MS results for the reaction (anaerobic) of **4a** with **3MLF**.

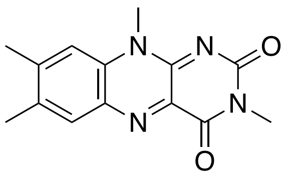

3MLF

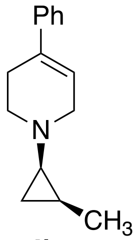

4b

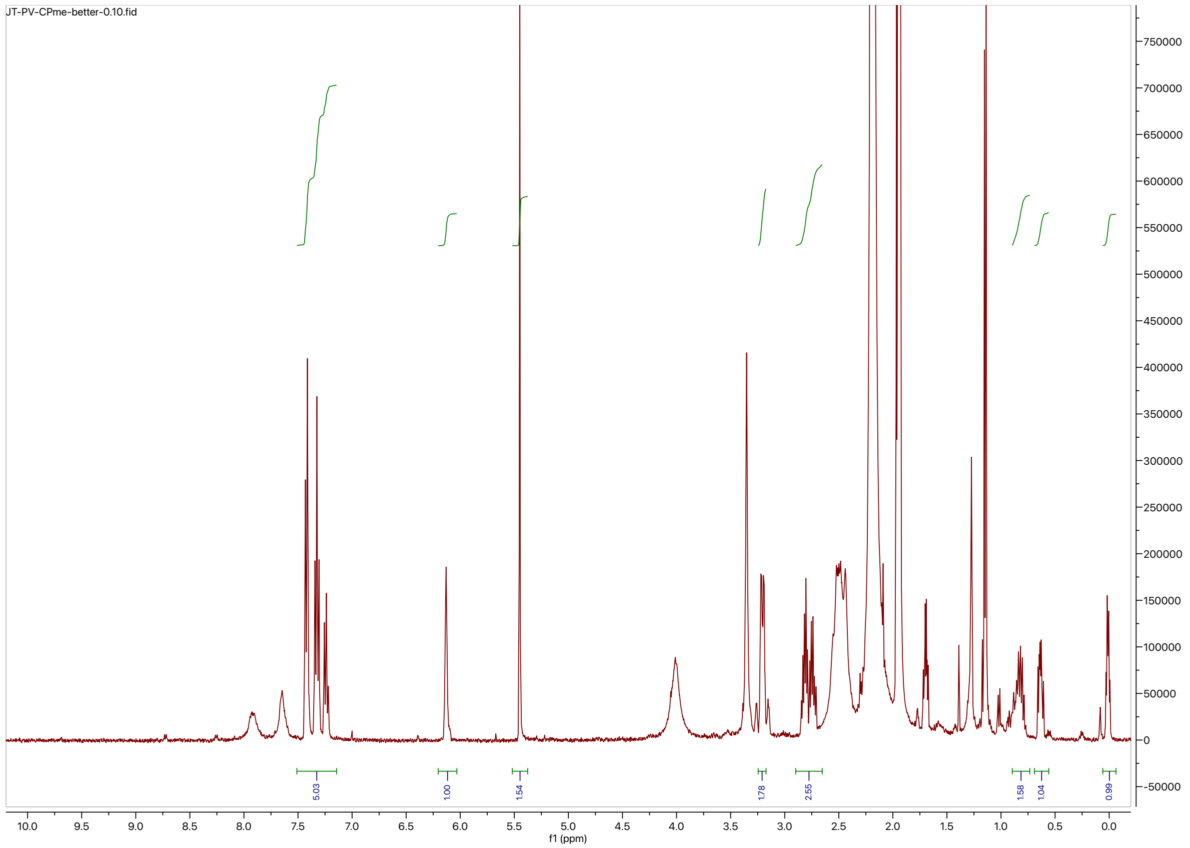

Figure S6. 400 MHz ^{1}H NMR spectrum for the reaction (anaerobic) of *cis*-**4b** with **3MLF** (t = 0) in CD_3CN.

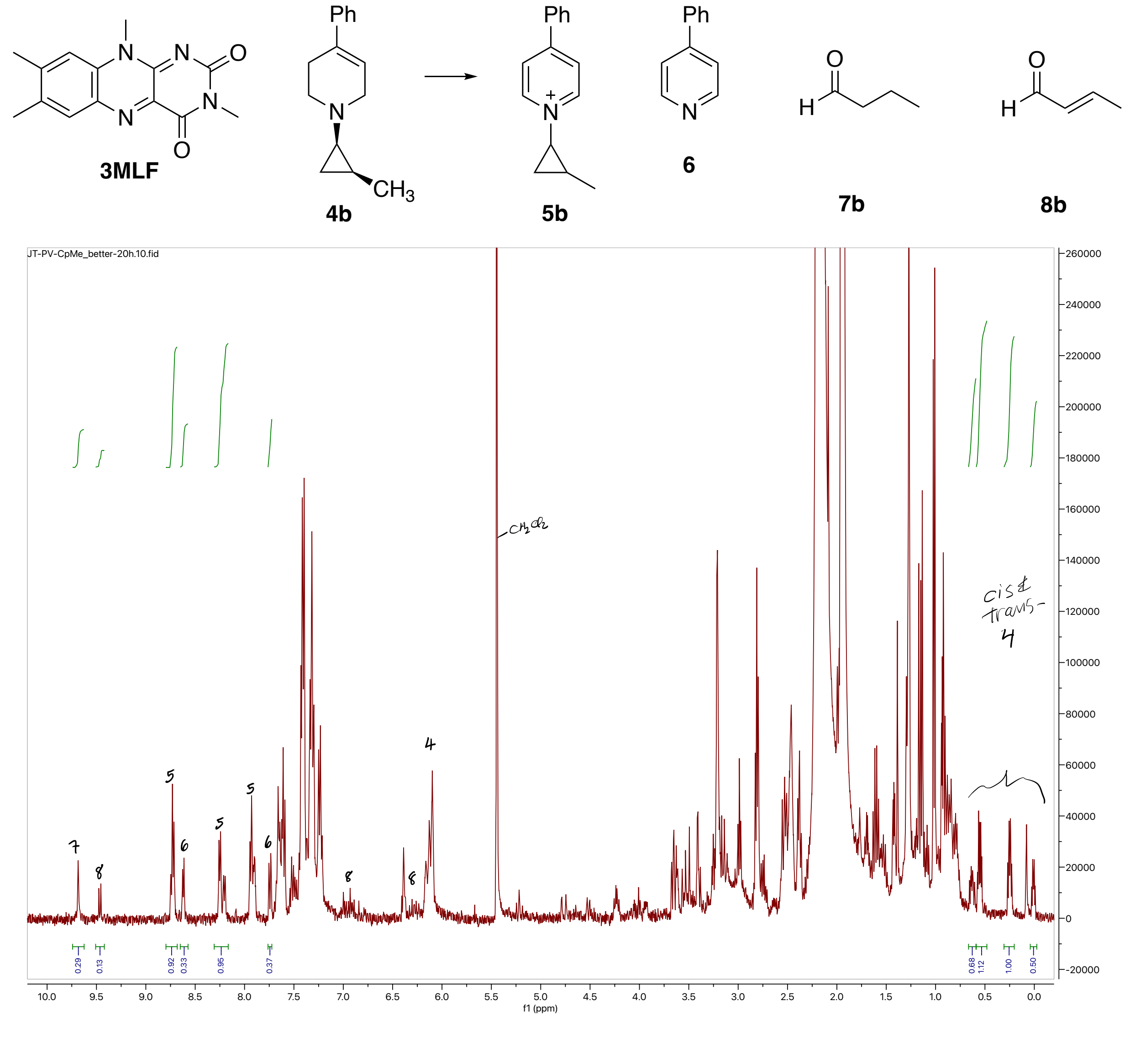

Figure S7. 400 MHz ^{1}H NMR spectrum for the reaction (anaerobic) of *cis*-**4b** with **3MLF** (t = 20 h) in CD_3CN.

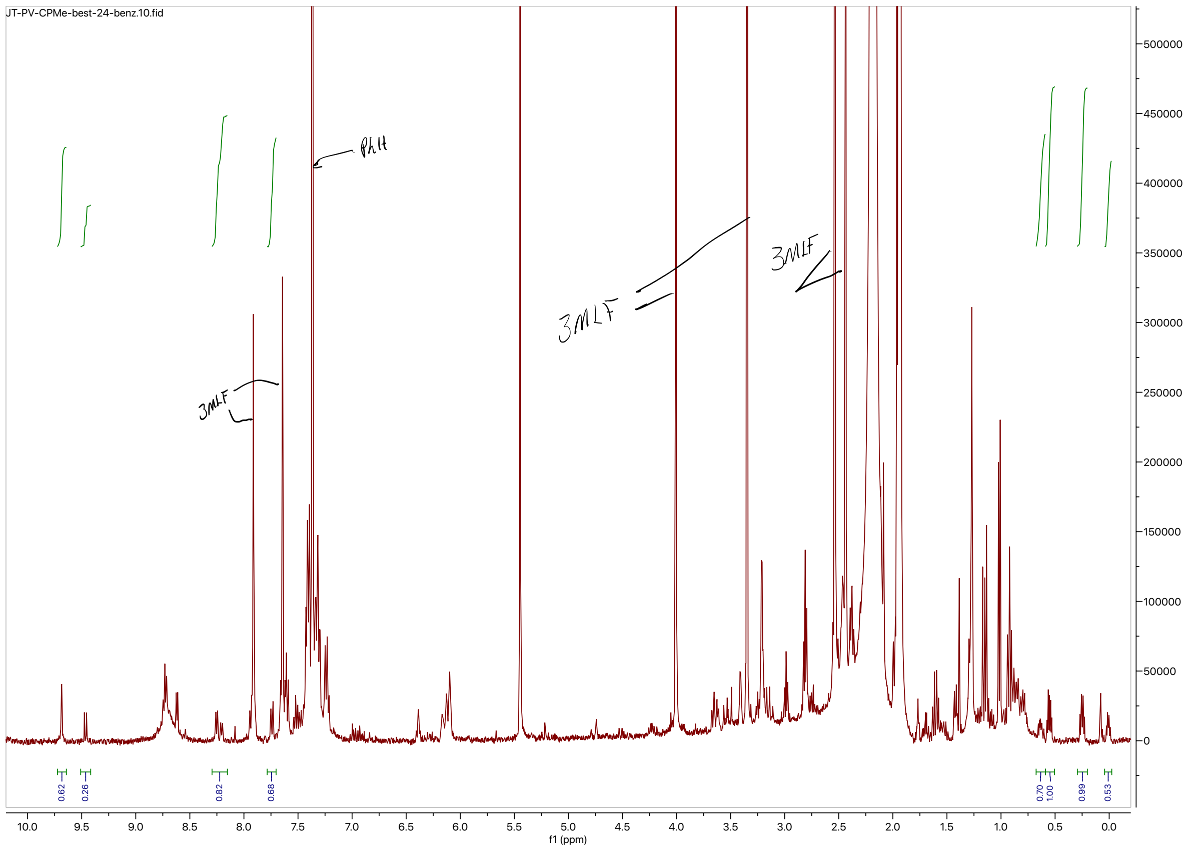

Figure S8. 400 MHz ^{1}H NMR spectrum for the reaction (anaerobic) of *cis*-**4b** with **3MLF** (t = 24 h and after exposure to O_2) in CD_3CN.

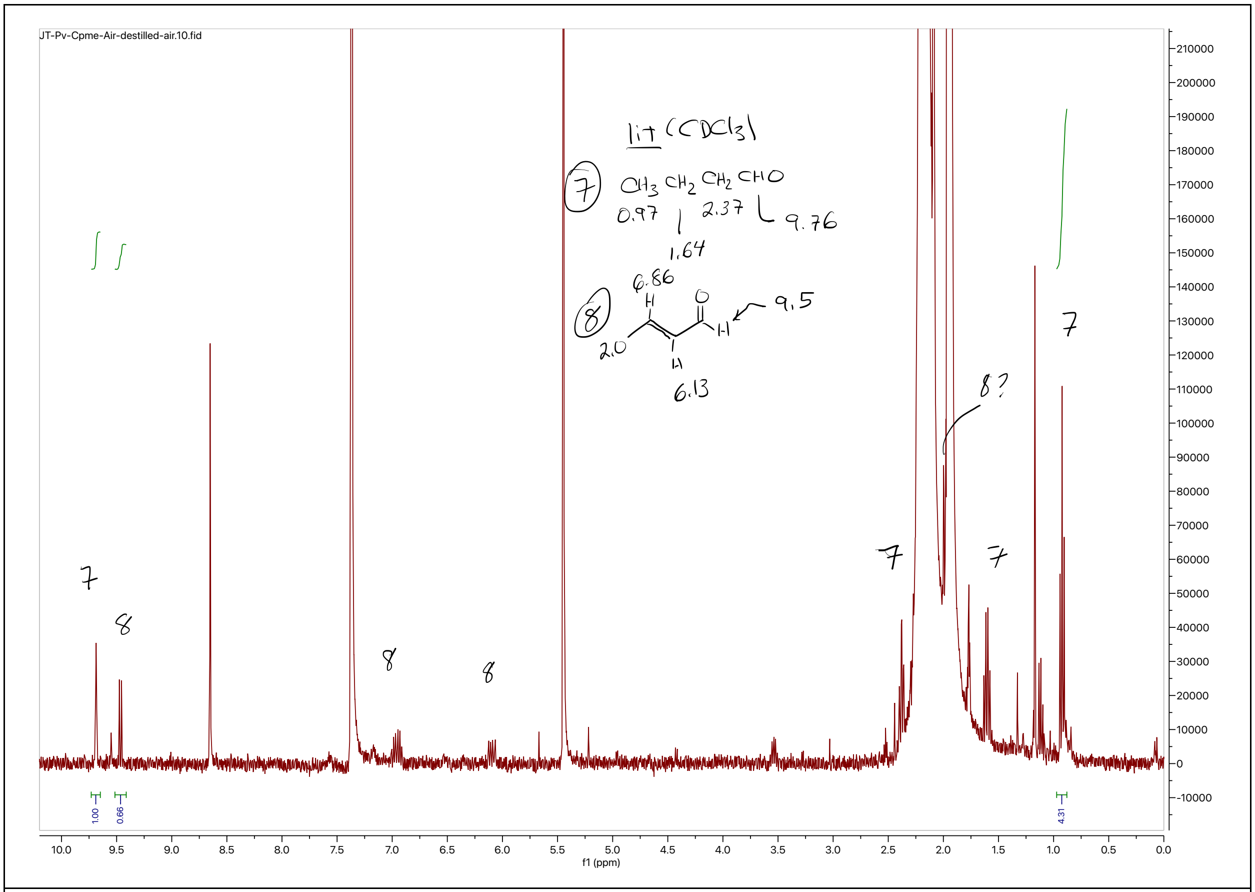

Figure S9. 400 MHz ^{1}H NMR spectrum for the distillate obtained from the reaction (anaerobic) of *cis*-**4b** with **3MLF** in CD_3CN.

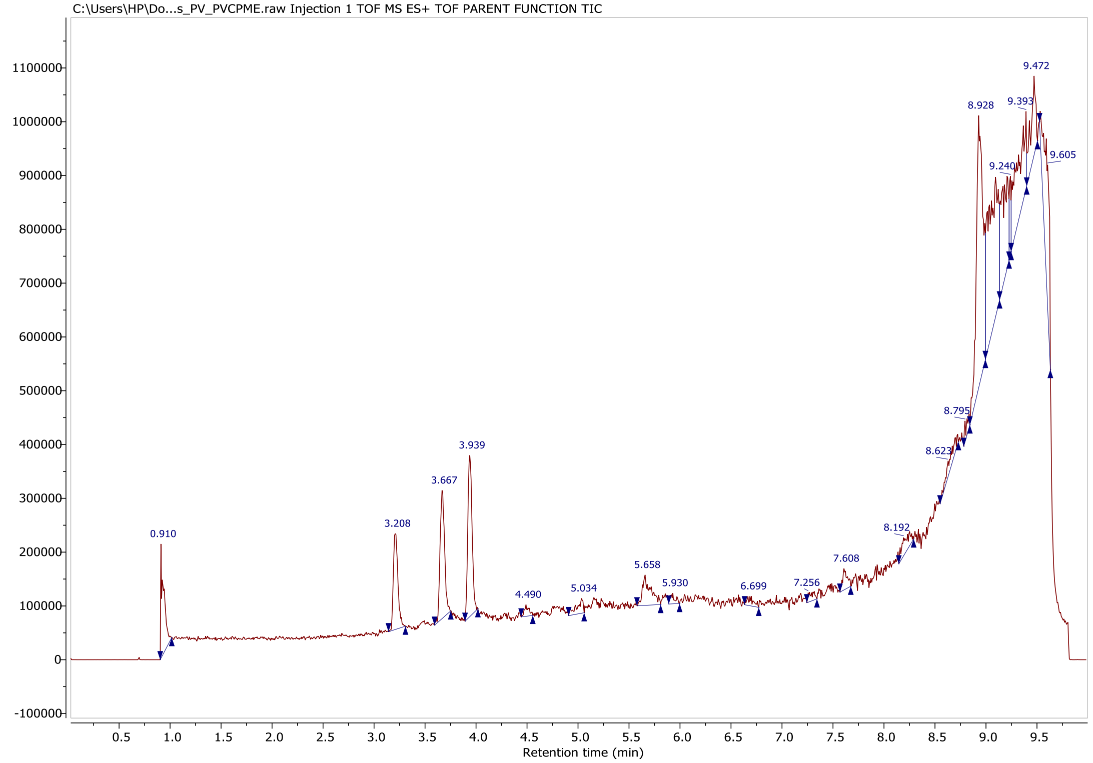

a)

b) 3.208 min

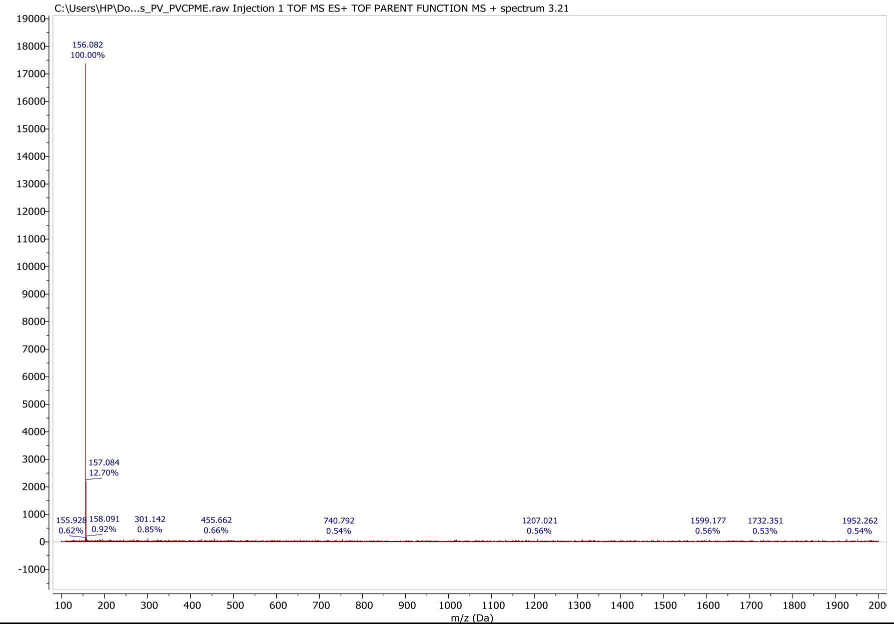

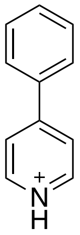

Exact Mass: 156.081

c) 3.667 min

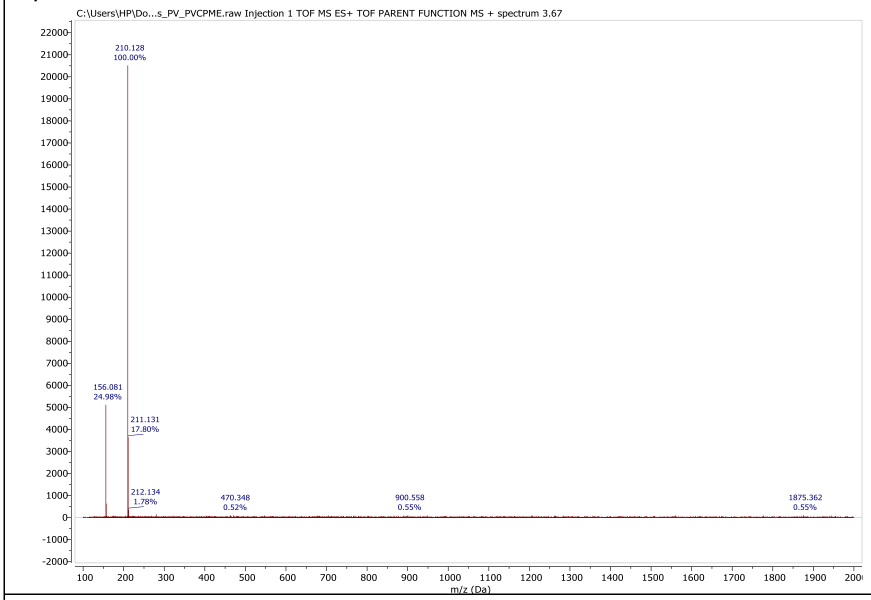

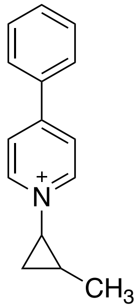

Exact Mass: 210.128

d) 3.939 min

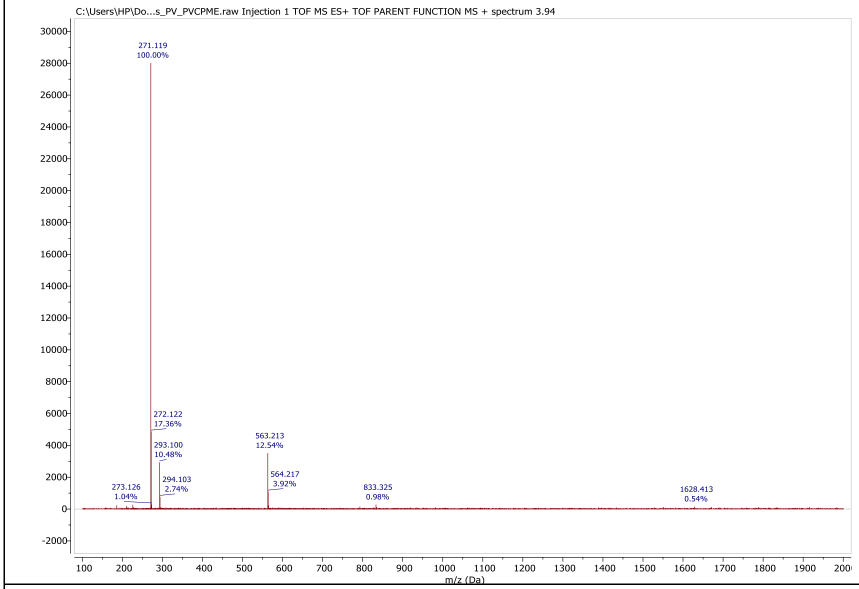

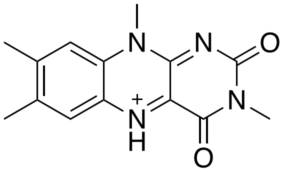

Exact Mass: 271.119

Figure S10. LC-MS/MS results for the reaction (anaerobic) of *cis*-**4b** with **3MLF**.

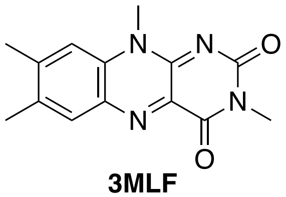

3MLF

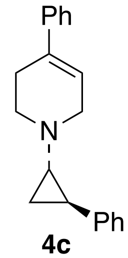

4c

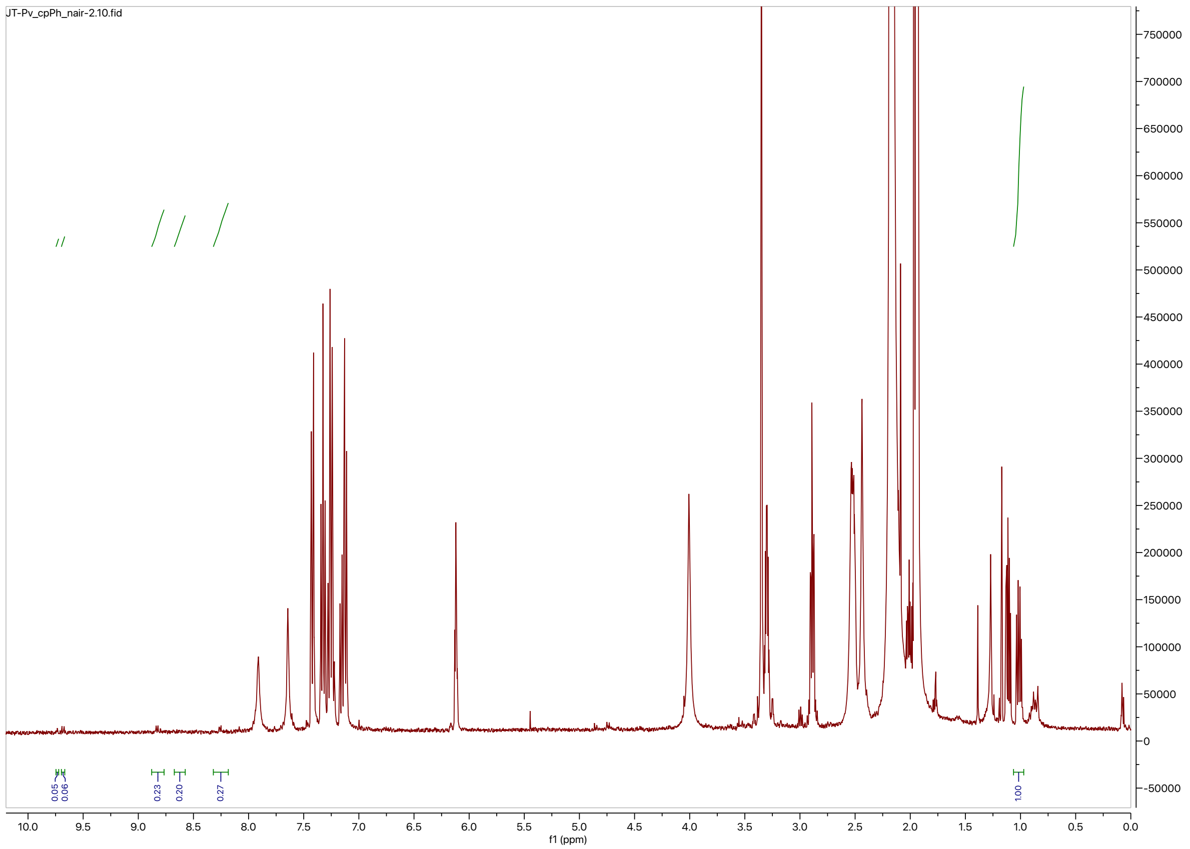

Figure S11. 400 MHz ^{1}H NMR spectrum for the reaction (anaerobic) of *trans*-**4c** with **3MLF** (t = 0) in CD_3CN.

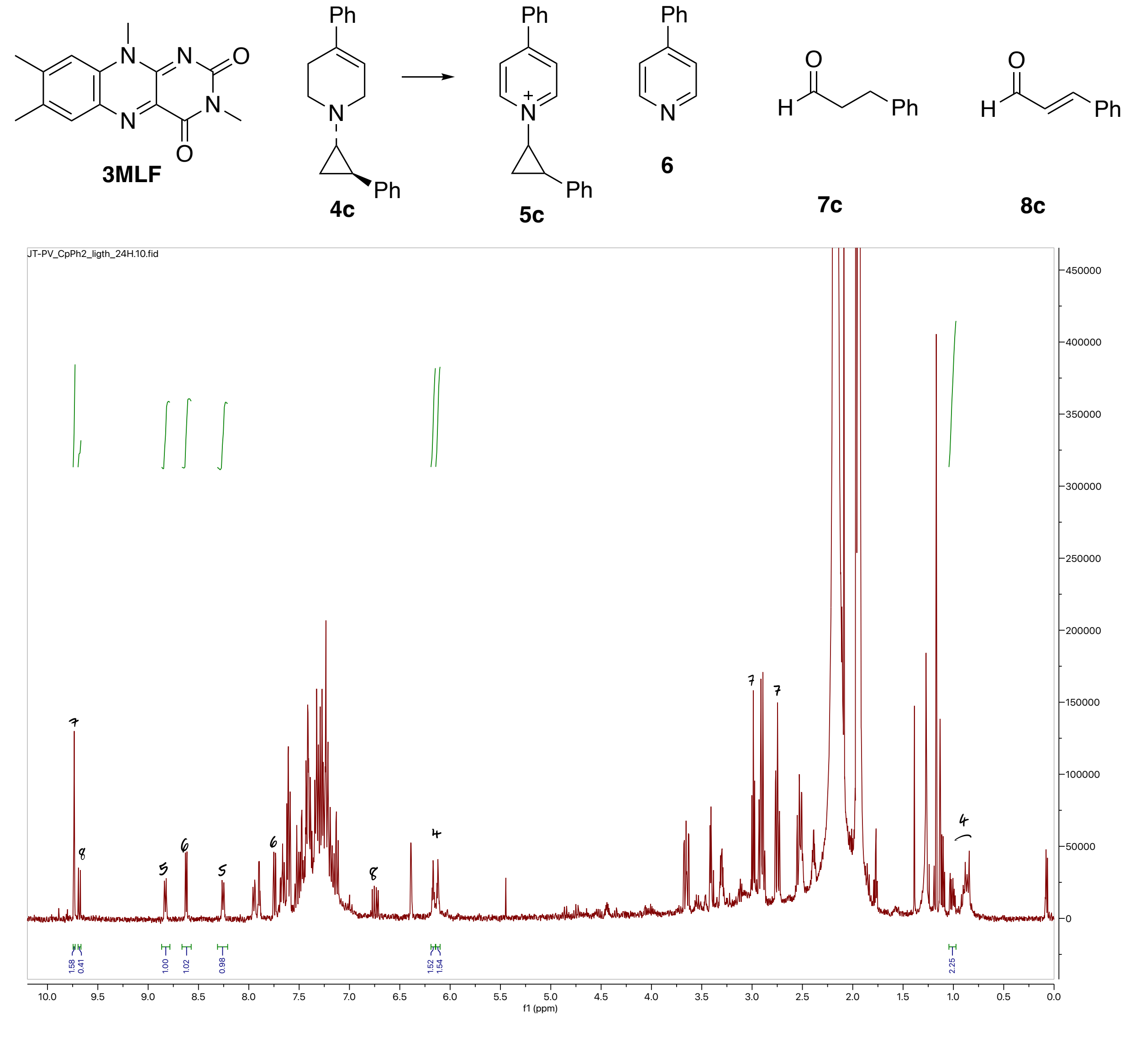

Figure S12. 400 MHz ^{1}H NMR spectrum for the reaction (anaerobic) of *trans*-**4c** with **3MLF** (t = 24h) in CD_3CN.

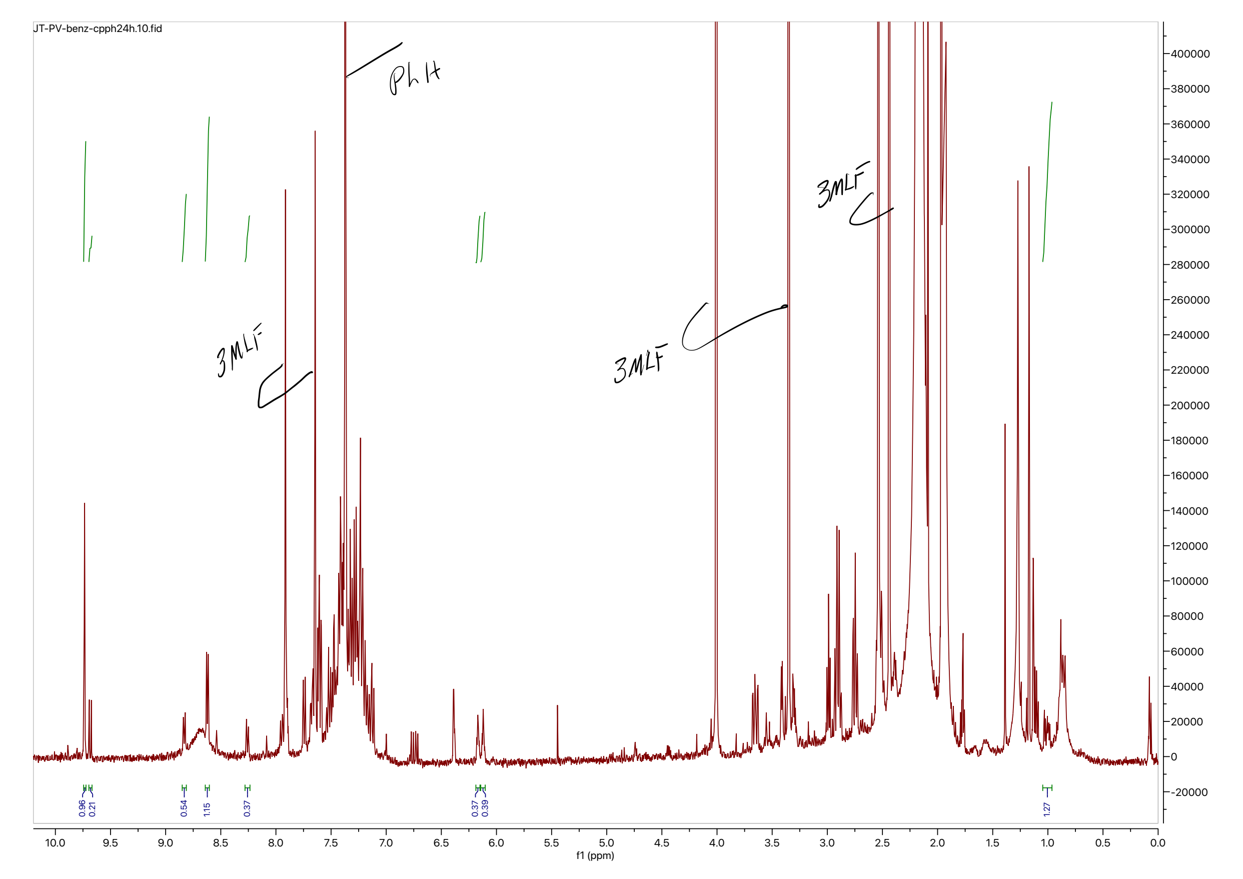

Figure S13. 400 MHz ^{1}H NMR spectrum for the reaction (anaerobic) of *trans*-**4c** with **3MLF** (t = 24 h and after exposure to O_2) in CD_3CN.

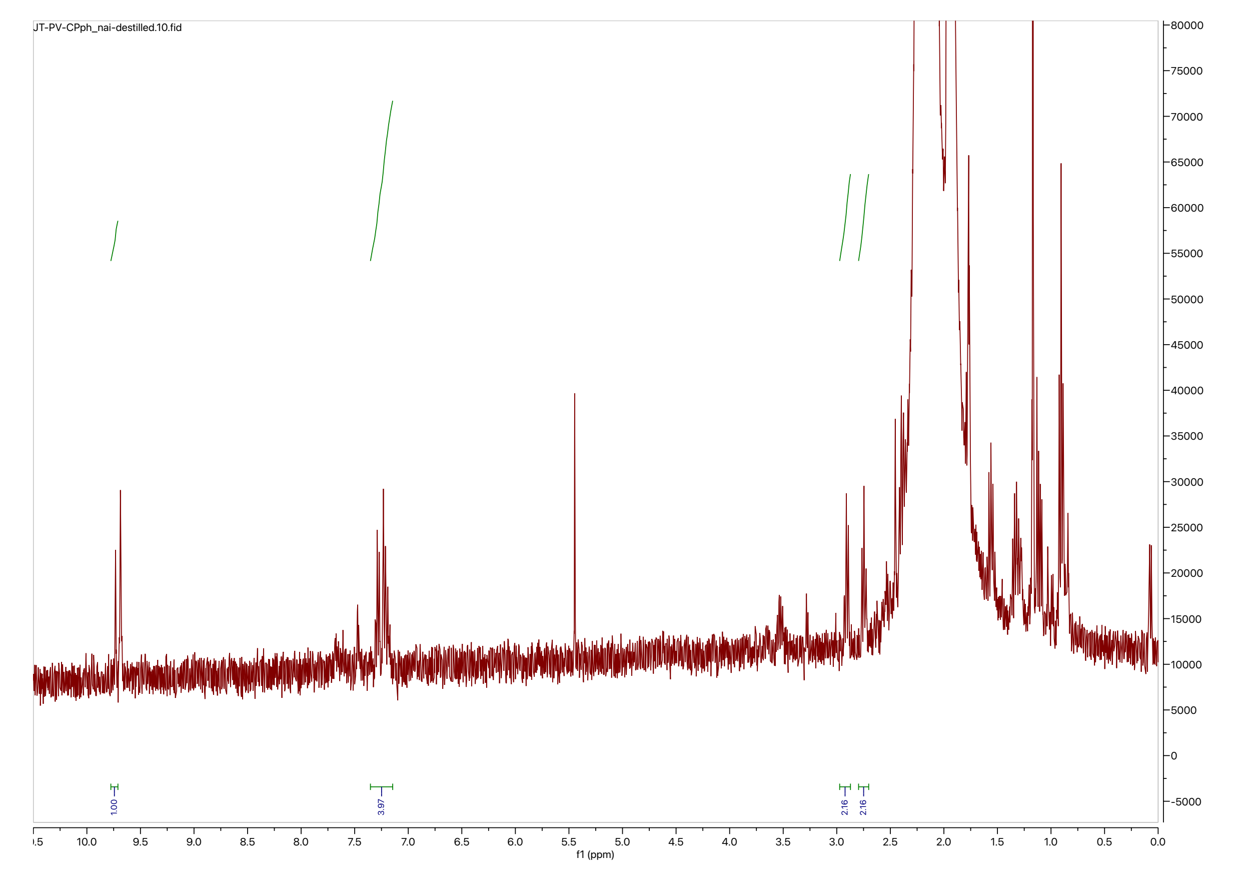

Figure S14. 400 MHz ^{1}H NMR spectrum for the reaction (anaerobic) of *trans*-**4c** with **3MLF** (distillate) in CD_3CN.

a)

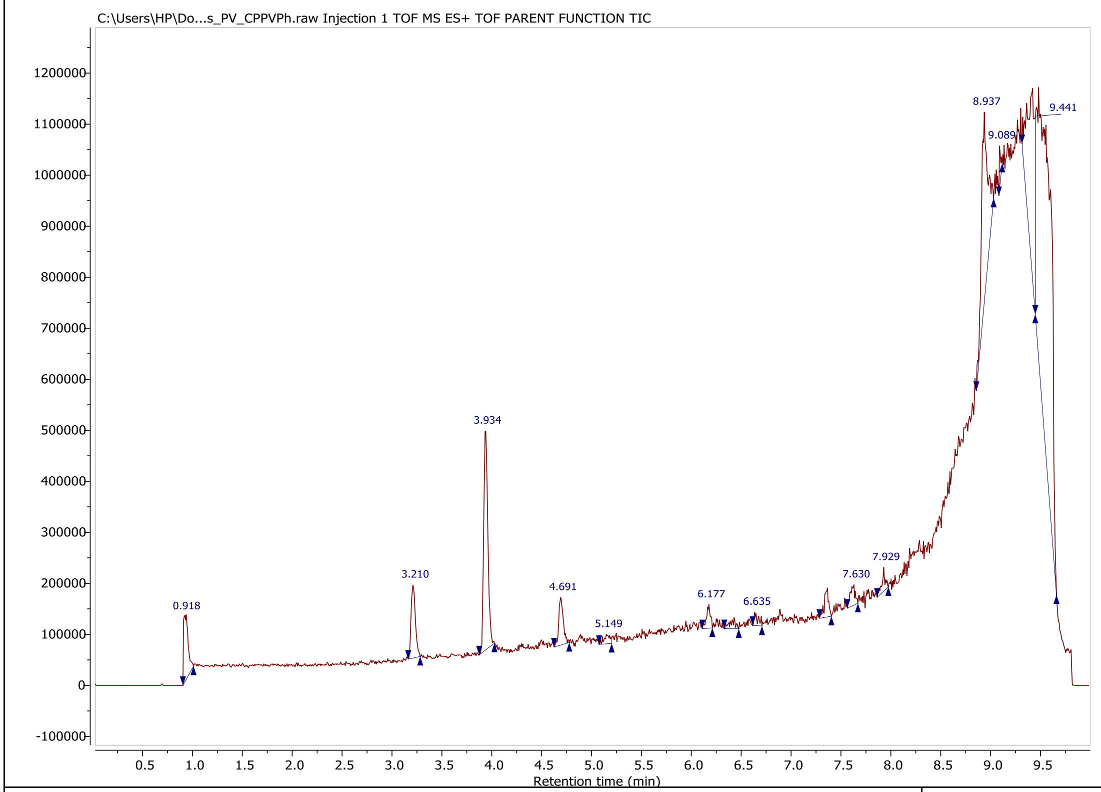

b) 3.21 min

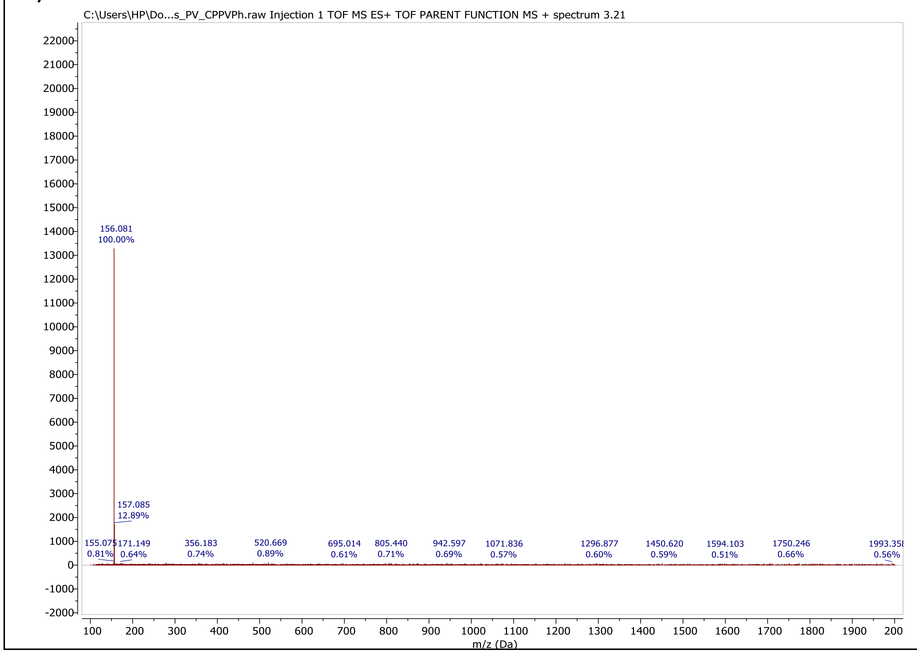

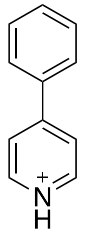

Exact Mass: 156.081

c) 3.934 min

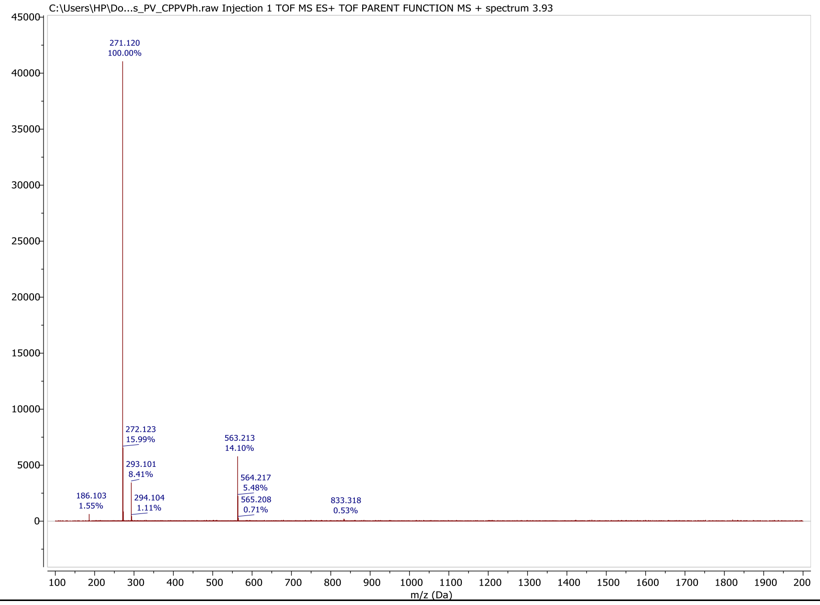

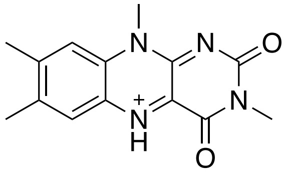

Exact Mass: 271.119

d) 4.691 min

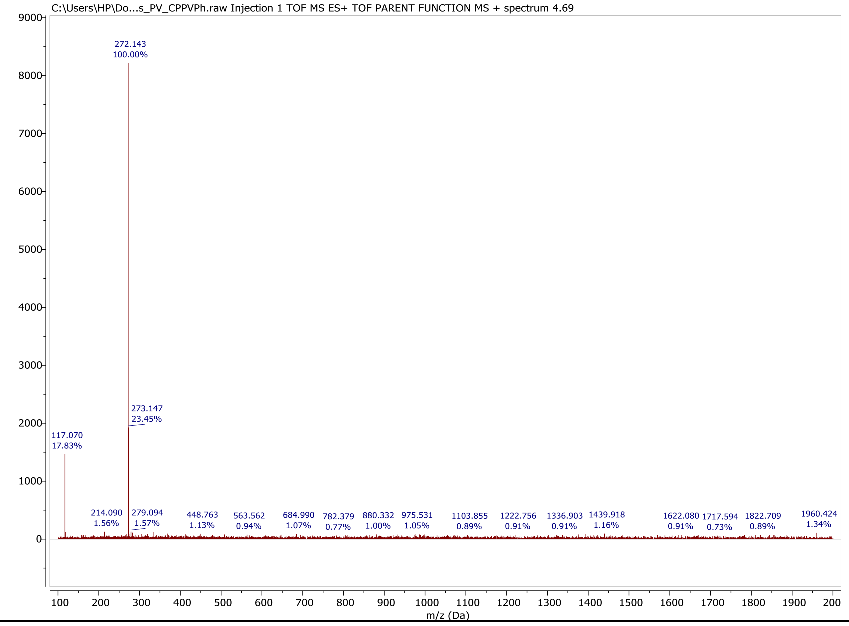

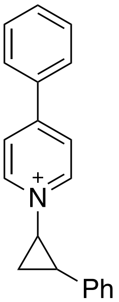

Exact Mass: 272.143

Figure S15. LC-MS/MS results for the reaction (anaerobic) of *trans*-**4c** with **3MLF.**

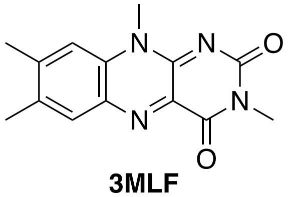

3MLF

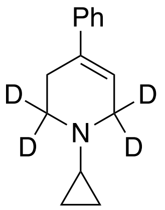

4a$_{D4}$

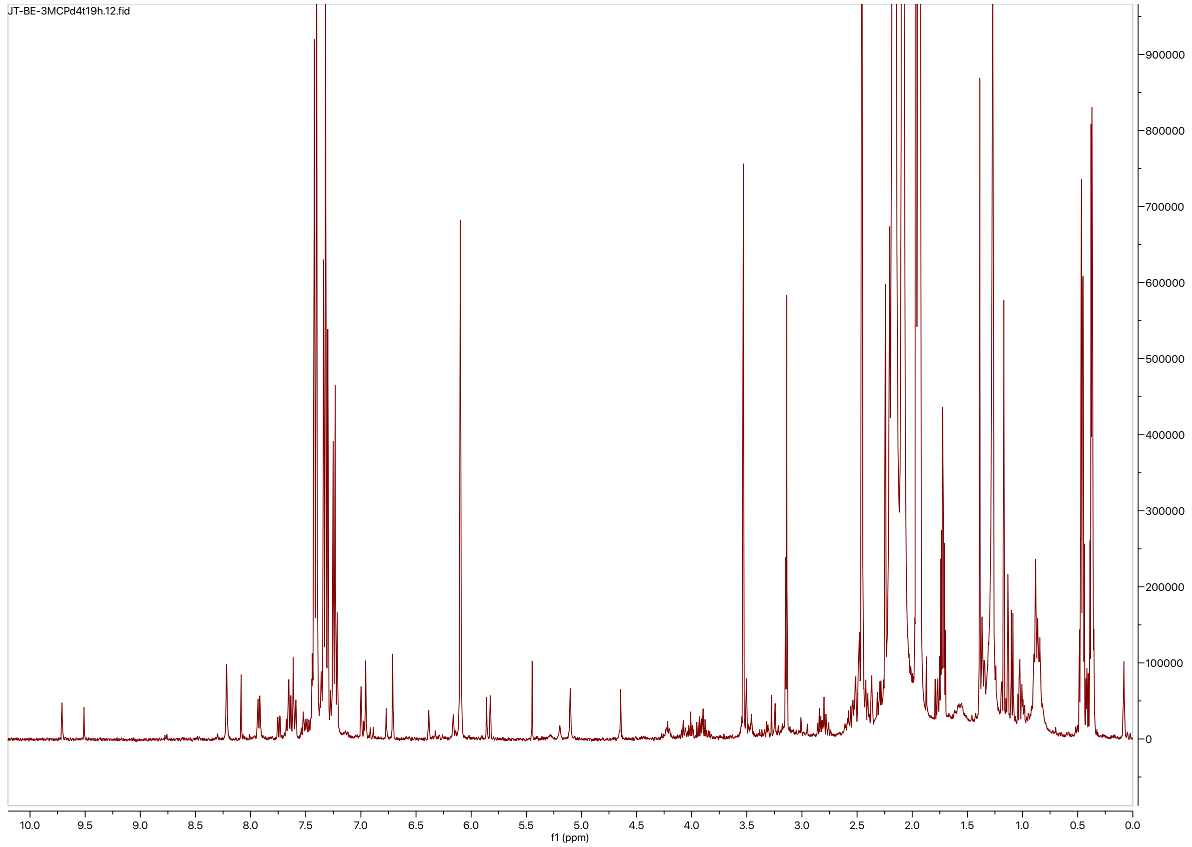

Figure S16. 400 MHz ^{1}H NMR spectrum for the reaction (anaerobic) of **4a$_{D4}$** with **3MLF** (t = 19 h) in CD_3CN.

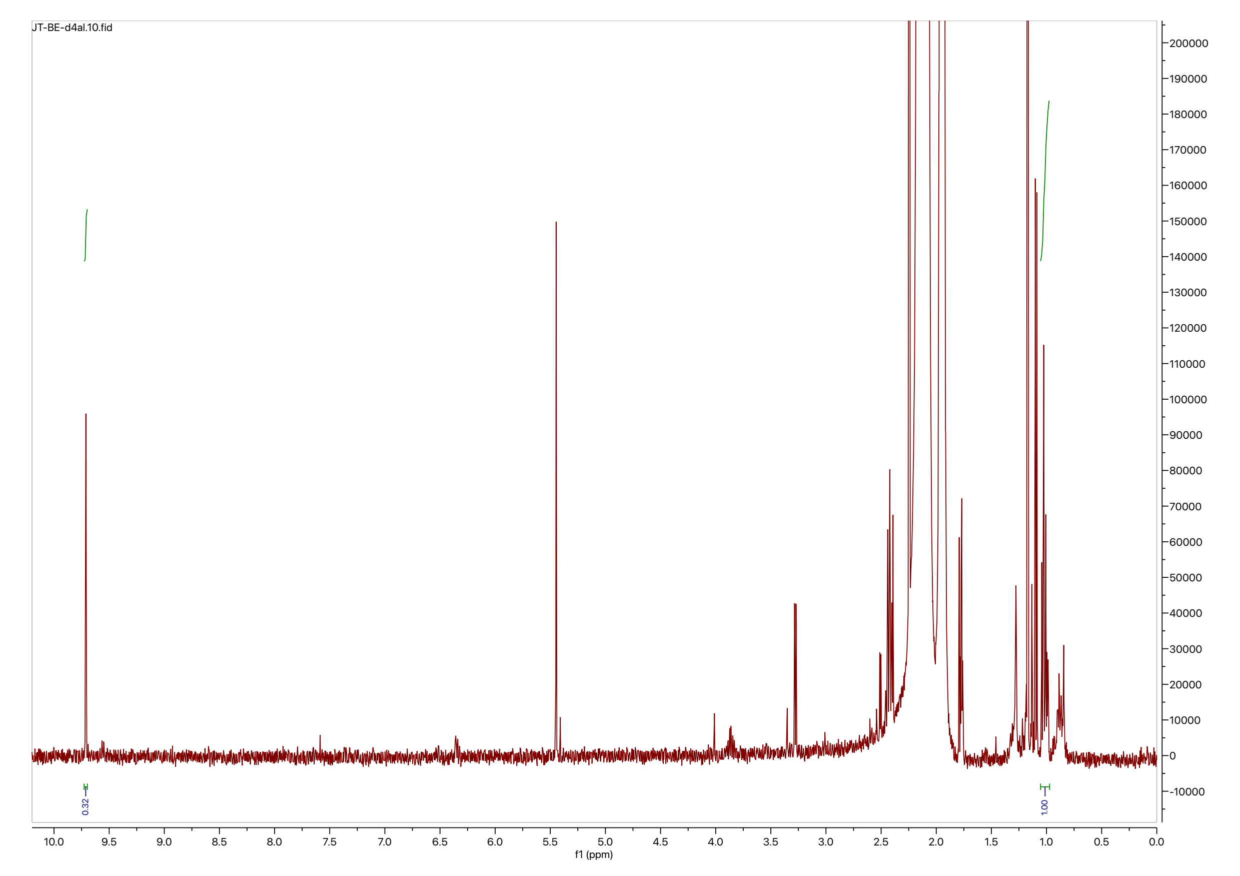

Figure S17. 400 MHz ^{1}H NMR spectrum for the reaction (anaerobic) of **4a$_{D4}$** with **3MLF** (distillate) in CD_3CN.

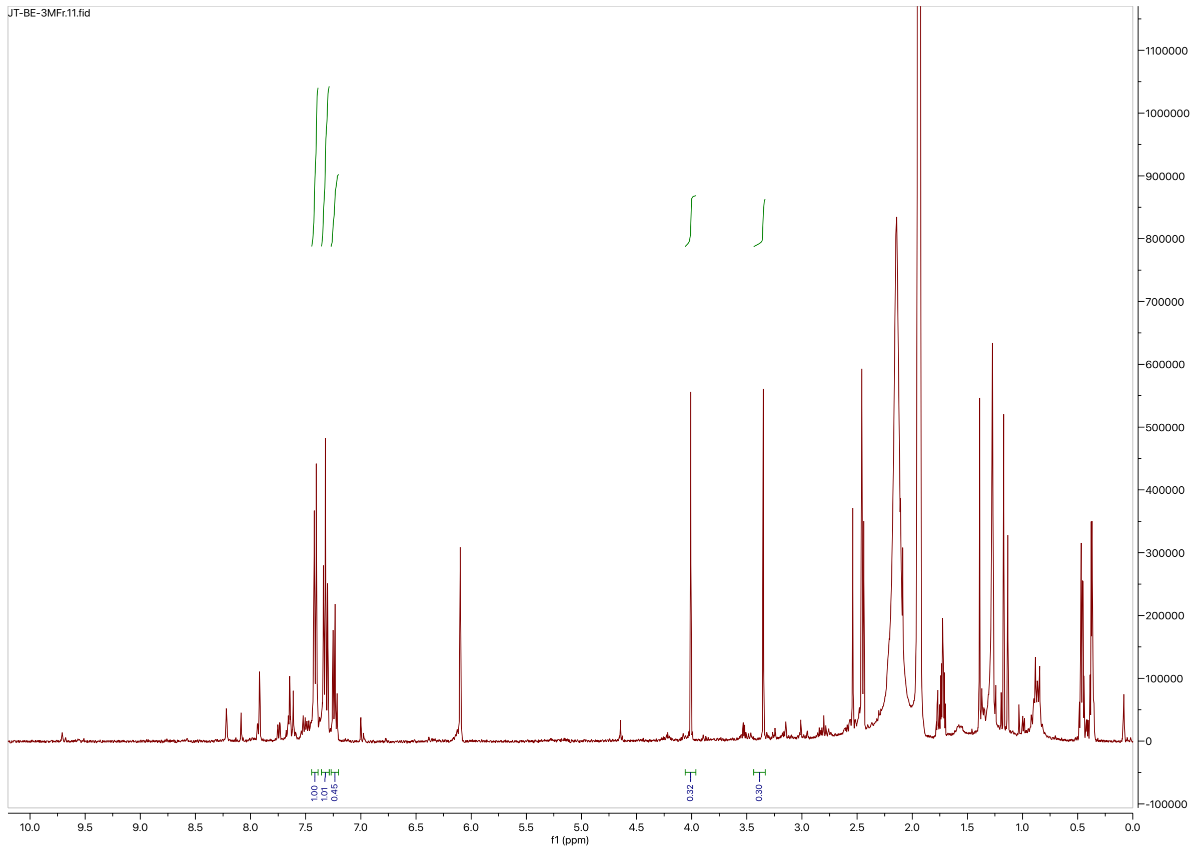

Figure S18. 400 MHz ^{1}H NMR spectrum for the reaction (anaerobic) of **4a$_{D4}$** with **3MLF** (residue after distillation) in CD_3CN.

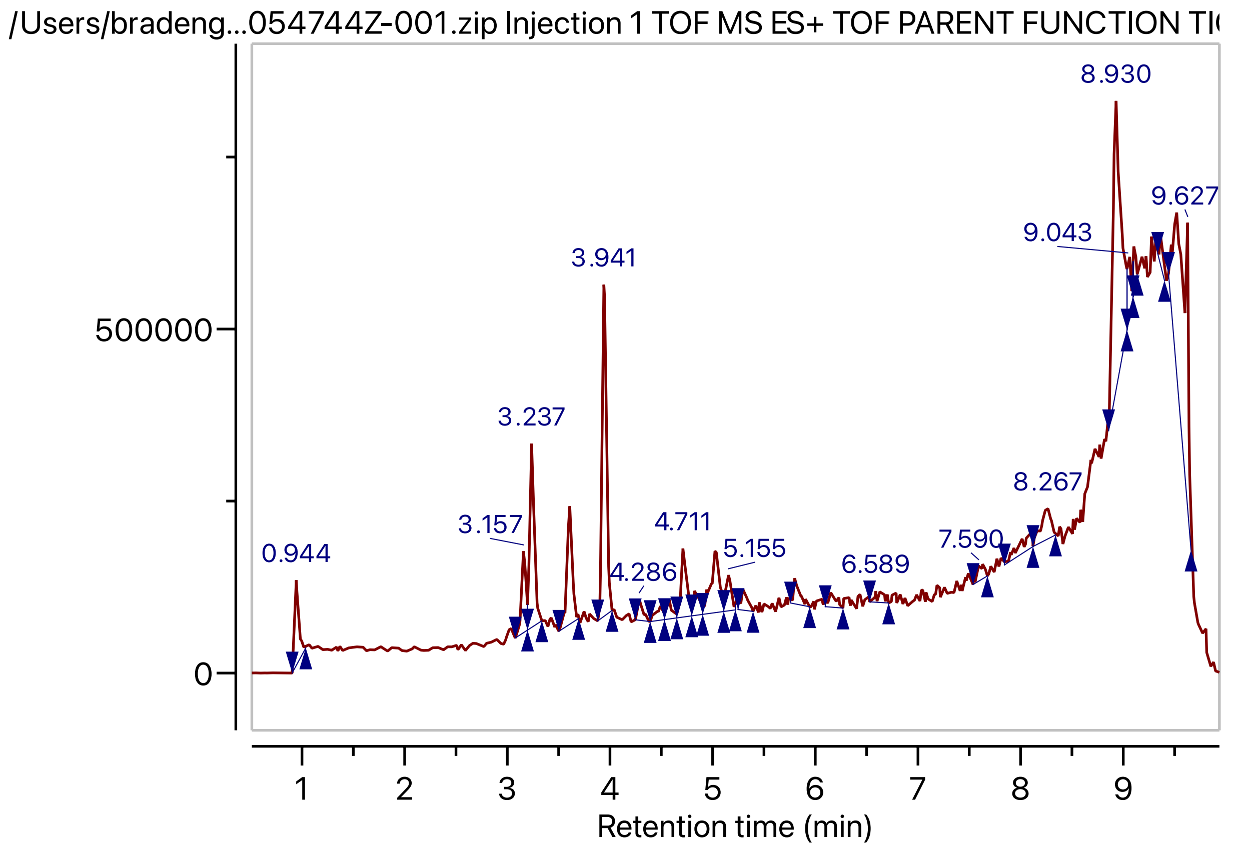

a)

b) 3.16 min

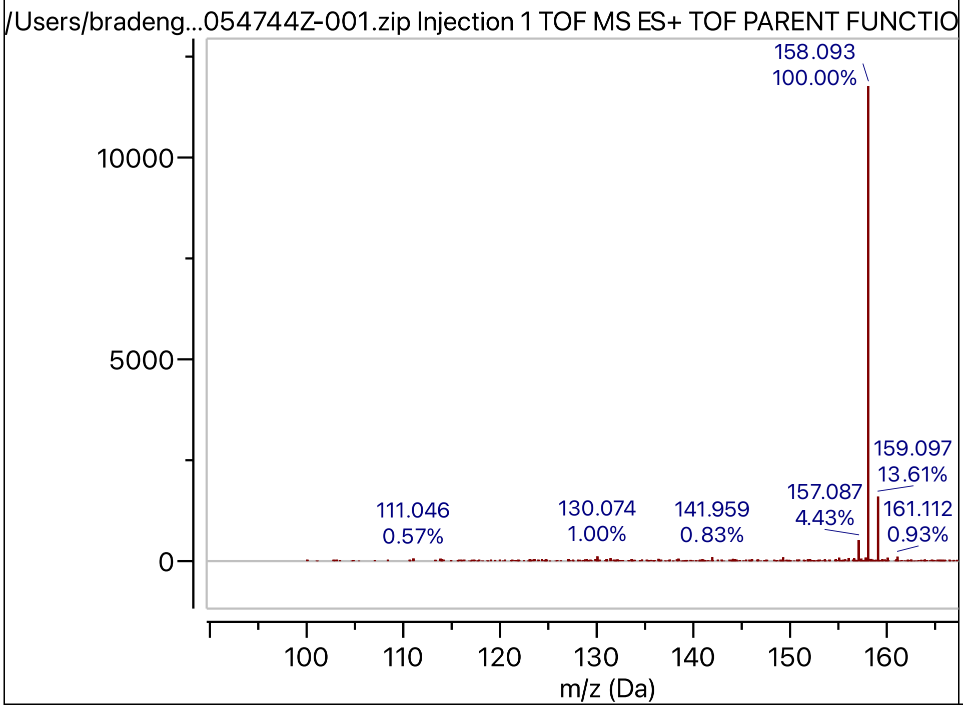

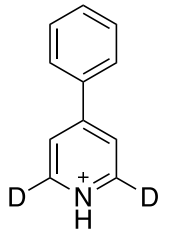

Exact Mass: 158.093

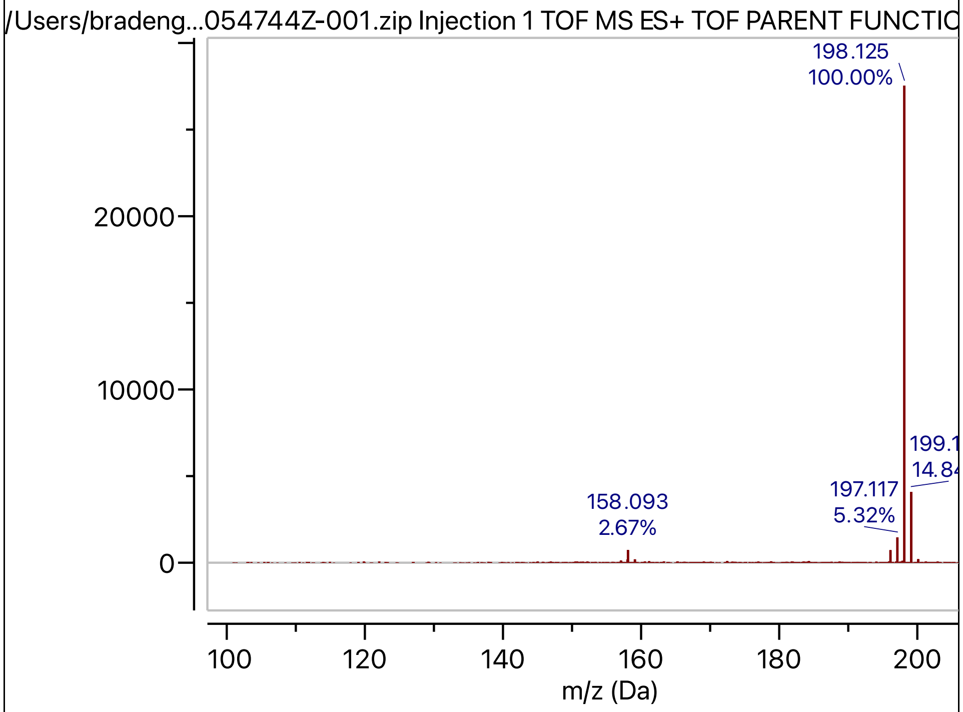

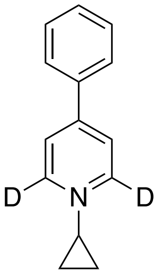

Exact Mass: 198.125

c) 3.24 min

d) 3.61 min

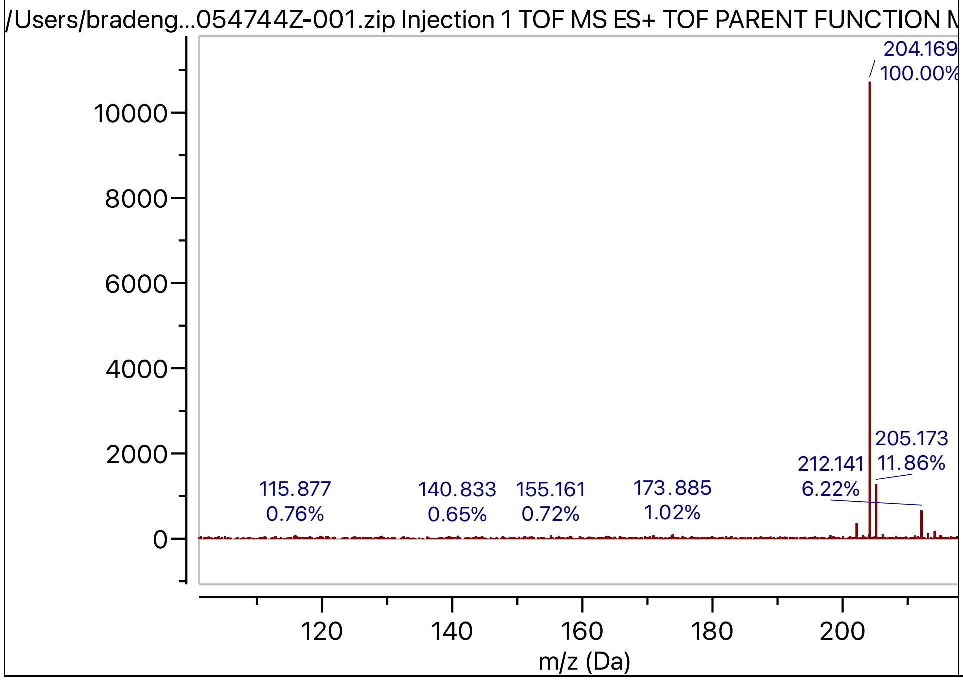

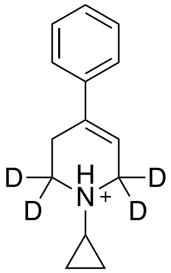

Exact Mass: 204.168

e) 3.94 min

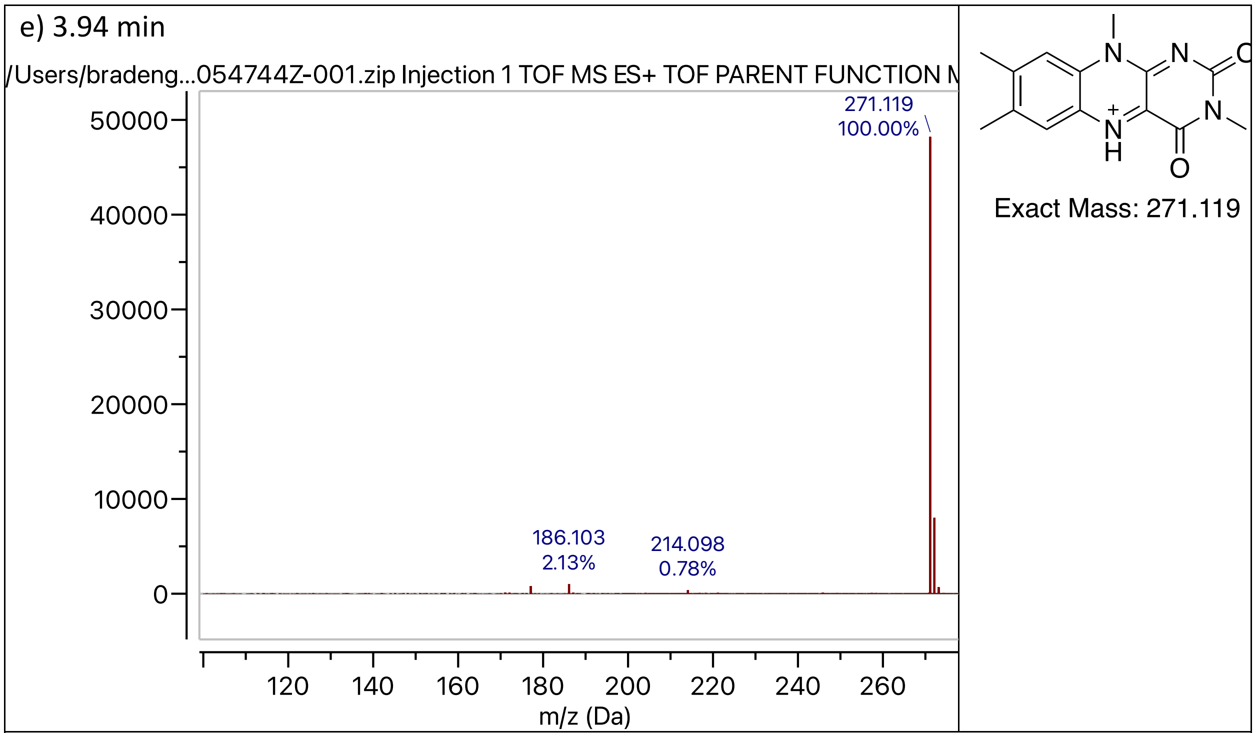

Figure S19. LC-MS/MS results for the reaction (anaerobic) of **4a**$_{\mathbf{D4}}$ with **3MLF**.

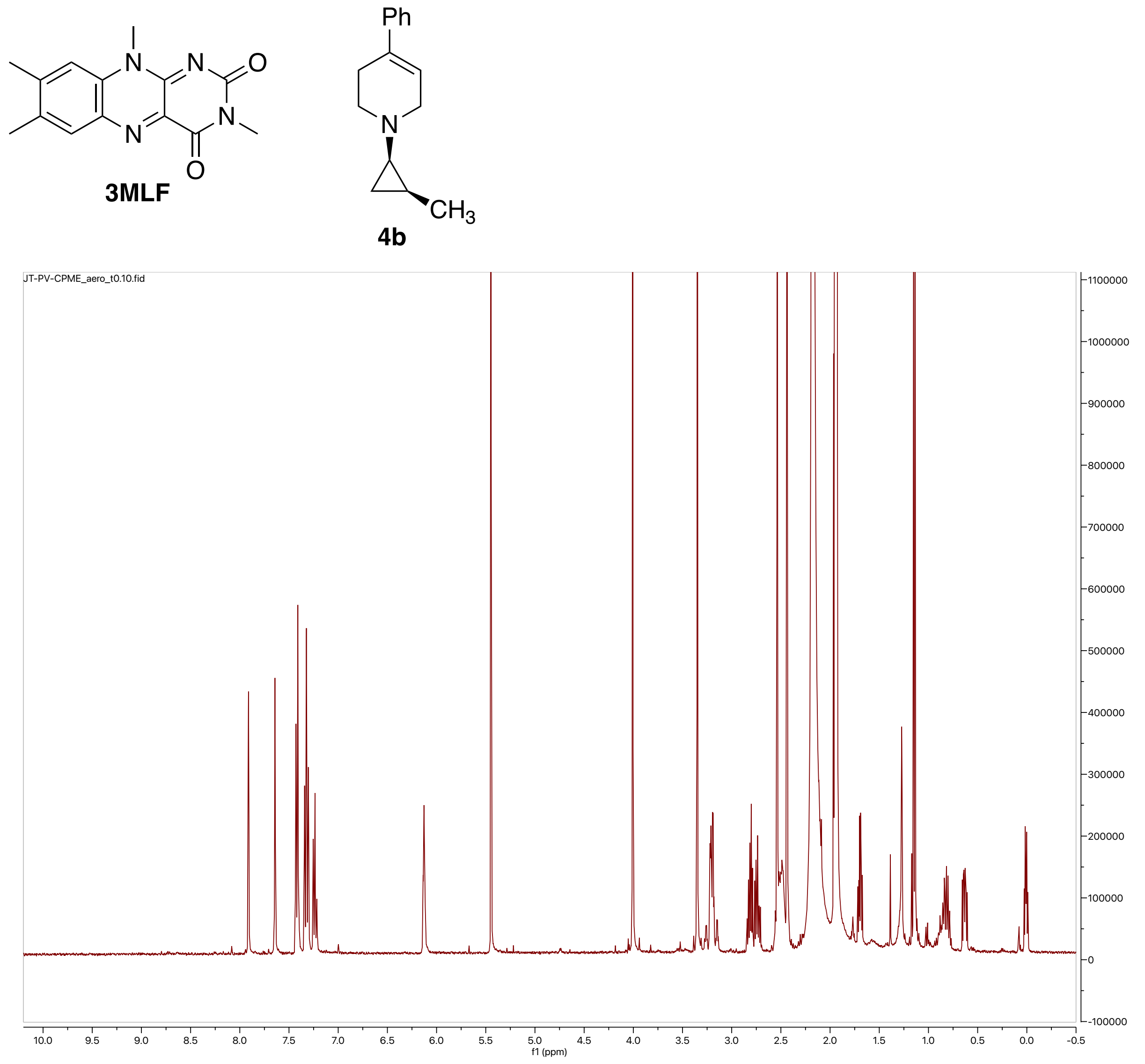

Figure S20. 400 MHz ^{1}H NMR spectrum for the reaction (aerobic) of *cis*-**4b** with **3MLF** (t = 0) in CD_3CN.

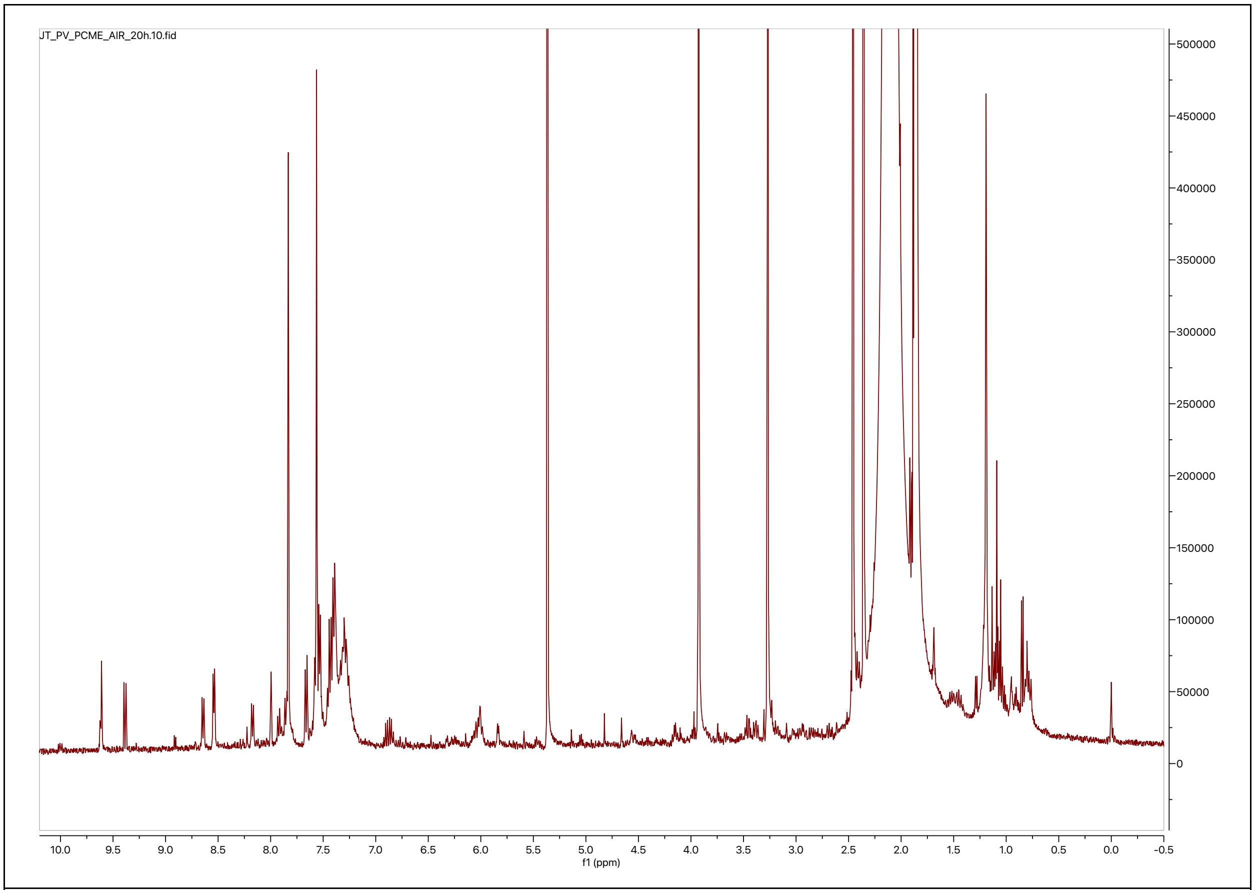

Figure S21. 400 MHz ^{1}H NMR spectrum for the reaction (aerobic) of *cis*-**4b** with **3MLF** (t = 20 h) in CD_3CN.

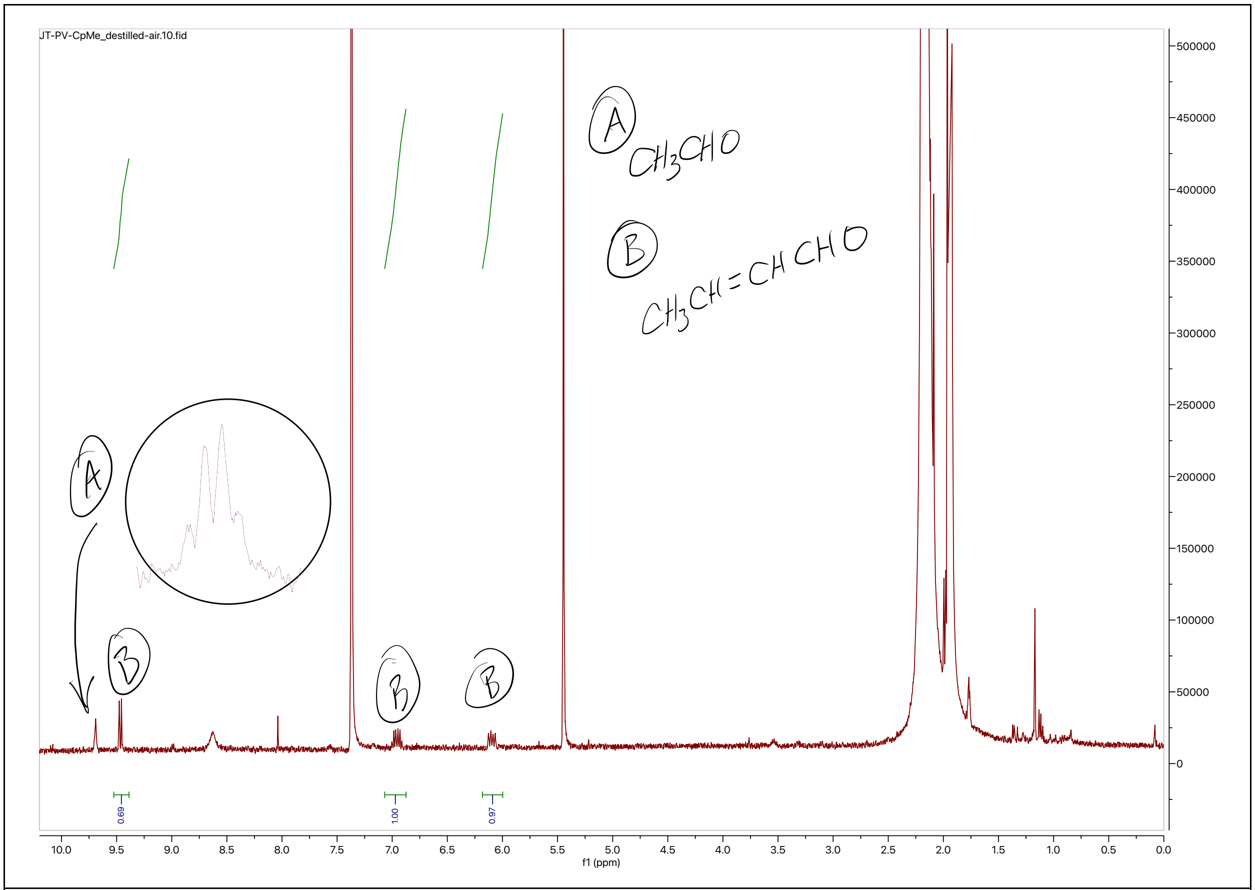

Figure S22. 400 MHz ^{1}H NMR spectrum for the reaction (aerobic) of *cis*-**4b** with **3MLF** (distillate) in CD_3CN.

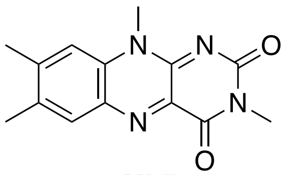

3MLF

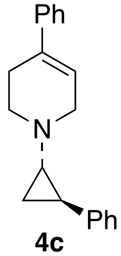

4c

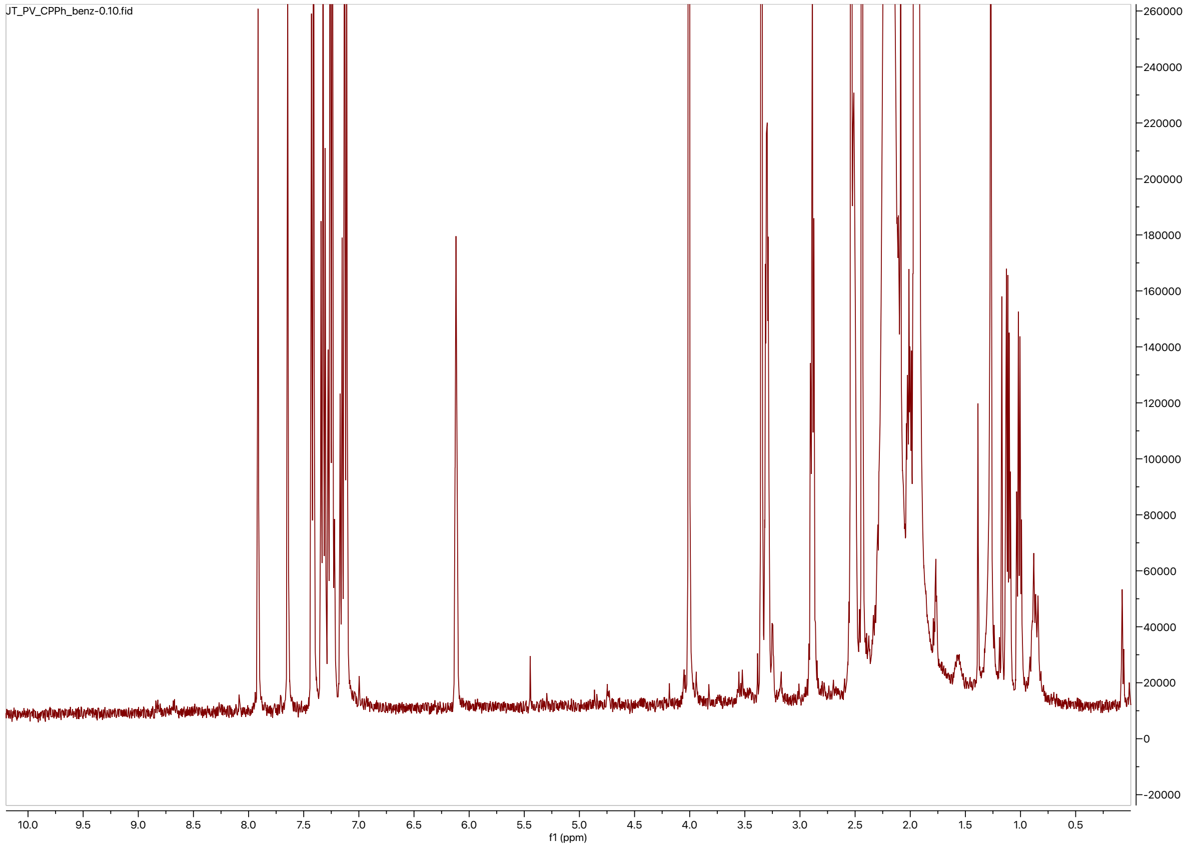

Figure S23. 400 MHz ^{1}H NMR spectrum for the reaction (aerobic) of *trans*-**4c** with **3MLF** (t = 0) in CD_3CN.

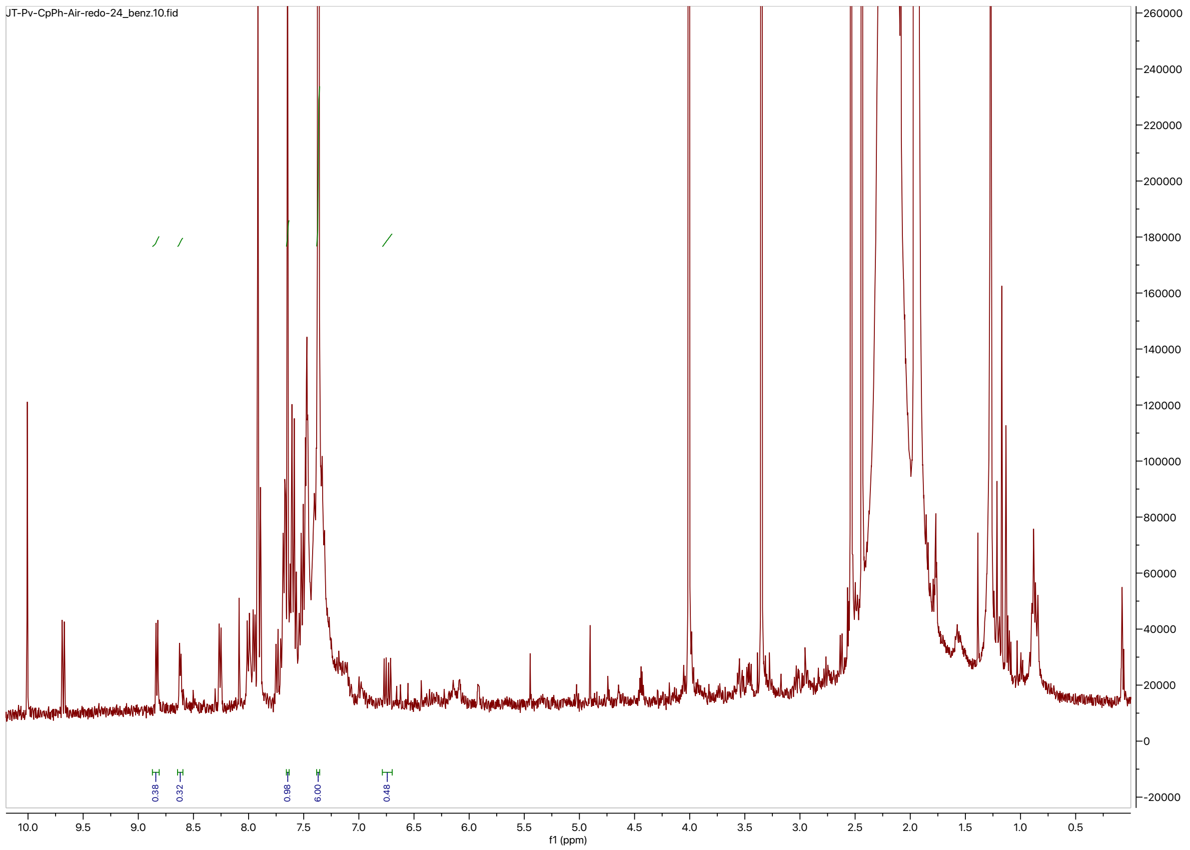

Figure S24. 400 MHz ^{1}H NMR spectrum for the reaction (aerobic) of *trans*-**4c** with **3MLF** (t = 24 h) in CD_3CN.

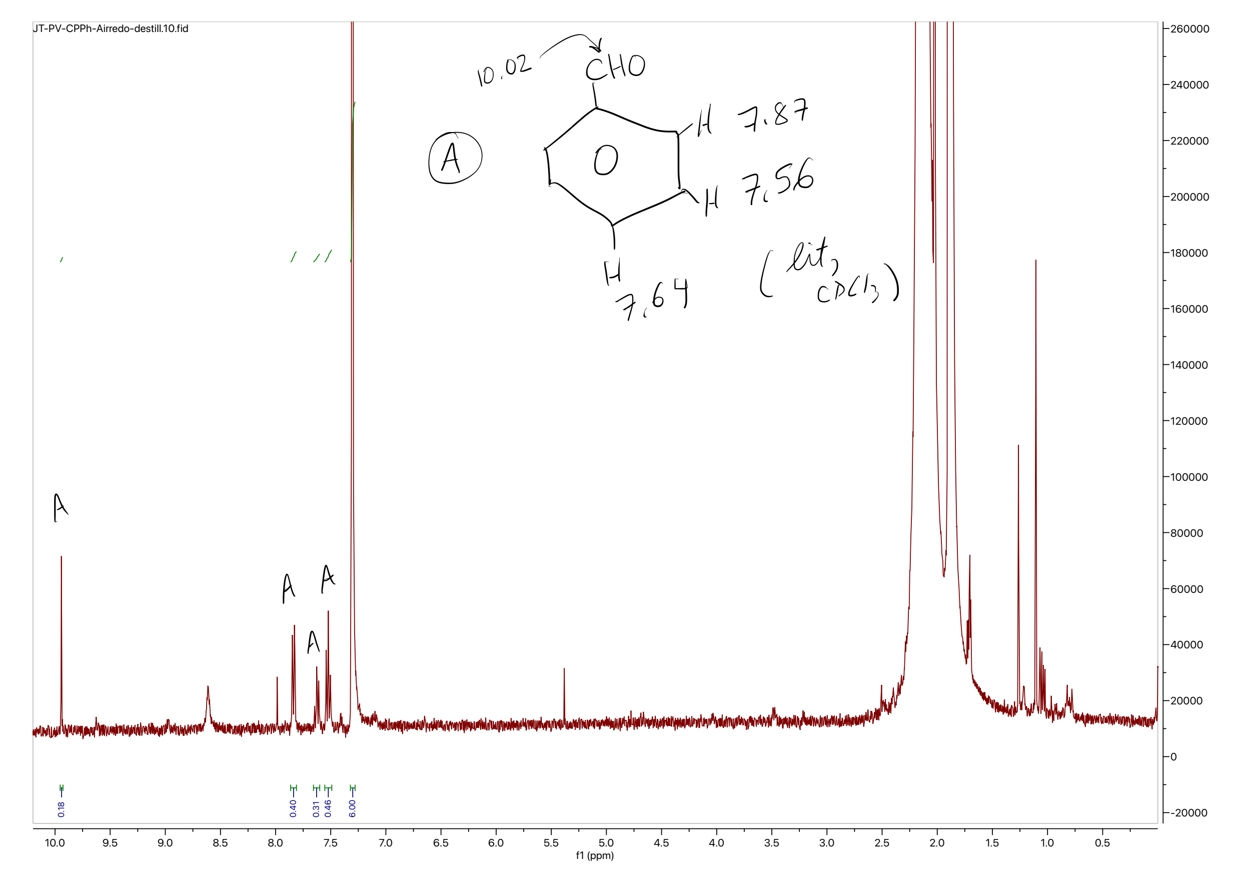

Figure S25. 400 MHz ^{1}H NMR spectrum for the reaction (aerobic) of *trans*-**4c** with **3MLF** (distillate) in CD_3CN.

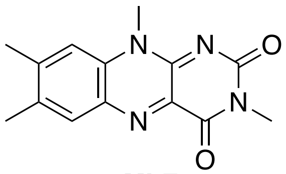

3MLF

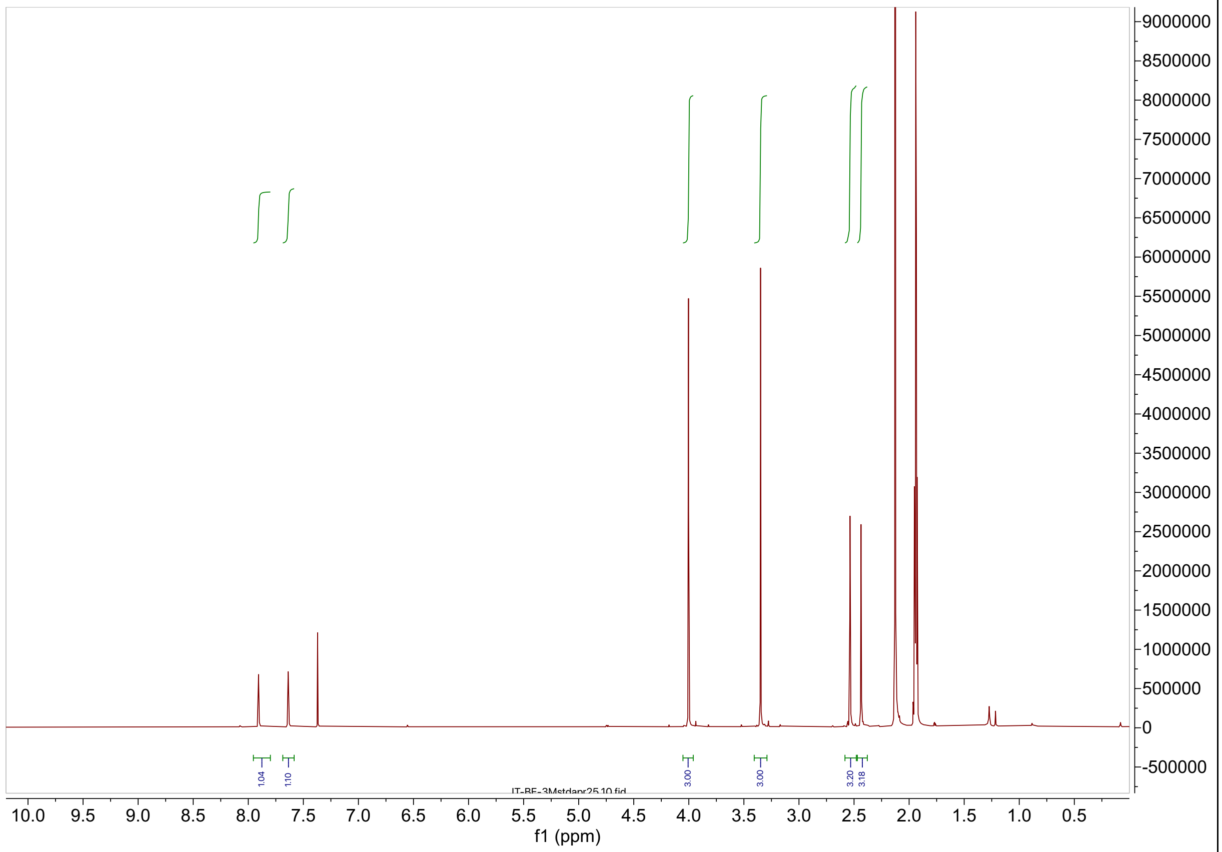

Figure S26. 400 MHz ^{1}H NMR spectrum of 3-methyllumiflavin (**3MLF**) in CD_3CN.

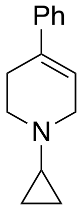

4a

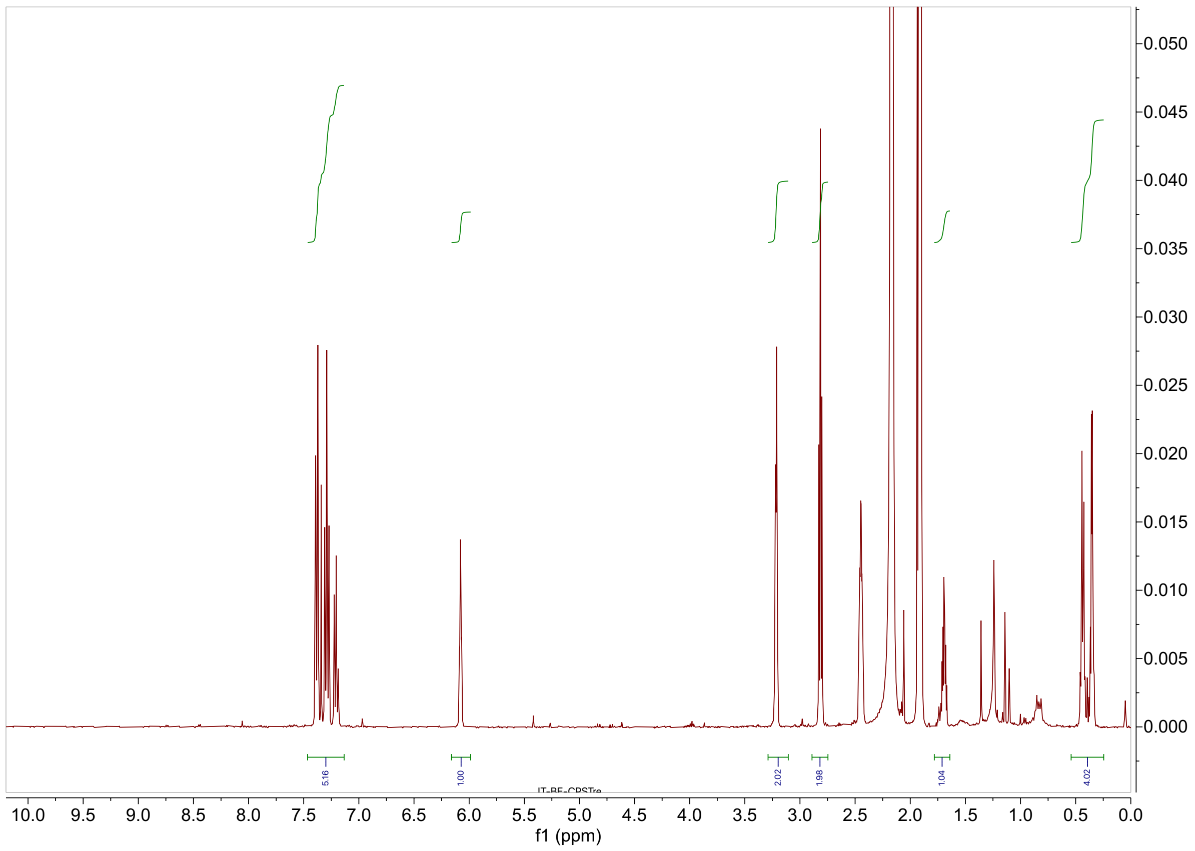

Figure S27. 400 MHz ^{1}H NMR spectrum of **4a** in CD_3CN.

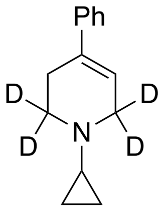

4a$_{D4}$

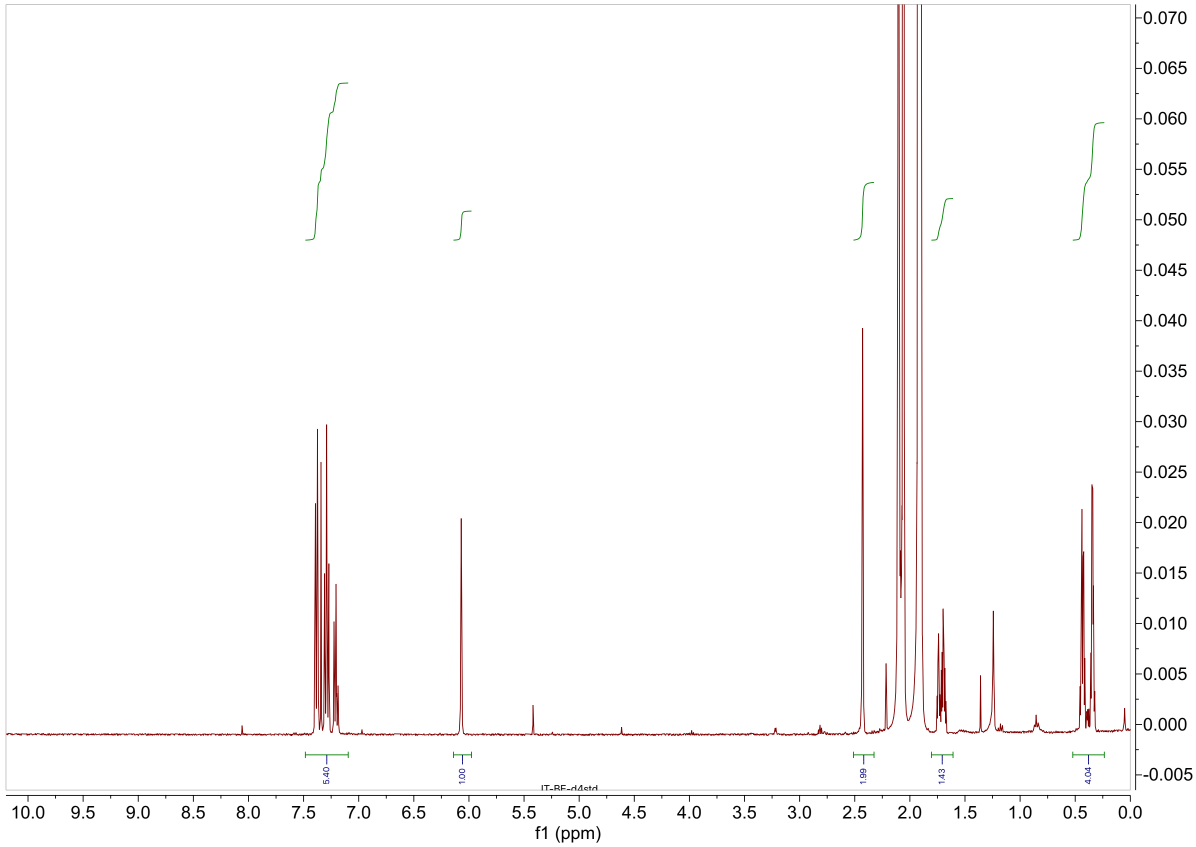

Figure S28. 400 MHz ^{1}H NMR spectrum of **4a$_{D4}$** in CD_3CN.

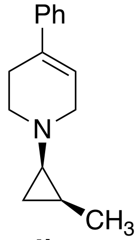

4b

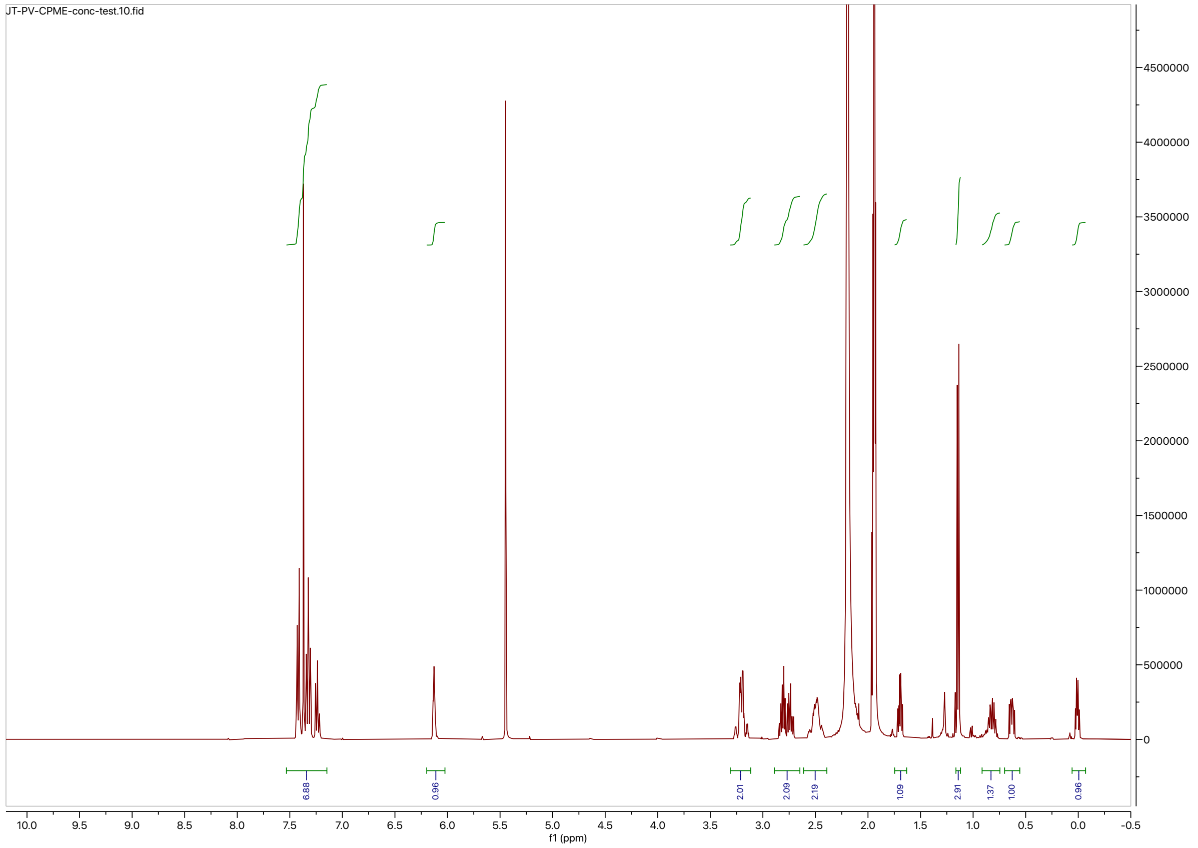

Figure S29. 400 MHz ^{1}H NMR spectrum of *cis*-**4b** in CD_3CN.

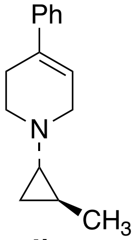

4b

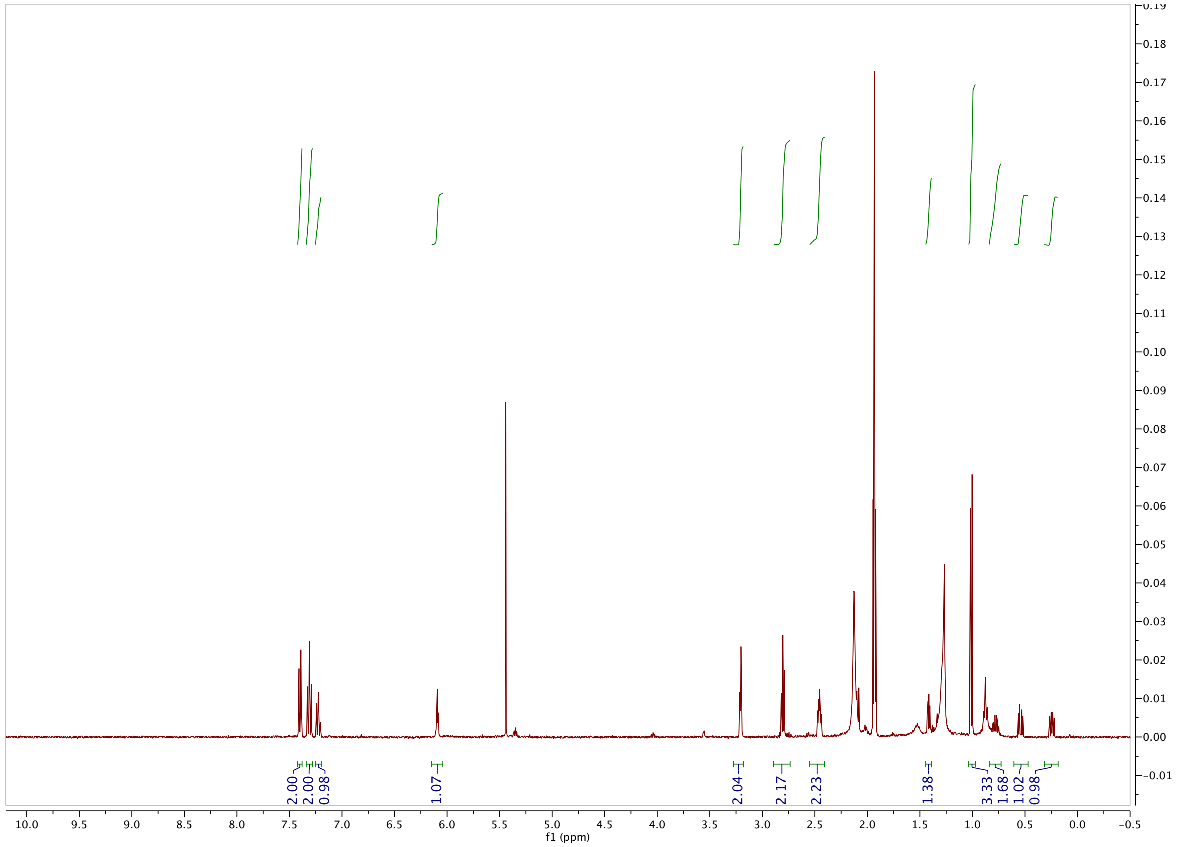

Figure S30. 400 MHz ^{1}H NMR spectrum of *trans*-**4b** in CD_3CN.

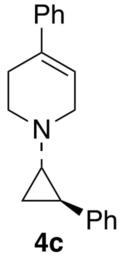

4c

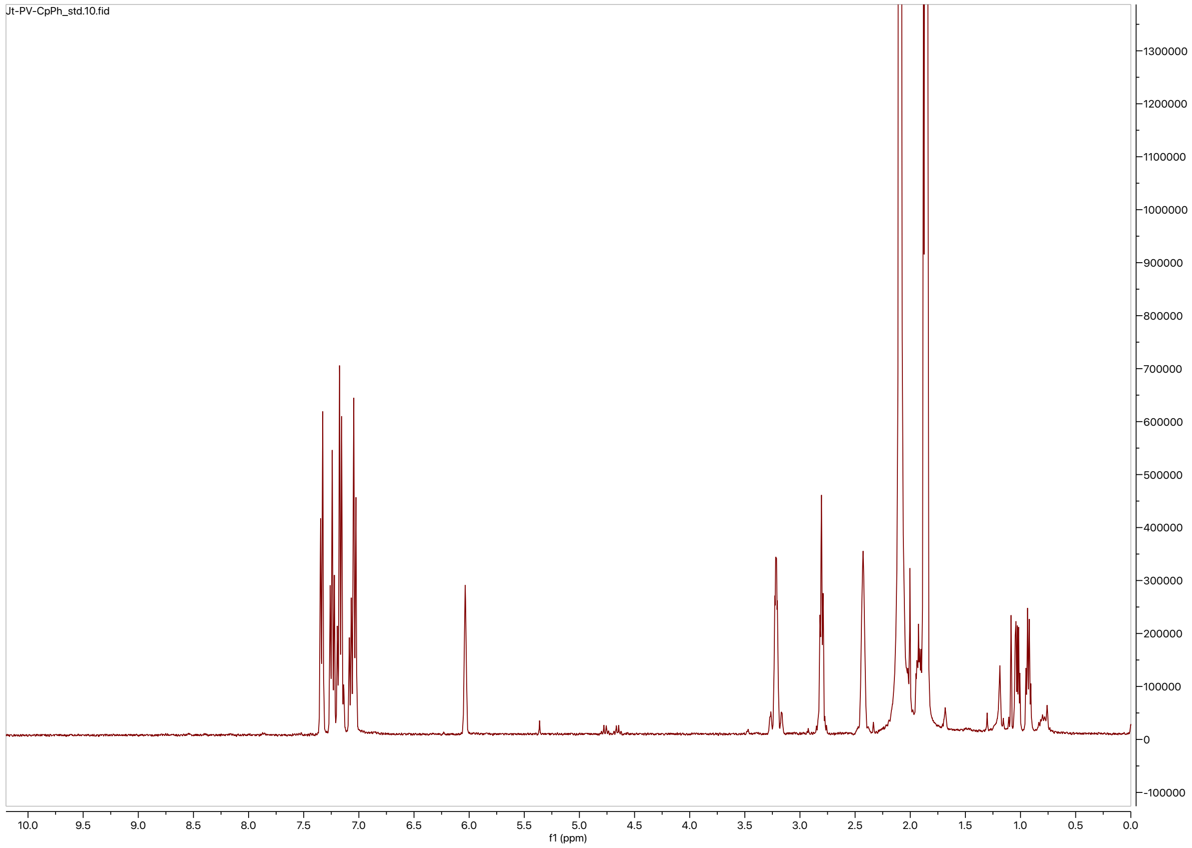

Figure S31. 400 MHz ^{1}H NMR spectrum of *trans*-**4c** in CD_3CN.

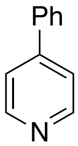

6

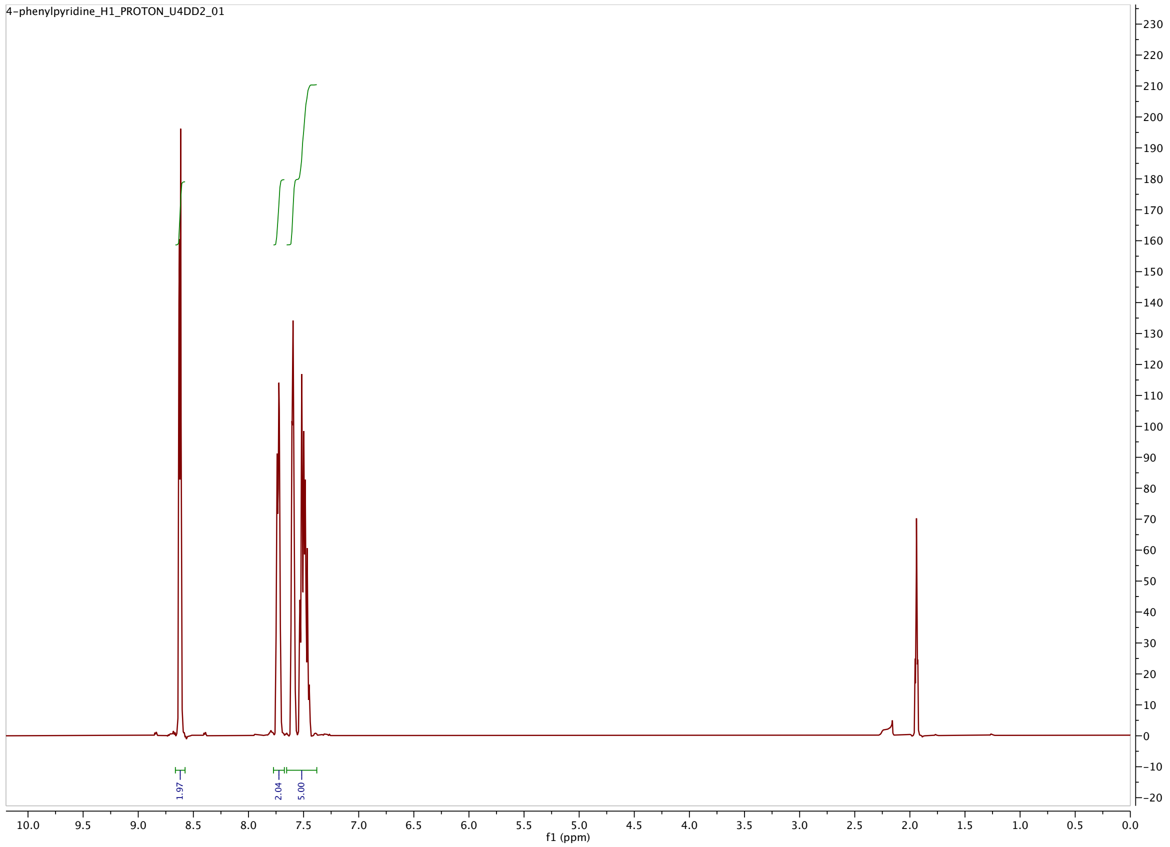

Figure S32. 400 MHz ^{1}H NMR spectrum of 4-phenylpyridine (**6**) in CD_3CN.

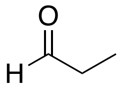

7a

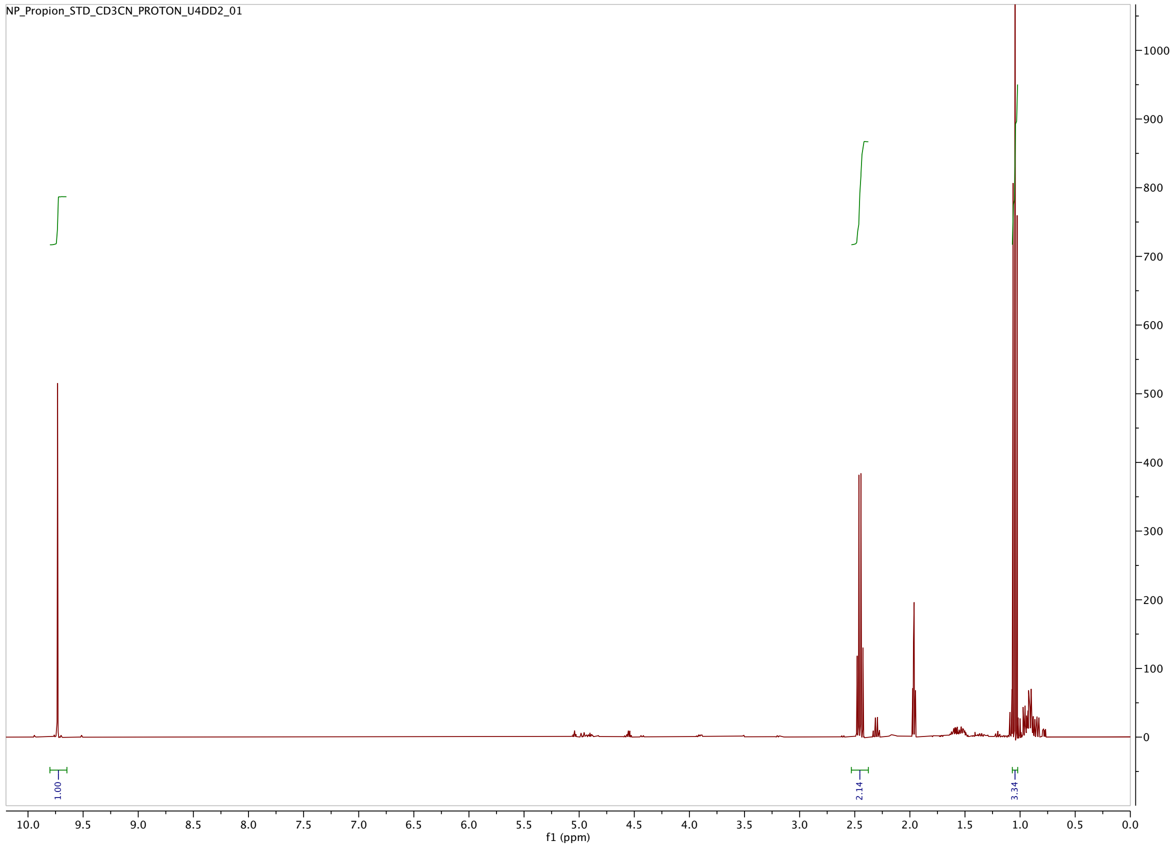

Figure S33. 400 MHz ^{1}H NMR spectrum of propionaldehyde (**7a**) in CD_3CN.

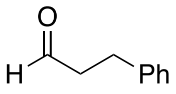

7c

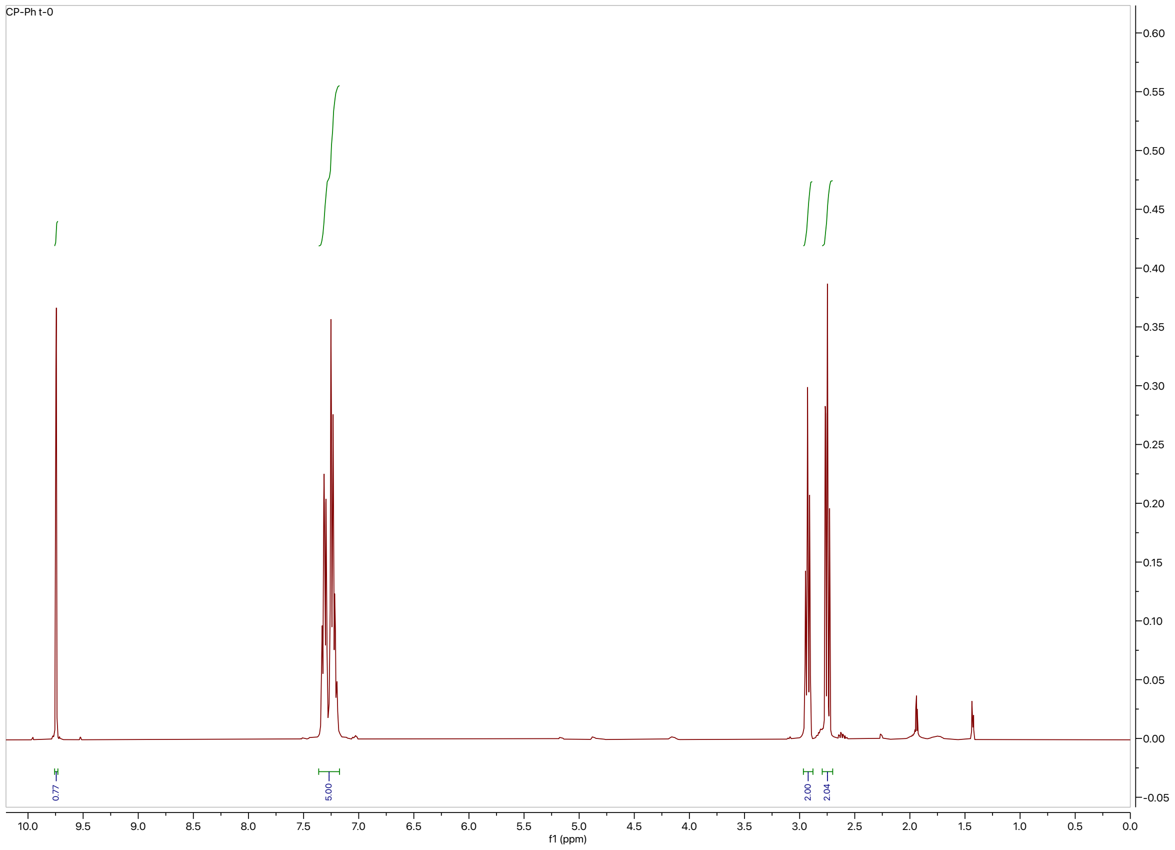

Figure S34. 400 MHz ^{1}H NMR spectrum of 3-phenylpropanal (**7c**) in CD_3CN.

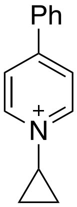

5a

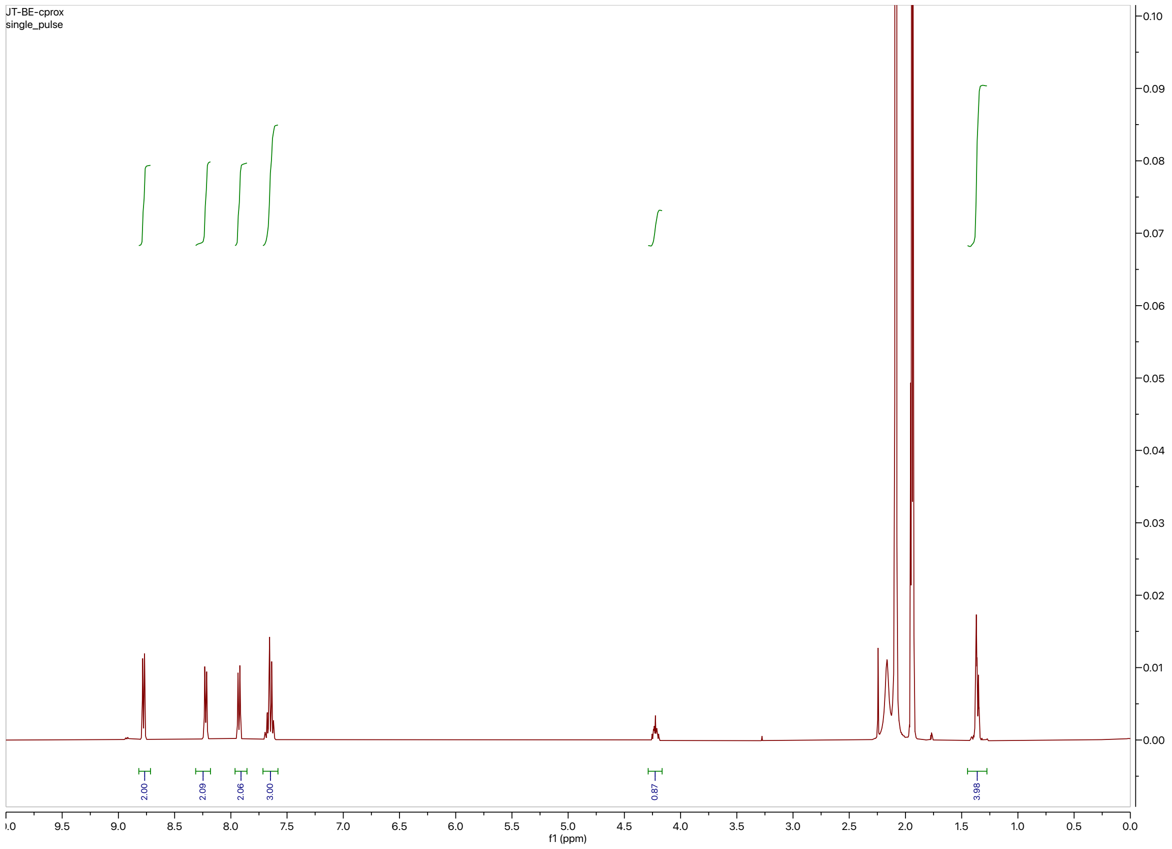

Figure S35. 400 MHz ^{1}H NMR spectrum of **5a** in CD_3CN.

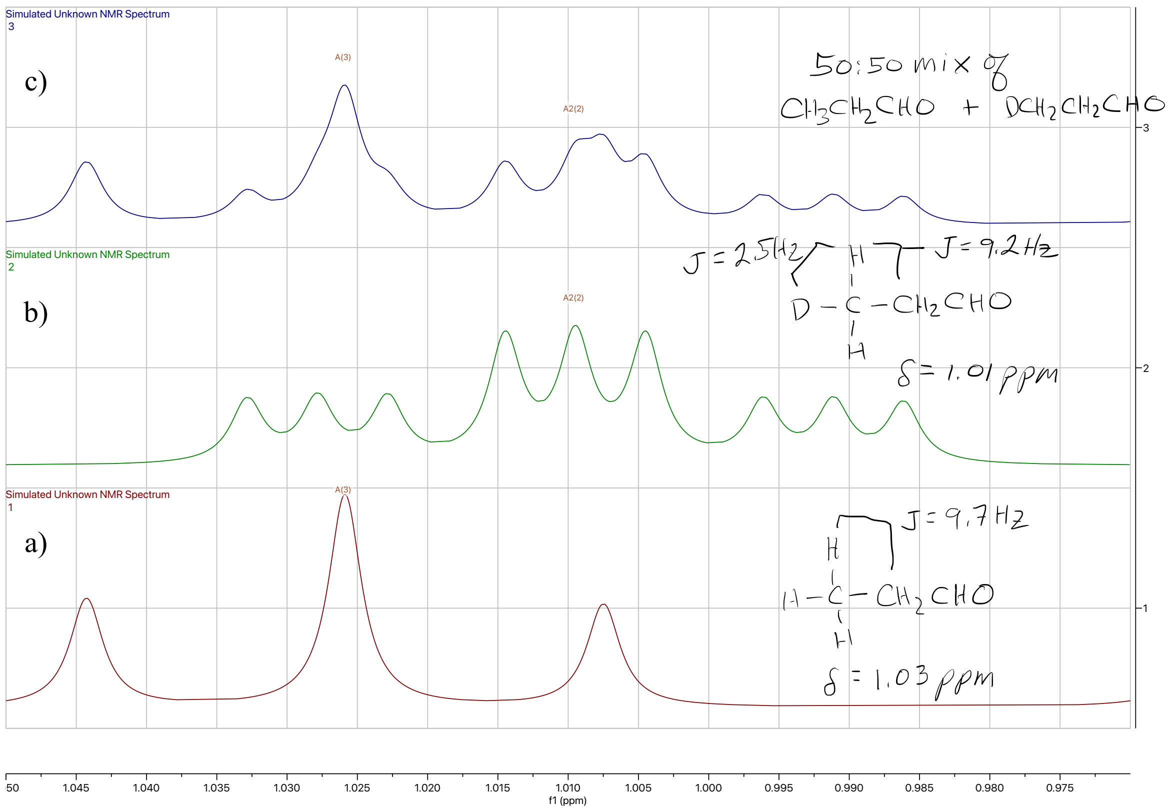

Figure S36. Details of the ^{1}H NMR simulation shown in Figure 8 of the manuscript (methyl region): a) CH_3CH_2CHO, b) DCH_2CH_2CHO, c) 50:50 mixture in CD_3CN.

Computed energies used to assess the energetics of electron transfer between 1,2,3,6-tetrahydropyridine radical cation and itself, **MPTP**, and **MMTP** (Table 1). Geometry optimizations were performed using M062X/6-31G(d) followed by single point calculations at the level indicated.

Core Structure = 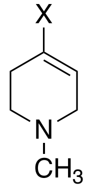

X	Energy (hartrees)		
		**M06-2X/cc-PVTZ**	**CAM-B3LYP/def2-TZVP**
N-methylpyrrole (s-*cis*)	neutral	-538.2256466	-538.1671367
	radical cation	-537.9630065	-537.9104248
N-methylpyrrole (s-*trans*)	neutral	-538.2248934	-538.166443
	radical cation	-537.9644541	-537.9116441
phenyl	neutral	-520.9897704	-520.9268656
	radical cation	-520.7093473	-520.652667
H	neutral	-289.9484876	-289.922987
	radical cation	-289.6650525	-289.6453254

XYZ coordinates for optimized structures pertinent to Table 1	
**Structure**	**XYZ coordinates**
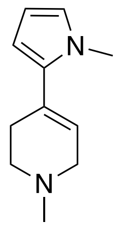<br>**MMTP** (s-*cis*)	6 -4.332782 0.069090 -0.101647<br>7 -2.907173 -0.122049 -0.301362<br>6 -2.204807 1.153244 -0.381358<br>1 -2.494778 1.830218 0.455619<br>1 -2.525068 1.666894 -1.299787<br>6 -0.706868 1.004062 -0.390291<br>1 -0.161583 1.889182 -0.703052<br>6 -0.060725 -0.133472 -0.069790<br>6 -0.879290 -1.359813 0.301912<br>6 -2.307383 -0.990563 0.705194<br>1 -2.917017 -1.898137 0.786897<br>1 -2.301480 -0.515295 1.708533<br>1 -0.912259 -2.050905 -0.551596<br>1 -0.392035 -1.897062 1.124214<br>6 1.391812 -0.320285 -0.092244<br>6 2.084269 -1.506739 -0.314099<br>1 1.624462 -2.465682 -0.510690<br>6 3.471744 -1.229083 -0.274676<br>6 3.602139 0.116990 -0.011840<br>1 4.482910 0.729608 0.122721<br>7 2.349105 0.674064 0.098070<br>6 2.131181 2.058215 0.481227<br>1 3.025060 2.424074 0.992945<br>1 1.281727 2.130739 1.163991<br>1 1.940268 2.701510 -0.386416<br>1 4.282657 -1.930473 -0.417194<br>1 -4.842616 -0.900441 -0.120051<br>1 -4.739903 0.682073 -0.914143<br>1 -4.579606 0.569717 0.856721
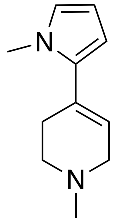<br>**MMTP** (s-*trans*)	6 -4.317488 0.111865 -0.066276<br>7 -2.875403 0.180029 -0.226790<br>6 -2.323288 -1.109280 -0.630305<br>1 -2.676180 -1.333429 -1.647910<br>1 -2.708243 -1.936971 0.008401<br>6 -0.820953 -1.139389 -0.597960<br>1 -0.348799 -2.005553 -1.056094<br>6 -0.060144 -0.190150 -0.026265<br>6 -0.733755 1.005688 0.629999<br>6 -2.198431 0.700907 0.956622<br>1 -2.251294 -0.006930 1.809296<br>1 -2.709682 1.618700 1.270190<br>1 -0.199902 1.274769 1.550545<br>1 -0.697253 1.883785 -0.027987<br>6 1.398990 -0.358811 0.063138

	6 2.118819 -1.490329 0.422150<br>1 1.675927 -2.431590 0.719043<br>6 3.500030 -1.170943 0.376511<br>6 3.593783 0.147529 -0.011083<br>1 4.456196 0.777933 -0.178476<br>7 2.323436 0.646005 -0.195249<br>6 2.040708 1.979318 -0.698048<br>1 2.969605 2.417381 -1.070191<br>1 1.636564 2.633172 0.082663<br>1 1.324748 1.933923 -1.524233<br>1 4.329683 -1.825008 0.608891<br>1 -4.713339 1.107997 0.160248<br>1 -4.638210 -0.578093 0.740291<br>1 -4.775675 -0.230753 -1.001306
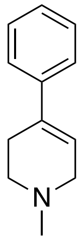<br>**MPTP**	6 0.907537 -1.252484 0.256333<br>6 0.169540 0.044097 -0.021232<br>6 -1.315195 0.030981 -0.013546<br>6 -2.060906 1.169995 0.341998<br>6 -3.453547 1.152570 0.332088<br>6 -4.142144 -0.009066 -0.025286<br>6 -3.419730 -1.152536 -0.366806<br>6 -2.025100 -1.133817 -0.355427<br>1 -1.482181 -2.032559 -0.632734<br>1 -3.941799 -2.064754 -0.644407<br>1 -5.228723 -0.024228 -0.028199<br>1 -4.003571 2.045792 0.617514<br>1 -1.540816 2.070900 0.654689<br>6 0.877615 1.156190 -0.278248<br>6 2.379893 1.214149 -0.300413<br>7 3.000770 -0.105481 -0.300724<br>6 2.359859 -0.993407 0.663354<br>1 2.389627 -0.577402 1.691738<br>1 2.915172 -1.938296 0.684886<br>6 4.438502 -0.015283 -0.114655<br>1 4.728855 0.414597 0.865372<br>1 4.886020 -1.011987 -0.195235<br>1 4.872370 0.615827 -0.898930<br>1 2.714158 1.754953 -1.198025<br>1 2.722409 1.828944 0.563639<br>1 0.370476 2.093111 -0.497962<br>1 0.895923 -1.892014 -0.637435<br>1 0.398753 -1.812846 1.051207
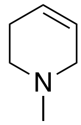<br>*N*-methyl-1,2,3,6-tetrahydropyridine	6 -1.256340 -1.219920 -0.146323<br>6 -1.902850 0.138484 -0.074111<br>1 -2.989418 0.191296 -0.120996<br>6 -1.180993 1.253884 0.048779<br>6 0.323947 1.250422 0.091007

	7 0.890696 -0.035490 -0.307381<br>6 0.199139 -1.154190 0.329424<br>1 0.223237 -1.074569 1.436142<br>1 0.725670 -2.078442 0.064471<br>6 2.326160 -0.070284 -0.089262<br>1 2.611844 0.019232 0.978605<br>1 2.736923 -1.010772 -0.472898<br>1 2.801799 0.754220 -0.633007<br>1 0.715018 2.022877 -0.587196<br>1 0.656623 1.549083 1.110983<br>1 -1.665089 2.225674 0.126808<br>1 -1.293448 -1.600119 -1.177616<br>1 -1.812414 -1.940421 0.469298
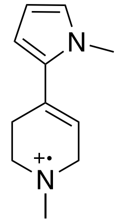<br>**MMTP** (s-*cis*)$^{\bullet+}$	6 -4.363341 0.112662 -0.087365<br>7 -2.923804 -0.046408 -0.245078<br>6 -2.181333 1.182233 -0.245589<br>1 -2.480442 1.836430 0.608319<br>1 -2.440038 1.778071 -1.137098<br>6 -0.703368 1.058005 -0.165239<br>1 -0.173376 1.995125 -0.269955<br>6 -0.043306 -0.140666 -0.023326<br>6 -0.897009 -1.400805 0.054181<br>6 -2.310416 -1.094024 0.554856<br>1 -2.928130 -1.993976 0.487620<br>1 -2.271059 -0.804820 1.623882<br>1 -0.952537 -1.860644 -0.941059<br>1 -0.441809 -2.132732 0.727339<br>6 1.374745 -0.304382 -0.026269<br>6 2.064630 -1.549759 -0.161531<br>1 1.583694 -2.511835 -0.260584<br>6 3.422641 -1.303227 -0.177967<br>6 3.578515 0.087573 -0.029684<br>1 4.486090 0.673200 0.034030<br>7 2.366331 0.686625 0.054504<br>6 2.215569 2.125089 0.285245<br>1 3.196868 2.535623 0.526218<br>1 1.547539 2.305449 1.130036<br>1 1.838779 2.628605 -0.609236<br>1 4.226568 -2.018447 -0.277671<br>1 -4.857727 -0.839283 -0.298359<br>1 -4.730222 0.854902 -0.802342<br>1 -4.645846 0.436620 0.929000

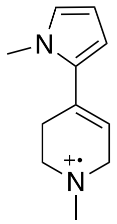<br>**MMTP** (s-*trans*)$^{\bullet+}$	6 -4.349070 0.075038 -0.084248<br>7 -2.899389 0.082829 -0.250349<br>6 -2.319704 -1.234633 -0.285344<br>1 -2.648196 -1.770637 -1.190691<br>1 -2.681947 -1.875101 0.555697<br>6 -0.839005 -1.279573 -0.209521<br>1 -0.397970 -2.261403 -0.348459<br>6 -0.044312 -0.170729 -0.005849<br>6 -0.741705 1.166907 0.176678<br>6 -2.187044 0.975100 0.651517<br>1 -2.189723 0.594578 1.692225<br>1 -2.694362 1.943885 0.661176<br>1 -0.211067 1.781503 0.909167<br>1 -0.750692 1.716886 -0.772938<br>6 1.373388 -0.344381 0.018754<br>6 2.090057 -1.576332 0.190770<br>1 1.632828 -2.543922 0.337938<br>6 3.440137 -1.301030 0.199734<br>6 3.568077 0.088614 0.012089<br>1 4.463379 0.690934 -0.067026<br>7 2.342122 0.660393 -0.084945<br>6 2.162327 2.089742 -0.372191<br>1 3.134211 2.502580 -0.644565<br>1 1.787093 2.622226 0.503799<br>1 1.480302 2.225454 -1.212180<br>1 4.257801 -1.995734 0.329099<br>1 -4.733870 1.089888 -0.215967<br>1 -4.665150 -0.294720 0.906707<br>1 -4.800643 -0.561314 -0.851263
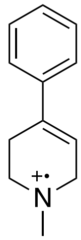<br>**MPTP**$^{\bullet+}$	6 0.920632 -1.260873 0.069727<br>6 0.146434 0.044482 -0.015115<br>6 -1.317886 0.027701 -0.000490<br>6 -2.068396 1.197737 0.276628<br>6 -3.454702 1.172486 0.268583<br>6 -4.134380 -0.016858 -0.024811<br>6 -3.412108 -1.184977 -0.298046<br>6 -2.023810 -1.166763 -0.275821<br>1 -1.484229 -2.081475 -0.497289<br>1 -3.936219 -2.108149 -0.524557<br>1 -5.219967 -0.034815 -0.032407<br>1 -4.012274 2.075202 0.497616<br>1 -1.560862 2.121299 0.534742<br>6 0.864415 1.188818 -0.176132<br>6 2.345393 1.231153 -0.299520<br>7 3.039516 0.000625 -0.061962<br>6 2.322793 -1.045684 0.645498<br>1 2.241723 -0.742838 1.706331

	1 2.915463 -1.963343 0.610271<br>6 4.485909 -0.044929 -0.156271<br>1 4.941330 0.036836 0.842761<br>1 4.799187 -0.996890 -0.597137<br>1 4.844923 0.777378 -0.778167<br>1 2.631756 1.610789 -1.297092<br>1 2.744805 1.999750 0.396362<br>1 0.379434 2.149820 -0.308193<br>1 0.984442 -1.725448 -0.923970<br>1 0.408106 -1.976254 0.719096
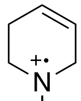<br>*N*-methyl-1,2,3,6-tetrahydropyridine$^{\bullet+}$	6 -1.165478 -1.245453 -0.254136<br>6 -1.890789 0.069443 -0.160216<br>1 -2.971345 0.064388 -0.272394<br>6 -1.245580 1.219273 0.044702<br>6 0.252799 1.314572 0.110038<br>7 0.929006 0.029005 0.078098<br>6 0.193432 -1.167385 0.467356<br>1 0.016509 -1.079512 1.552185<br>1 0.824963 -2.039474 0.287009<br>6 2.332983 -0.054715 -0.272342<br>1 2.858563 -0.665938 0.470418<br>1 2.426805 -0.554535 -1.248028<br>1 2.771261 0.942250 -0.329972<br>1 0.662539 1.922897 -0.709750<br>1 0.584553 1.820202 1.038478<br>1 -1.769963 2.166425 0.127268<br>1 -1.033080 -1.548444 -1.301812<br>1 -1.738050 -2.045702 0.227499

Results obtained from molecular orbital calculations pertaining to the barrier ($\Delta E^{\neq}$)[1] and energetics (ΔE^{o})[2] of ring opening for radical cations generated from **4**(**a** - **c**)

4a$^{\bullet+}$

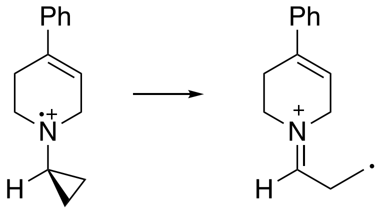

B1

	CAM3-B3LYP/CC-PVDZ		M062X/CC-PVDZ		M062X/DEF2-TZVP//M062X/CC-PVDZ	
	**au**	**kcal/mol**	**au**	**kcal/mol**	**au**	**kcal/mol**
reactant	-597.841264		-597.951851		-598.110617	
transition state ($\Delta E^{\neq}$)	-597.833926	4.6	-597.942275	6.0	-598.101358	5.8
product (ΔE^{o})	-597.840020	0.8	-597.946513	3.3	-598.105581	3.2

4a$^{\bullet+}$

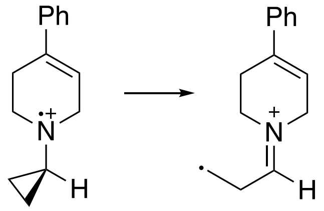

B2

	CAM3-B3LYP/CC-PVDZ		M062X/CC-PVDZ		M062X/DEF2-TZVP//M062X/CC-PVDZ	
	**au**	**kcal/mol**	**au**	**kcal/mol**	**au**	**kcal/mol**
reactant	-597.841104		-597.951573		-598.110393	
transition state ($\Delta E^{\neq}$)	-597.832677	5.3	-597.940595	6.9	-598.099858	6.6
product (ΔE^{o})	-597.839325	1.1	-597.945653	3.7	-598.105075	3.3

[1] $\Delta E^{\neq}$ = E(transition state) - E(reactant).
[2] ΔE^{o} = E(ring-opened product) - E(reactant).

cis-**4b**$^{\bullet+}$

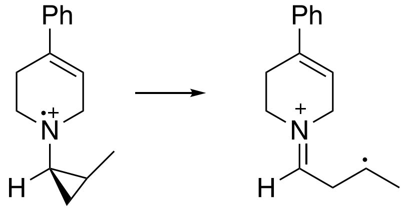

B1

	CAM3-B3LYP/CC-PVDZ		M062X/CC-PVDZ		M062X/DEF2-TZVP//M062X/CC-PVDZ	
	**au**	**kcal/mol**	**au**	**kcal/mol**	**au**	**kcal/mol**
reactant	-637.134055		-637.251207		-637.422756	
transition state ($\Delta E^{\neq}$)	-637.133159	0.6	-637.248136	1.9	-637.418718	2.5
product (ΔE^{o})	-637.138622	-2.9	-637.251858	-0.4	-637.423151	-0.4

cis-**4b**$^{\bullet+}$

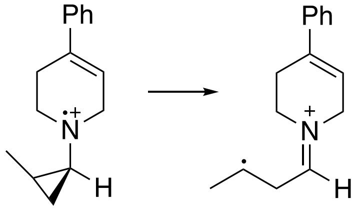

B2

	CAM3-B3LYP/CC-PVDZ		M062X/CC-PVDZ		M062X/DEF2-TZVP//M062X/CC-PVDZ	
	**au**	**kcal/mol**	**au**	**kcal/mol**	**au**	**kcal/mol**
reactant	-637.134087		-637.250344		-637.420566	
transition state ($\Delta E^{\neq}$)	-637.133225	0.5	-637.247497	1.8	-637.418162	1.5
product (ΔE^{o})	-637.138075	-2.5	-637.250409	0.04	-637.421838	-0.8

trans-**4b**$^{\bullet+}$

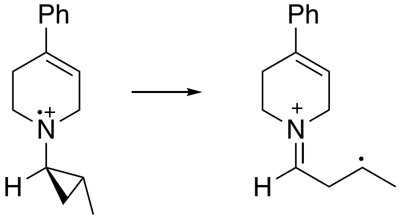

B1

	CAM3-B3LYP/CC-PVDZ		M062X/CC-PVDZ		M062X/DEF2-TZVP//M062X/CC-PVDZ	
	**au**	**kcal/mol**	**au**	**kcal/mol**	**au**	**kcal/mol**
reactant	-637.137877		-637.254137		-637.424603	
transition state ($\Delta E^{\neq}$)	-637.135397	1.5	-637.248805	3.3	-637.419471	3.2
product (ΔE^{o})	-637.139196	-0.8	-637.250478	2.3	-637.421745	1.8

trans-**4b**$^{\bullet+}$

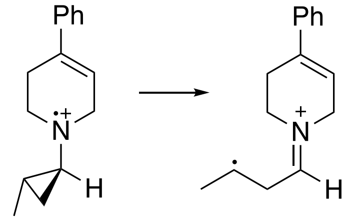

B2

	CAM3-B3LYP/CC-PVDZ		M062X/CC-PVDZ		M062X/DEF2-TZVP//M062X/CC-PVDZ	
	**au**	**kcal/mol**	**au**	**kcal/mol**	**au**	**kcal/mol**
reactant	-637.137889		-637.254146		-637.424695	
transition state ($\Delta E^{\neq}$)	-637.134112	2.4	-637.247734	4.0	-637.418649	3.7
product (ΔE^{o})	-637.138551	-0.4	-637.249993	2.6	-637.421317	2.1

XYZ coordinates for optimized structures pertinent to Table 3. Geometry optimizations were performed at the M06-2X/CC-PVDZ level

**Structure**	**XYZ coordinates**			
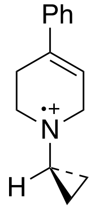 **4a**$^{\bullet+}$ (B1)	6	-0.036018	0.185857	1.394840
	1	0.538915	-0.103814	2.285791
	1	-0.040389	1.286959	1.360712
	6	-1.465375	-0.338538	1.563111
	1	-1.440303	-1.421253	1.759049
	1	-2.007720	0.167472	2.368875
	6	-1.667133	-0.906366	-0.813869
	1	-2.072629	-0.498730	-1.748438
	6	-0.158243	-0.890482	-0.831135
	1	0.289752	-1.298564	-1.738156
	6	0.605238	-0.389309	0.151543
	1	-2.054293	-1.938336	-0.716913
	7	-2.182310	-0.143320	0.311421
	6	2.086237	-0.371323	0.073393
	6	4.885611	-0.299175	-0.085199
	6	2.813061	0.629999	0.732116
	6	2.784929	-1.347717	-0.650955
	6	4.172971	-1.310662	-0.729759
	6	4.202429	0.670018	0.646110
	1	2.296520	1.402376	1.304271
	1	2.241842	-2.162965	-1.131155
	1	4.702954	-2.082838	-1.287395
	1	4.752306	1.461362	1.155220
	1	5.973670	-0.273235	-0.145332
	6	-3.161135	0.827305	0.200025
	6	-3.181575	1.773210	-1.037752
	6	-4.230871	0.753378	-0.904586
	1	-3.450692	1.260787	1.155476
	1	-2.375835	1.623842	-1.757302
	1	-3.399750	2.808531	-0.776141
	1	-4.200366	-0.129612	-1.542949
	1	-5.221796	1.055142	-0.564916
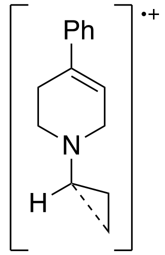 **4a**$^{\bullet+}$ (B1 transition state)	6	-0.044387	0.238773	1.374274
	1	0.552481	0.029564	2.272836
	1	-0.066035	1.334120	1.256148
	6	-1.453069	-0.291398	1.609821
	1	-1.415283	-1.349682	1.906506
	1	-1.988353	0.272168	2.382383
	6	-1.686339	-1.036048	-0.732139
	1	-2.094853	-0.672759	-1.683277
	6	-0.183117	-0.986130	-0.767094
	1	0.256846	-1.446291	-1.652273
	6	0.592589	-0.413008	0.166242
	1	-2.050260	-2.069906	-0.598501

	7	-2.229188	-0.226428	0.363313
	6	2.072481	-0.380573	0.064637
	6	4.874343	-0.280815	-0.128394
	6	2.805394	0.596225	0.754448
	6	2.771795	-1.318311	-0.711292
	6	4.157582	-1.266799	-0.808894
	6	4.194142	0.648526	0.654825
	1	2.295341	1.340040	1.368125
	1	2.234445	-2.118684	-1.221026
	1	4.683614	-2.009518	-1.408849
	1	4.744929	1.418851	1.194497
	1	5.961001	-0.245491	-0.201438
	6	-3.278655	0.570025	0.251216
	6	-3.188335	1.956450	-1.097750
	6	-4.092035	0.788937	-0.976745
	1	-3.588929	1.072313	1.168621
	1	-2.247845	1.869401	-1.642134
	1	-3.452362	2.918239	-0.660493
	1	-4.005716	0.025325	-1.752130
	1	-5.132223	1.032883	-0.745462
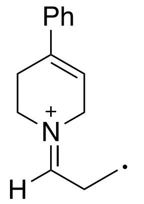 **4a**$^{\bullet+}$ (B1 product)	6	-0.014727	0.156529	1.414775
	1	0.590437	-0.086953	2.299341
	1	-0.054715	1.255822	1.349402
	6	-1.412103	-0.403955	1.624970
	1	-1.368640	-1.482326	1.833425
	1	-1.953629	0.097305	2.435040
	6	-1.652412	-1.006632	-0.762679
	1	-2.055712	-0.579529	-1.687981
	6	-0.149260	-0.963196	-0.784113
	1	0.296578	-1.378707	-1.689155
	6	0.622309	-0.433151	0.176356
	1	-2.028303	-2.040178	-0.678701
	7	-2.194208	-0.248293	0.381242
	6	2.101466	-0.401470	0.080201
	6	4.899436	-0.309840	-0.104593
	6	2.825482	0.624099	0.704445
	6	2.802573	-1.391163	-0.623303
	6	4.189807	-1.344562	-0.715113
	6	4.213869	0.673796	0.605161
	1	2.306496	1.407175	1.259733
	1	2.262507	-2.224098	-1.076120
	1	4.721514	-2.127521	-1.255799
	1	4.761092	1.483834	1.087173
	1	5.986702	-0.276656	-0.174611
	6	-3.223722	0.522954	0.326897
	6	-3.468236	2.184859	-1.315343
	6	-4.031194	0.863975	-0.880991

	1	-3.478141	1.033003	1.262373
	1	-2.516138	2.214895	-1.845951
	1	-3.934772	3.120024	-1.009676
	1	-3.963934	0.096954	-1.662209
	1	-5.080423	0.963006	-0.574196
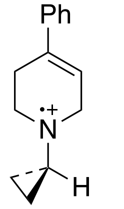 **4a**$^{\bullet+}$ (B2)	6	-0.288733	0.043194	1.000722
	1	0.495017	0.698215	1.415313
	1	-1.187505	0.212196	1.609009
	6	0.095289	-1.429011	1.152681
	1	0.393987	-1.676474	2.176676
	1	-0.763949	-2.061523	0.869902
	6	0.900261	-1.530719	-1.182064
	1	0.472566	-2.469712	-1.586579
	6	-0.025863	-0.372358	-1.416496
	1	-0.241331	-0.163582	-2.465747
	6	-0.543197	0.393204	-0.444839
	1	1.864788	-1.378866	-1.689945
	7	1.171958	-1.757316	0.231011
	6	-1.336567	1.609148	-0.749821
	6	-2.844527	3.887388	-1.387976
	6	-2.466713	1.950928	0.006341
	6	-0.965777	2.434792	-1.821393
	6	-1.714560	3.563869	-2.138900
	6	-3.218041	3.078795	-0.315662
	1	-2.792750	1.320636	0.835409
	1	-0.065569	2.204294	-2.393452
	1	-1.408733	4.199842	-2.969854
	1	-4.102344	3.324272	0.272443
	1	-3.430737	4.772515	-1.635186
	6	2.377985	-2.319169	0.618404
	6	3.004915	-1.965275	1.985706
	6	2.433890	-3.308024	1.807128
	1	3.058782	-2.497638	-0.212198
	1	2.464048	-1.230590	2.583146
	1	4.086022	-1.832867	1.939965
	1	1.477917	-3.546486	2.274141
	1	3.099472	-4.153679	1.632116
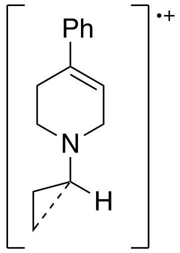 **4a**$^{\bullet+}$ (B2 transition state)	6	-0.225388	0.468833	0.966536
	1	0.360503	-0.289538	1.511093
	1	-0.067880	1.414356	1.501783
	6	-1.717143	0.151632	1.021440
	1	-2.057074	-0.055786	2.039953
	1	-2.294513	1.002609	0.630080
	6	-1.707053	-0.772211	-1.267995
	1	-2.565519	-0.249056	-1.723329
	6	-0.442839	0.018773	-1.452172
	1	-0.125269	0.135009	-2.489659

	6	0.267809	0.570832	-0.457857
	1	-1.628511	-1.756008	-1.751159
	7	-2.018873	-0.991445	0.153876
	6	1.548869	1.276796	-0.712818
	6	3.943646	2.639370	-1.257102
	6	1.895812	2.429303	0.006297
	6	2.430652	0.810101	-1.699413
	6	3.615694	1.487059	-1.971190
	6	3.080922	3.107419	-0.267830
	1	1.227425	2.831252	0.768601
	1	2.196760	-0.106695	-2.242291
	1	4.291364	1.107861	-2.737703
	1	3.328151	4.008878	0.292243
	1	4.872522	3.168784	-1.468324
	6	-2.506532	-2.151030	0.562741
	6	-1.501493	-3.142314	1.887668
	6	-2.849795	-2.534896	1.960259
	1	-2.714615	-2.875052	-0.226490
	1	-0.627059	-2.572286	2.199892
	1	-1.362584	-4.168322	1.549692
	1	-2.969710	-1.716697	2.672941
	1	-3.678284	-3.247532	2.000237
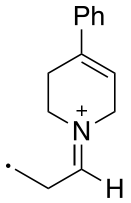<br>**4a**$^{\bullet +}$ (B2 product)	6	-0.570299	-0.344598	0.916199
	1	0.177723	0.229654	1.486976
	1	-1.528030	-0.200003	1.433887
	6	-0.246992	-1.833560	0.948348
	1	-0.043297	-2.199749	1.957352
	1	-1.074928	-2.415840	0.520630
	6	0.621329	-1.825825	-1.347414
	1	0.034785	-2.682805	-1.716784
	6	-0.124724	-0.530909	-1.512207
	1	-0.227878	-0.184182	-2.541971
	6	-0.646041	0.178069	-0.499792
	1	1.574792	-1.811306	-1.891789
	7	0.916754	-2.079068	0.077549
	6	-1.289868	1.496300	-0.722398
	6	-2.525795	3.971454	-1.191524
	6	-2.432814	1.871645	-0.002693
	6	-0.767997	2.387371	-1.671713
	6	-1.382397	3.613702	-1.905679
	6	-3.048768	3.097391	-0.240393
	1	-2.874868	1.196156	0.731046
	1	0.143417	2.127352	-2.212700
	1	-0.960604	4.297933	-2.641988
	1	-3.944227	3.368616	0.318627
	1	-3.005944	4.933021	-1.373218
	6	2.098100	-2.398656	0.475437

	6	3.390072	-1.334007	2.143240
	6	2.574441	-2.565603	1.880949
	1	2.852771	-2.487853	-0.313240
	1	2.898064	-0.414441	2.458711
	1	4.455700	-1.314966	1.919214
	1	1.754985	-2.678394	2.599538
	1	3.202535	-3.466897	1.917799

Structure	XYZ coordinates
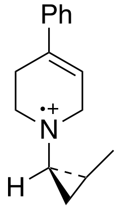	6 0.552826 -1.396855 -0.213857
cis-**4b**$^{\bullet+}$ (B1)	1 -0.315842 -1.570635 -0.870444
	1 1.328892 -2.098219 -0.549295
	6 0.217375 -1.739572 1.234047
	1 -0.269447 -2.716670 1.328173
	1 1.140691 -1.743741 1.837279
	6 -0.125775 0.631714 1.806795
	1 0.453149 0.745800 2.744980
	6 0.730248 0.918517 0.606986
	1 1.105780 1.941488 0.550585
	6 1.025061 0.029816 -0.350181
	1 -0.952662 1.349576 1.873196
	7 -0.662990 -0.722467 1.805622
	6 1.784711 0.410485 -1.567126
	6 3.241912 1.167594 -3.841657
	6 2.747451 -0.445631 -2.117891
	6 1.557261 1.648192 -2.184146
	6 2.279772 2.023771 -3.310413
	6 3.473606 -0.067332 -3.241907
	1 2.960742 -1.410473 -1.655026
	1 0.787246 2.313923 -1.791107
	1 2.084357 2.987774 -3.782165
	1 4.227810 -0.741439 -3.649990
	1 3.808095 1.461313 -4.726664
	6 -1.861917 -1.089716 2.361842
	6 -2.800045 -0.151003 3.086201
	6 -3.291834 -0.563207 1.758564
	1 -1.913659 -2.152423 2.598024
	1 -2.476208 0.883493 3.208168
	1 -3.263879 -0.584441 3.975061
	1 -3.979323 -1.412632 1.738311
	6 -3.362088 0.344071 0.567947
	1 -4.367429 0.793916 0.538950
	1 -3.226824 -0.211697 -0.370570
	1 -2.637061 1.166711 0.601709
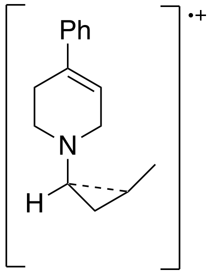	6 0.579218 -1.419224 -0.146267
	1 -0.301007 -1.609252 -0.782354
	1 1.357639 -2.109630 -0.497733
	6 0.278302 -1.756696 1.308686
	1 -0.198718 -2.737607 1.415462
	1 1.210351 -1.755549 1.894952
	6 -0.041926 0.612821 1.901887
cis-**4b**$^{\bullet+}$ (B1 transition state)	1 0.589852 0.704815 2.804930
	6 0.752895 0.905467 0.660667
	1 1.118691 1.930597 0.586357
	6 1.026982 0.015055 -0.302307

	1	-0.852465	1.342291	2.017147
	7	-0.603521	-0.739762	1.893629
	6	1.748345	0.401026	-1.541481
	6	3.142079	1.171461	-3.854046
	6	2.706773	-0.444377	-2.115515
	6	1.492835	1.634722	-2.155892
	6	2.183790	2.017024	-3.300244
	6	3.401157	-0.059713	-3.257882
	1	2.943652	-1.404971	-1.656534
	1	0.726242	2.292901	-1.744876
	1	1.966314	2.978045	-3.767524
	1	4.152672	-0.726053	-3.682408
	1	3.683296	1.469940	-4.752362
	6	-1.789127	-1.084114	2.410201
	6	-2.813427	-0.163324	2.983182
	6	-3.354603	-0.506348	1.650497
	1	-1.933619	-2.156172	2.543940
	1	-2.510637	0.874824	3.132762
	1	-3.340332	-0.581041	3.846142
	1	-3.997742	-1.387331	1.587086
	6	-3.312690	0.391478	0.465284
	1	-4.293859	0.892587	0.384873
	1	-3.167936	-0.169914	-0.468378
	1	-2.549475	1.176025	0.538122
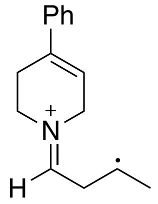<br>*cis*-**4b**$^{•+}$ (B1 product)	6	0.814431	-1.497118	0.016617
	1	-0.012484	-1.826817	-0.634282
	1	1.682090	-2.108502	-0.267745
	6	0.499932	-1.787869	1.475864
	1	0.108095	-2.800775	1.626440
	1	1.397619	-1.661149	2.098420
	6	-0.010349	0.570000	1.966646
	1	0.629208	0.697792	2.857042
	6	0.738714	0.875388	0.700251
	1	0.986989	1.929277	0.562387
	6	1.097544	-0.032234	-0.217614
	1	-0.863971	1.247320	2.079392
	7	-0.498065	-0.821050	1.975042
	6	1.749072	0.361135	-1.492076
	6	2.992870	1.143238	-3.882296
	6	2.788990	-0.399943	-2.039459
	6	1.336092	1.515000	-2.170675
	6	1.952735	1.903122	-3.354262
	6	3.409099	-0.008334	-3.220960
	1	3.146690	-1.296722	-1.531050
	1	0.504147	2.102575	-1.779018
	1	1.612173	2.800586	-3.872616
	1	4.226047	-0.607145	-3.625862

	1	3.476117	1.446073	-4.812176
	6	-1.674093	-1.197541	2.346620
	6	-2.826454	-0.336244	2.726908
	6	-3.659608	-0.307387	1.473750
	1	-1.856744	-2.276090	2.291139
	1	-2.523978	0.668501	3.049188
	1	-3.368393	-0.822890	3.550503
	1	-4.408580	-1.090810	1.341158
	6	-3.415472	0.695220	0.406747
	1	-3.493281	1.725049	0.801189
	1	-4.135590	0.590474	-0.414463
	1	-2.401592	0.607871	-0.030749
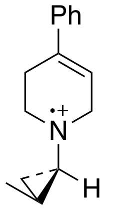 *cis*-**4b**$^{\bullet+}$ (B2)	6	0.287358	-0.854250	-0.631252
	1	-0.531579	-1.176729	-1.294013
	1	1.154029	-1.481139	-0.880130
	6	-0.066983	-1.122608	0.832268
	1	-0.397059	-2.153133	0.983076
	1	0.816226	-0.927635	1.463976
	6	-0.765682	1.217890	1.163073
	1	-0.277543	1.497528	2.117199
	6	0.130165	1.524288	-0.002909
	1	0.382842	2.578996	-0.125426
	6	0.593769	0.604870	-0.860354
	1	-1.701991	1.792136	1.105992
	7	-1.110767	-0.199246	1.254154
	6	1.388800	0.979154	-2.056021
	6	2.916413	1.726292	-4.287042
	6	2.500361	0.219645	-2.446342
	6	1.047474	2.112995	-2.807283
	6	1.806115	2.483372	-3.914146
	6	3.261974	0.594705	-3.549913
	1	2.802882	-0.656894	-1.871328
	1	0.163991	2.693331	-2.536317
	1	1.523602	3.362409	-4.493475
	1	4.131421	0.001582	-3.833047
	1	3.509402	2.015789	-5.154566
	6	-2.329048	-0.554346	1.780607
	6	-2.840790	-1.973801	1.911203
	6	-2.400550	-1.370295	3.183027
	1	-3.060650	0.252391	1.750278
	1	-2.225603	-2.771025	1.493199
	1	-3.909770	-2.066886	1.714657
	1	-3.172889	-0.913790	3.806208
	6	-1.140853	-1.771050	3.891909
	1	-1.406718	-2.482335	4.688462
	1	-0.656186	-0.909292	4.371804
	1	-0.422162	-2.272920	3.232471

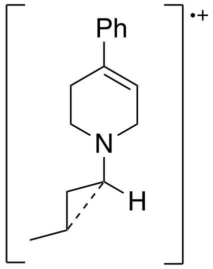 *cis*-**4b**$^{\bullet+}$ (B2 transition state)	6	0.353013	-0.858549	-0.625052
	1	-0.450986	-1.202528	-1.295512
	1	1.236656	-1.464754	-0.865592
	6	-0.014294	-1.124016	0.831521
	1	-0.343502	-2.155933	0.981920
	1	0.855569	-0.930976	1.478928
	6	-0.681898	1.218272	1.194530
	1	-0.145289	1.454413	2.131251
	6	0.171369	1.526106	-0.003555
	1	0.412359	2.581929	-0.141135
	6	0.628705	0.606779	-0.865410
	1	-1.600604	1.822175	1.185072
	7	-1.072013	-0.197106	1.240435
	6	1.395239	0.986377	-2.079142
	6	2.872301	1.738441	-4.344866
	6	2.501527	0.232209	-2.494276
	6	1.034003	2.118197	-2.824630
	6	1.767506	2.491139	-3.947669
	6	3.237422	0.608741	-3.614620
	1	2.820216	-0.643495	-1.926953
	1	0.154200	2.695646	-2.536006
	1	1.468992	3.369067	-4.520872
	1	4.102267	0.017937	-3.916393
	1	3.444827	2.029690	-5.225513
	6	-2.296278	-0.541629	1.625822
	6	-2.796409	-1.920048	1.890640
	6	-2.516605	-1.458770	3.271525
	1	-3.009736	0.280258	1.700076
	1	-2.173861	-2.743388	1.533812
	1	-3.852644	-2.042313	1.635713
	1	-3.315241	-0.941119	3.806226
	6	-1.278577	-1.805683	4.015645
	1	-1.527317	-2.592503	4.749769
	1	-0.891815	-0.952060	4.590006
	1	-0.489102	-2.202504	3.364303
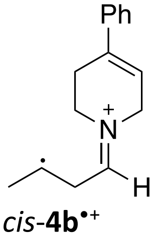 *cis*-**4b**$^{\bullet+}$ (B2 product)	6	0.436104	-0.790487	-0.471571
	1	-0.343891	-1.213876	-1.125198
	1	1.342606	-1.384544	-0.649559
	6	0.056201	-0.948795	0.994586
	1	-0.258098	-1.965482	1.244090
	1	0.903774	-0.674906	1.639807
	6	-0.592988	1.409574	1.216493
	1	-0.014700	1.641699	2.126401
	6	0.221640	1.635981	-0.026642
	1	0.440585	2.681204	-0.252184
	6	0.673049	0.658828	-0.827326
	1	-1.493010	2.039050	1.222545

	7	-1.024024	0.001480	1.312842	
	6	1.395761	0.957020	-2.088812	
	6	2.788132	1.565768	-4.447603	
	6	2.496230	0.185983	-2.487803	
	6	0.995792	2.030344	-2.897817	
	6	1.687371	2.332690	-4.066947	
	6	3.190208	0.492271	-3.655104	
	1	2.844322	-0.645300	-1.872924	
	1	0.117963	2.616512	-2.621349	
	1	1.359523	3.165479	-4.689317	
	1	4.051824	-0.109257	-3.944400	
	1	3.328601	1.801306	-5.364564	
	6	-2.246294	-0.317362	1.582067	
	6	-2.775897	-1.686231	1.835385	
	6	-2.669623	-1.760984	3.333589	
	1	-2.927104	0.523855	1.747220	
	1	-2.202417	-2.475878	1.333536	
	1	-3.820227	-1.731272	1.503503	
	1	-3.523017	-1.430496	3.926633	
	6	-1.375557	-2.087253	3.986653	
	1	-0.928476	-2.999949	3.557318	
	1	-1.502840	-2.241022	5.065058	
	1	-0.631523	-1.275951	3.861393	

Structure	XYZ coordinates			
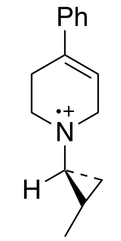 *trans*-**4b**$^{•+}$ (B1)	1	0.520406	0.193876	-1.884431
	6	-0.396279	0.501983	-1.357018
	1	-1.117961	0.814999	-2.124525
	6	-0.107334	1.705904	-0.460632
	1	-1.055115	2.099814	-0.059656
	1	0.423710	2.505428	-0.988064
	7	0.697719	1.276295	0.676294
	6	0.068637	0.298219	1.550564
	1	-0.563641	0.854713	2.269623
	1	0.844190	-0.211956	2.137261
	6	-0.749103	-0.699969	0.774336
	1	-1.138722	-1.527353	1.369198
	6	-0.963066	-0.642538	-0.548324
	6	-1.745547	-1.675286	-1.269069
	6	-3.184859	-3.654273	-2.639377
	6	-1.454990	-1.970582	-2.608221
	6	-2.782821	-2.371688	-0.632651
	6	-3.495614	-3.353471	-1.312855
	6	-2.164002	-2.959824	-3.285371
	1	-0.654427	-1.442070	-3.128474
	1	-3.056959	-2.122263	0.393557
	1	-4.306462	-3.879055	-0.808679
	1	-1.918157	-3.187153	-4.322541
	1	-3.745064	-4.422913	-3.171846
	6	1.960361	1.785126	0.885574
	6	2.514215	1.968182	2.344418
	6	3.098406	0.926975	1.489749
	1	2.250497	2.547151	0.163170
	1	1.803906	1.628914	3.102720
	1	4.062388	1.118329	1.014956
	1	2.860284	-0.121668	1.668475
	6	3.230296	3.261412	2.627813
	1	3.859981	3.133542	3.520364
	1	2.520985	4.075699	2.821758
	1	3.884144	3.545503	1.791902
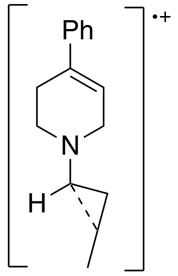 *trans*-**4b**$^{•+}$ (B1 transition state)	1	0.549379	0.035410	-2.011997
	6	-0.300398	0.347257	-1.383750
	1	-1.061773	0.762110	-2.059093
	6	0.132669	1.442440	-0.423275
	1	-0.746567	1.858840	0.093897
	1	0.658082	2.258006	-0.933848
	7	1.028118	0.897851	0.607197
	6	0.489638	-0.207423	1.406892
	1	0.028138	0.209360	2.321681
	1	1.328773	-0.842505	1.722726
	6	-0.501167	-1.039797	0.644361

	1	-0.881640	-1.899373	1.198592
	6	-0.866643	-0.832985	-0.628381
	6	-1.808121	-1.729353	-1.341141
	6	-3.546103	-3.452877	-2.713894
	6	-1.714412	-1.880622	-2.731413
	6	-2.800063	-2.441235	-0.651468
	6	-3.660233	-3.296021	-1.332301
	6	-2.571742	-2.742131	-3.411280
	1	-0.951812	-1.338950	-3.293694
	1	-2.921478	-2.302422	0.423936
	1	-4.432696	-3.834758	-0.783217
	1	-2.477531	-2.857481	-4.491098
	1	-4.222440	-4.121607	-3.246674
	6	2.190073	1.480831	0.865754
	6	2.417306	2.369871	2.556277
	6	3.093494	1.168164	2.005823
	1	2.514637	2.232918	0.144638
	1	1.503007	2.207992	3.131327
	1	4.151098	1.308285	1.762712
	1	2.896228	0.231671	2.531780
	6	2.998968	3.733545	2.493280
	1	3.466224	3.955461	3.470222
	1	2.234301	4.503838	2.325430
	1	3.784687	3.815691	1.729997
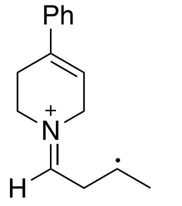 *trans*-**4b**$^{\bullet+}$ (B1 product)	1	0.376409	0.151563	-1.999966
	6	-0.499923	0.411556	-1.384557
	1	-1.277137	0.769488	-2.074457
	6	-0.152285	1.542042	-0.430584
	1	-1.057173	1.906855	0.077480
	1	0.338181	2.383274	-0.933319
	7	0.752065	1.043395	0.623891
	6	0.161770	-0.008510	1.469616
	1	-0.423625	0.492746	2.260140
	1	0.972160	-0.562123	1.956690
	6	-0.691801	-0.950263	0.667182
	1	-1.029766	-1.828728	1.218892
	6	-0.999397	-0.798960	-0.629029
	6	-1.818903	-1.786376	-1.371423
	6	-3.336617	-3.671278	-2.790490
	6	-1.639759	-1.954981	-2.751185
	6	-2.784763	-2.563809	-0.717012
	6	-3.536111	-3.498527	-1.420649
	6	-2.387954	-2.896385	-3.453557
	1	-0.896153	-1.363517	-3.288136
	1	-2.974185	-2.414967	0.347191
	1	-4.290788	-4.087597	-0.899143
	1	-2.228359	-3.023205	-4.524422

	1	-3.926959	-4.403364	-3.341891
	6	1.940537	1.528559	0.776858
	6	2.679753	2.486713	2.721295
	6	2.894376	1.253519	1.888061
	1	2.226331	2.305222	0.060154
	1	1.823379	2.490714	3.397831
	1	3.917074	1.214366	1.489000
	1	2.681415	0.328053	2.434462
	6	3.599683	3.648883	2.658058
	1	4.444932	3.499905	3.356857
	1	3.101522	4.582457	2.948472
	1	4.042073	3.773283	1.657691
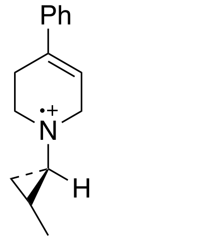	1	0.446808	0.135768	-1.376125
	6	-0.468824	0.464034	-0.857666
	1	-1.177040	0.785501	-1.633956
	6	-0.168756	1.672236	0.028328
	1	-1.113098	2.081895	0.424208
	1	0.354003	2.463583	-0.518161
trans-**4b**$^{\bullet+}$ (B2)	7	0.625291	1.258101	1.175599
	6	0.027386	0.241711	2.033890
	1	-0.563975	0.767897	2.808456
	1	0.842586	-0.279449	2.556644
	6	-0.829797	-0.728535	1.271808
	1	-1.232218	-1.544632	1.873631
	6	-1.060377	-0.666780	-0.047476
	6	-1.873175	-1.678963	-0.762933
	6	-3.364147	-3.625246	-2.126162
	6	-1.615766	-1.961019	-2.111429
	6	-2.903755	-2.372967	-0.112912
	6	-3.641699	-3.338357	-0.789350
	6	-2.350424	-2.933240	-2.785525
	1	-0.820880	-1.435380	-2.643133
	1	-3.151712	-2.135452	0.922569
	1	-4.445957	-3.862666	-0.273453
	1	-2.129852	-3.149558	-3.830758
	1	-3.943619	-4.381706	-2.655524
	6	1.869011	1.770100	1.479589
	6	2.889840	2.102878	0.325477
	6	2.279700	3.201106	1.089096
	1	2.303649	1.352103	2.386887
	1	2.471459	1.907765	-0.665308
	1	2.876943	3.693010	1.859181
	1	1.505547	3.816097	0.630098
	6	4.313378	1.668974	0.546136
	1	4.969474	2.248267	-0.120012
	1	4.448194	0.604088	0.318027
	1	4.631799	1.858835	1.580258

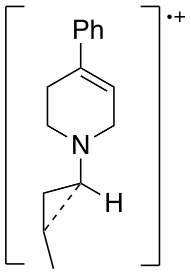 *trans*-**4b**$^{\bullet+}$ (B2 transition state)	1	0.197221	0.541331	-1.343917
	6	-0.701583	0.715686	-0.730197
	1	-1.505320	0.990418	-1.427635
	6	-0.476394	1.885284	0.224369
	1	-1.428202	2.167087	0.698691
	1	-0.076363	2.765504	-0.286525
	7	0.426999	1.483185	1.305414
	6	-0.093543	0.395538	2.145482
	1	-0.783154	0.837505	2.885427
	1	0.748631	-0.038544	2.700531
	6	-0.791086	-0.655163	1.326984
	1	-1.051620	-1.560873	1.876094
	6	-1.081156	-0.541293	0.022424
	6	-1.777788	-1.611795	-0.732373
	6	-3.076868	-3.644197	-2.170168
	6	-1.549816	-1.774527	-2.106076
	6	-2.682673	-2.473258	-0.094752
	6	-3.324953	-3.480699	-0.807060
	6	-2.188796	-2.787827	-2.817465
	1	-0.854994	-1.117845	-2.631650
	1	-2.912786	-2.334407	0.962194
	1	-4.033008	-4.134370	-0.298089
	1	-1.992476	-2.906106	-3.882830
	1	-3.583224	-4.431630	-2.727656
	6	1.641670	1.972070	1.513713
	6	2.975393	1.796934	0.130917
	6	2.338553	3.008688	0.705407
	1	2.147631	1.601668	2.406376
	1	2.486274	1.338127	-0.730605
	1	3.004841	3.631218	1.310071
	1	1.716412	3.597038	0.028658
	6	4.298029	1.283256	0.568719
	1	5.061880	1.631291	-0.150215
	1	4.337292	0.185959	0.568029
	1	4.584978	1.664746	1.557712
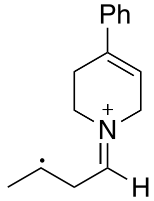 *trans*-**4b**$^{\bullet+}$ (B2 product)	1	0.151680	0.544050	-1.244552
	6	-0.769945	0.736696	-0.671384
	1	-1.543131	1.012207	-1.402541
	6	-0.560482	1.914758	0.272140
	1	-1.513305	2.213508	0.731133
	1	-0.127239	2.783477	-0.229663
	7	0.312806	1.485774	1.376478
	6	-0.317073	0.465084	2.235321
	1	-1.044764	0.982240	2.882404
	1	0.461577	0.038101	2.880596
	6	-0.976732	-0.599236	1.402498
	1	-1.264272	-1.496463	1.952258

	6	-1.192245	-0.506500	0.081518
	6	-1.833295	-1.593004	-0.698306
	6	-3.009610	-3.661389	-2.187679
	6	-1.546375	-1.745744	-2.061579
	6	-2.736257	-2.482455	-0.097521
	6	-3.317144	-3.507642	-0.835571
	6	-2.124414	-2.776509	-2.798768
	1	-0.851453	-1.067184	-2.558957
	1	-3.013529	-2.353914	0.949826
	1	-4.024647	-4.184250	-0.356001
	1	-1.882647	-2.886127	-3.856047
	1	-3.468283	-4.464197	-2.764996
	6	1.536591	1.857932	1.554720
	6	3.211446	1.718754	-0.027391
	6	2.356965	2.737222	0.675401
	1	2.046112	1.399047	2.408382
	1	2.784023	1.217483	-0.897342
	1	2.968992	3.398787	1.304391
	1	1.761808	3.346182	-0.014044
	6	4.595482	1.415589	0.414564
	1	5.302827	2.149338	-0.016934
	1	4.920387	0.419620	0.089752
	1	4.706147	1.488764	1.507895

Structure[a]	XYZ coordinates			
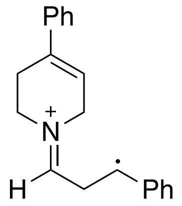 *trans*-**4c**$^{\bullet+}$ (B1 product)	1	-0.709415	-1.508770	-0.722071
	6	-1.729281	-1.293443	-0.375061
	1	-2.350326	-2.119567	-0.736979
	6	-2.246451	-0.005658	-1.018308
	1	-2.057138	0.024569	-2.093358
	1	-3.322867	0.105721	-0.850824
	7	-1.590306	1.165066	-0.390283
	6	-1.915404	1.305852	1.046293
	1	-2.910662	1.769325	1.112523
	1	-1.209290	2.001835	1.503801
	6	-1.873820	-0.027116	1.748236
	1	-1.945752	0.034155	2.830316
	6	-1.757578	-1.219539	1.139103
	6	-1.612788	-2.478094	1.912885
	6	-1.353059	-4.839830	3.423559
	6	-2.234714	-3.668426	1.499336
	6	-0.846143	-2.500070	3.091662
	6	-0.719405	-3.667578	3.840522
	6	-2.110352	-4.835750	2.251223
	1	-2.847193	-3.685351	0.602240
	1	-0.324242	-1.600754	3.406579
	1	-0.117204	-3.664265	4.744405
	1	-2.609905	-5.742068	1.921719
	1	-1.253187	-5.751333	4.005374
	6	-0.761131	1.918026	-1.056158
	6	0.131421	2.993772	-0.535160
	6	1.442319	2.252394	-0.505663
	1	-0.641105	1.664249	-2.107016
	1	-0.155246	3.372440	0.447968
	1	0.139149	3.834839	-1.234246
	1	1.596900	1.604681	0.353597
	6	2.426077	2.256044	-1.521710
	6	4.475015	2.212908	-3.464367
	6	3.584958	1.439162	-1.357146
	6	2.335879	3.049084	-2.703001
	6	3.345196	3.022668	-3.651781
	6	4.588294	1.423701	-2.310740
	1	3.674349	0.830531	-0.461025
	1	1.475265	3.691016	-2.867366
	1	3.261933	3.636239	-4.543784
	1	5.464420	0.799379	-2.163917
	1	5.262789	2.199956	-4.211377

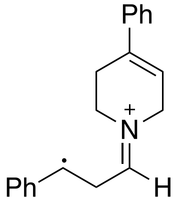 *trans*-**4c**$^{\bullet+}$ (B2 product)	1	-0.054954	0.399443	-2.583356
	6	-0.991691	0.031961	-2.142628
	1	-1.792917	0.404363	-2.789267
	6	-1.202172	0.643861	-0.754490
	1	-1.048445	1.721763	-0.759150
	1	-2.217609	0.439284	-0.397484
	7	-0.280912	0.008753	0.211212
	6	-0.536617	-1.441831	0.383722
	1	-1.386797	-1.546915	1.074441
	1	0.336117	-1.880066	0.879999
	6	-0.808772	-2.119069	-0.932314
	1	-0.859488	-3.202970	-0.878670
	6	-0.989698	-1.483696	-2.102533
	6	-1.157825	-2.223946	-3.377468
	6	-1.482928	-3.674757	-5.768679
	6	-2.052789	-1.786429	-4.368898
	6	-0.418561	-3.396524	-3.616395
	6	-0.581548	-4.115668	-4.797914
	6	-2.216913	-2.508160	-5.550127
	1	-2.654116	-0.894490	-4.215443
	1	0.310787	-3.728408	-2.882517
	1	0.004181	-5.015057	-4.965355
	1	-2.922183	-2.159135	-6.298893
	1	-1.607543	-4.233284	-6.691857
	6	0.680022	0.606446	0.855374
	6	0.963656	2.068683	0.940276
	6	0.405477	2.381836	2.304156
	1	1.290276	-0.036507	1.485924
	1	0.489700	2.659290	0.152972
	1	2.043076	2.233831	0.881145
	1	-0.675980	2.475157	2.361098
	6	1.143448	2.495479	3.504479
	6	2.513200	2.792835	5.956013
	6	0.439975	2.755978	4.718901
	6	2.563516	2.385227	3.571603
	6	3.228245	2.532281	4.777861
	6	1.115801	2.904918	5.917556
	1	-0.642694	2.847292	4.688551
	1	3.141059	2.190351	2.672719
	1	4.310414	2.448394	4.809844
	1	0.561825	3.109083	6.828837
	1	3.042377	2.910403	6.896755

[a]Neither conformation B1 nor B2 of *trans*-**4c**$^{\bullet+}$ (ring closed form) was found to reside at a potential energy minimum.

Reviewer comments (not directly addressed in the manuscript)

One reviewer of this manuscript made a number of comments and conjectures which, while interesting, would require additional experiments/data that go beyond the intended scope of this study. These are noted below for the interested reader:

Reviewer: To confirm the dihydropyridine intermediate hypothesis, an experiment with 6,6-dimethyl-4 would stop at that stage upon exposure to oxygen.

Response: We do not have the means to synthesize the proposed compound, or run such experiments. However, there is ample evidence in the cited papers (Castagnoli et. al) for a dihydropyridine intermediate enroute to the final pyridinium product. It also should be noted that this hypothesis is not critical to the main point of the paper, but does explain some minor peaks noted under anaerobic conditions that vanished upon addition of O_2.

Comment: The premise of the paper is that the double bond in MPTP is responsible for the tertiary amine activity with MAOB, yet the MPTP analogue without the double bond as a substrate/inactivator was not made and tested. If the double bond is lowering the pKa of the amine, then the 5-fluoro MPTP analogue should increase activity.

The reviewer correctly notes the overarching point of the paper. The compound without the C=C is not an MAO substrate or inhibitor and we think we know why as articulated in the manuscript. The effect of substituent is an incredibly good, albeit complicated question. To my knowledge, no one has looked at the fluoro compound.

However, I actually disagree and do not think that the 5-fluoro analog would be more reactive. Earlier work has shown that in these tetrahydropyridines, the C-H BDEs are practically independent of substituent (*Chem. Thermodyn. Therm. Anal.* **2023,** *12*, 100119). In the context of a thermochemical cycle, the bond dissociation process (R-H → R• + H•) is the sum of electron transfer (R-H → $RH^{\bullet+}$ + e^-), deprotonation ($RH^{\bullet+}$ → R• + H^+), and of course H^+ + e^- → H•, which is constant across the series. Since BDE does not seem to vary with substituent, this suggests that any substituent such as fluorine which makes the deprotonation more favorable (presumably by inductive/field effects), would make the electron transfer step <u>less</u> favorable. This makes sense chemically as it implies that substituents have much more of an effect on the energy of the radical cation than on either the neutral compound or radical.

And to the extent that electron transfer seems to be rate limiting, as we believe, that would mean the fluoro compound would be less reactive. Also, in the case of MPTP, the electron removed from the nitrogen lone pair (not the aromatic ring), so any substituents on the "northern" end of the molecule would have to exert their effect over a fairly long distance.

Thus while the question is intriguing, complicated, and something we thought about as part of an extended study of substituent effects on reactivity, this issue goes well beyond the intended scope of this paper which is to document the behavior of N-cyclopropyl containing compounds related to MPTP.
